# Stochastic phenotype switching leads to intratumor heterogeneity in human liver cancer

**DOI:** 10.1002/hep.29679

**Published:** 2018-02-01

**Authors:** Andrija Matak, Pooja Lahiri, Ethan Ford, Daniela Pabst, Karl Kashofer, Dimitris Stellas, Dimitris Thanos, Kurt Zatloukal

**Affiliations:** ^1^ Institute of Pathology Medical University of Graz Graz Austria; ^2^ University of Western Australia Crawley WA Australia; ^3^ Biomedical Research Foundation Academy of Athens Athens Greece

## Abstract

Intratumor heterogeneity is increasingly recognized as a major factor impacting diagnosis and personalized treatment of cancer. We characterized stochastic phenotype switching as a mechanism contributing to intratumor heterogeneity and malignant potential of liver cancer. Clonal analysis of primary tumor cell cultures of a human sarcomatoid cholangiocarcinoma identified different types of self‐propagating subclones characterized by stable (keratin‐7‐positive or keratin‐7‐negative) phenotypes and an unstable phenotype consisting of mixtures of keratin‐7‐positive and keratin‐7‐negative cells, which lack stem cell features but may reversibly switch their phenotypes. Transcriptome sequencing and immunohistochemical studies with the markers Zeb1 and CD146/MCAM demonstrated that switching between phenotypes is linked to changes in gene expression related but not identical to epithelial–mesenchymal transition. Stochastic phenotype switching occurred during mitosis and did not correlate with changes in DNA methylation. Xenotransplantation assays with different cellular subclones demonstrated increased tumorigenicity of cells showing phenotype switching, resulting in tumors morphologically resembling the invasive component of primary tumor and metastasis. *Conclusion*. Our data demonstrate that stochastic phenotype switching contributes to intratumor heterogeneity and that cells with a switching phenotype have increased malignant potential. (Hepatology 2017).

**Abbreviations**
5‐aza‐dC: 5‐aza‐2′‐deoxycytidine
BrdU: bromodeoxyuridine
DEG: differentially expressed gene
DMR: differentially methylated region
EMT: epithelial–mesenchymal transition
FACS: fluorescence‐activated cell sorting
MeDIP‐seq: methylated DNA immunoprecipitation sequencing
RNA‐seq: RNA sequencing

Intratumor heterogeneity is a common but poorly understood phenomenon of most cancers. Heterogeneity within a primary tumor may include diversity in cell morphology and biological behavior relating to proliferation, metastatic potential, and responsiveness to targeted therapies. As a consequence, intratumor heterogeneity has become particularly important in the context of personalized medicine where it can markedly influence the relevance of diagnostic and prognostic biomarker analysis, sample size bias in clinical trials, and treatment failure associated with selective responses of cancer cell subpopulations to drugs.^1^, ^2^, ^3^, ^4^ Insight into the mutational and gene expression heterogeneity in different tumor regions is considered to provide a gateway for understanding the biological impact of genetic and phenotypic diversity within a primary tumor and its metastases.^5^, ^6^

Several mechanisms may be responsible for intratumor heterogeneity.^7^, ^8^, ^9^ The clonal evolution model underscores the contribution of stochastic chromosomal aberrations, gene mutations, or persistent epigenetic abnormalities in generating distinct subclones within a tumor.^5^, ^10^, ^11^ Alternatively, intratumor heterogeneity could be due to physiological cell differentiation mechanisms operating in stem or progenitor cells. For instance, if an initial genetic aberration affects a stem or progenitor cell, then the developing tumor may consist of a mixture of cells reflecting the differentiation potential of the primary affected cell.^12^, ^13^ This type of intratumor heterogeneity is specifically exemplified in a rare liver cancer subtype classified as hepatocellular‐cholangiocarcinoma, which is formed by coexisting malignant hepatocyte and cholangiocyte cell lineages, consistent with the notion that the cell of origin could have been a bipotential liver progenitor cell.^14^, ^15^

The cellular organization of tumors may also be affected by the type of driver mutations and the differentiation status of the targeted cells and is often influenced by factors secreted from cells forming the microenvironment surrounding the tumor.^16^, ^17^ In this context, epithelial–mesenchymal transition (EMT) changes may be involved in generating phenotypically distinct tumor cell populations. EMT may also lead to the conversion of nontumorigenic cells to cancer stem cells,^12^, ^18^ thus facilitating the metastatic spread of solid tumors during tumor progression.^19^

Alternatively, intratumor heterogeneity could be due to epigenetic events, such as DNA methylation and/or histone modifications. According to this model, the unifying feature of diverse tumor types involves loss of epigenetic stability to promote intratumor heterogeneity.^20^, ^21^ Importantly, as epigenetic and genetic defects may influence each other, they could synergistically affect tumorigenesis, resulting in the generation of unique cancer phenotypes.^22^ Recent studies have shown that abnormal methylation is confined to large hypomethylated chromosomal regions, which involve genes implicated in tissue differentiation, epigenetic reprogramming, and cancer occurrence.^23^, ^24^ Furthermore, stochastic epigenetic variation is an inherent characteristic of the phenotypic variability implicated in normal cell differentiation as well as in cancers where cellular diversity favors the selection of the fittest cancer clones influenced by a changing tumor environment.^25^, ^26^

Sarcomatoid cholangiocarcinoma is a rare type of liver cancer characterized by coinciding epithelial cholangiocytic and mesenchymal (sarcomatoid) differentiated cells in regionally distinct as well as phenotypically mixed, transitional tumor compartments.^27^, ^28^ This tumor type can be considered particularly informative for elucidating cellular, histological, and molecular intratumor heterogeneity. The existence of sarcomatoid features in tumors is generally associated with a more aggressive tumor behavior, leading to early patient demise after initial diagnosis.^29^, ^30^, ^31^ Recent studies have established a divergent, monoclonal progression model of sarcomatoid carcinomas, where unstable epithelial cells undergo a trans‐differentiation process from epithelial toward mesenchymal differentiation.^32^, ^33^, ^34^ In our study, we found that stochastic phenotype switching provides a mechanism leading to intratumor heterogeneity in a case of sarcomatoid cholangiocarcinoma from which primary cultures were established, recapitulating the heterogeneity in differentiation within the primary human tumor and its metastasis (for an overview, see http://onlinelibrary.wiley.com/doi/10.1002/hep.29679/suppinfo).

## Materials and Methods

### PRIMARY TUMOR AND CELL CULTURE

Primary tumor cell culture was established from fresh tissue originating from a surgically resected sarcomatoid cholangiocarcinoma of the liver. The study was approved by the research ethics committee of the Medical University of Graz (12‐159 ex 01/02 and EK20‐119). Tumor tissue was mechanically cut into small slices, which were placed into culture dishes in Dulbecco's modified Eagle's medium supplemented with 20% fetal bovine serum and 1% penicillin/streptomycin and cultured at 37°C in 5% CO_2_ for 1 month. Outgrowing cells were detached by mechanically scraping and transferring to six‐well plates where they were cultured for 2 months. From the original five dishes with outgrowing cells only one dish contained cells that could be propagated for further passages. From this dish, clone C with epithelial morphology was isolated and maintained for 23 passages with regular freezing of cell aliquots between passages. Clone C (passage 16) was used for single‐cell sorting (FACSAria; BD Biosciences) into 96‐well plates. After 24 hours, plates were checked for wells containing single cells. After 2 weeks, expanded colonies (>50 cells) were either subcultured into 24‐well plates, directly stained in the wells with anti‐keratin‐7 antibody (http://onlinelibrary.wiley.com/doi/10.1002/hep.29679/suppinfo), or used for further single‐cell sorting. In total, we determined the keratin‐7 phenotype in 1,043 single cell–derived subclones, comprising the three different keratin‐7 expression phenotypes (i.e., heterogeneous K7het, positive K7pos, negative K7neg). Parallel to this, we selected 47 subclones (f1 and f2 subclones) and propagated them separately for further clonal studies.

### RNA SEQUENCING

Total RNA was isolated using the Qiagen RNeasy Mini Kit and the Qiagen RNeasy FFPE kit for the cell clones and primary tumor samples, respectively. Samples from tumor areas with defined morphological features were collected by core biopsies taken from the formaldehyde‐fixed or paraformaldehyde‐fixed, paraffin‐embedded blocks (details are shown in http://onlinelibrary.wiley.com/doi/10.1002/hep.29679/suppinfo). For quality control of isolated RNA, concentrations and purity were measured with Nanodrop 1000 (Thermo Scientific), RNA integrity was analyzed by spectrophotometry, and different amplicon lengths were determined using quantitative RT‐PCR as described.^35^ For the cell clones, 500 ng of RNA was used for Illumina RNA sequencing (RNA‐seq) library construction using the Illumina TruSeq RNA Sample Preparation Kit v2 according to the manufacturer's instructions, except that one‐third of the recommended volumes was used in each step. For the primary tumor samples, ribosomal RNA was depleted from 1 μg of the total RNA for each sample using the Ribo‐Zero ribosomal RNA Removal Kit (Epicentre Biotechnologies). The ribosomal RNA–depleted RNA was piped into the Illumina TruSeq RNA Sample Preparation Kit v2 by resuspending in 6.5 μL of Elute, Prime, Fragment mix. The resulting RNA‐seq libraries were quantified using the Library Quantification Kit from KAPA Biosystems. The libraries were sequenced on an Illumina HiSeq 2000 at the EMBL GeneCore (Heidelberg, Germany). Reads were mapped using TopHat, assigned to genes using HTSeq‐count, and differentially expressed genes (DEGs) and per‐gene‐dispersion estimates were called using DESeq. For gene ontology analysis, we used GeneCodis^36^ and DAVID bioinformatics gene ontology annotation and the signaling pathway tool. For gene set enrichment analysis, we used the online molecular signature database (MSigDB)^37^, ^38^ with gene sets from the C2 database, which contains 1,892 curated gene sets that are collected from various sources including online pathway databases and knowledge of domain experts. *P* value thresholds were set to 0.01.

### METHYLATED DNA IMMUNOPRECIPITATION SEQUENCING

Genomic DNA was isolated from cells of subclones (K7het, K7pos, and K7neg) using the PureLink Genomic DNA Mini Kit (Life Technologies). Purified genomic DNA (4 μg in 120 μL) was transferred to a Covaris microTube and sonicated in a Covaris S2 sonicator using the following settings: time 7 minutes, duty cycle 10%, intensity 5, cycles per burst 200, temperature 4°C, and power mode frequency sweeping. The sheared DNA was precipitated with 1 volume of AMPure beads (Beckman Coulter) and 1 volume of 30% PEG_8000_, 1.25 M NaCl; washed 2 times with 75% ethanol; and resuspended in 41 μL of 10 mM Tris, pH 8.0, and 0.1 mM ethylene diamine tetraacetic acid. DNA ends were blunted and A‐tailed, and Illumina TruSeq adapters were ligated using an in‐house‐made version of Illumina's TruSeq DNA sample preparation kit. Methylated DNA immunoprecipitation was performed essentially as described (http://www.roadmapepigenomics.org/) except that a short oligonucleotide (AGATCGGAAGAGCGTC) was added to the denaturation reaction to prevent DNA fragments from annealing together by their adapter sequences. Libraries were amplified with Kapa HiFi DNA polymerase (Kapa Biosystems) and sequenced on an Illumina HiSeq 2000 (EMBL GeneCore). Reads were mapped to the human genome using Bowtie, and differentially methylated regions (DMRs) were identified with DiffReps (http://www.ncbi.nlm.nih.gov/pmc/articles/PMC3677880).

### BISULFITE PYROSEQUENCING

Quantification of CpG methylation at *KRT7* promoter was performed by pyrosequencing with predesigned assays (Human_*KRT7*_01_PM PyroMark CPG assay; Qiagen). Pyrosequencing was performed with Qiagen PyroMark. Detection and quantitative mutation analyses were performed by the inbuilt software (Pyrogram). Genomic DNA was extracted using the QIAamp DNA mini protocol (Qiagen) from either stable subclones (K7neg, K7pos) or fluorescence‐activated cell–sorted (FACS) keratin‐7‐positive cells or keratin‐7‐negative cells from K7het subclones.

### BROMODEOXYURIDINE ANALYSIS

Cells were grown on microscopic slides in six‐well plates and incubated in culture medium with 10 μM bromodeoxyuridine (BrdU) for 12 hours. Cells were fixed in BD Cytofix/Cytoperm Buffer, washed with BD Perm/Wash Buffer (BD Pharmingen), and stained with anti‐BrdU antibody (Abcam) and Alexa Fluor 488. Double staining was performed with anti‐keratin‐7 antibody (Dako) and secondary Alexa Fluor 594‐conjugated antibody.

### TUMOR XENOGRAFTS

For heterotopic xenograft transplantation, 6‐week‐old nonobese diabetic/severe combined immunodeficient mice were subcutaneously inoculated into their lateral flanks with 5 × 10^6^ cells and monitored daily until the tumors became palpable. We used two different subclones in early (10‐15 passages) and late (>20 passages) passages each, representing the three keratin‐7 phenotypes (K7het, K7pos, K7neg), in the experiment. At the end of the experiment the animals were killed, and tumors were excised, weighed, and fixed in formalin for immunohistochemical analysis. All animals were housed in individually ventilated cages in the Animal House Facility of the Biomedical Research Foundation of the Academy of Athens in pathogen‐free conditions, in full compliance with the recommendations of the Federation of Laboratory Animal Science Associations. The Greek Ministry of Agriculture (European Directive 86/609) approved all procedures concerning the protection of animals used for experimental purposes.

### STATISTICAL ANALYSIS

Values are expressed as mean ± SD. Quantitative RT‐PCR validation is represented in log_2_ scale (Student *t* test: ^*^ *P* < 0.05, ^**^ *P* < 0.01, ^***^ *P* < 0.0001; n = 3‐4 f1 clones). DiffReps were used to associate DMRs with DEGs. *P* values were calculated on the hypergeometric distribution for DMRs. Cumulative tumor weights between different clonal phenotypes were calculated by one‐way analysis of variance with uncorrected Fisher's least significant difference test (^**^ *P* < 0.01, ^****^ *P* < 0.0001). DEGs were identified using the data analysis package DESeq.^39^

## Results

### CHARACTERIZATION OF EPITHELIAL AND MESENCHYMAL PHENOTYPES IN SARCOMATOID CHOLANGIOCARCINOMA

**Figure 1:**
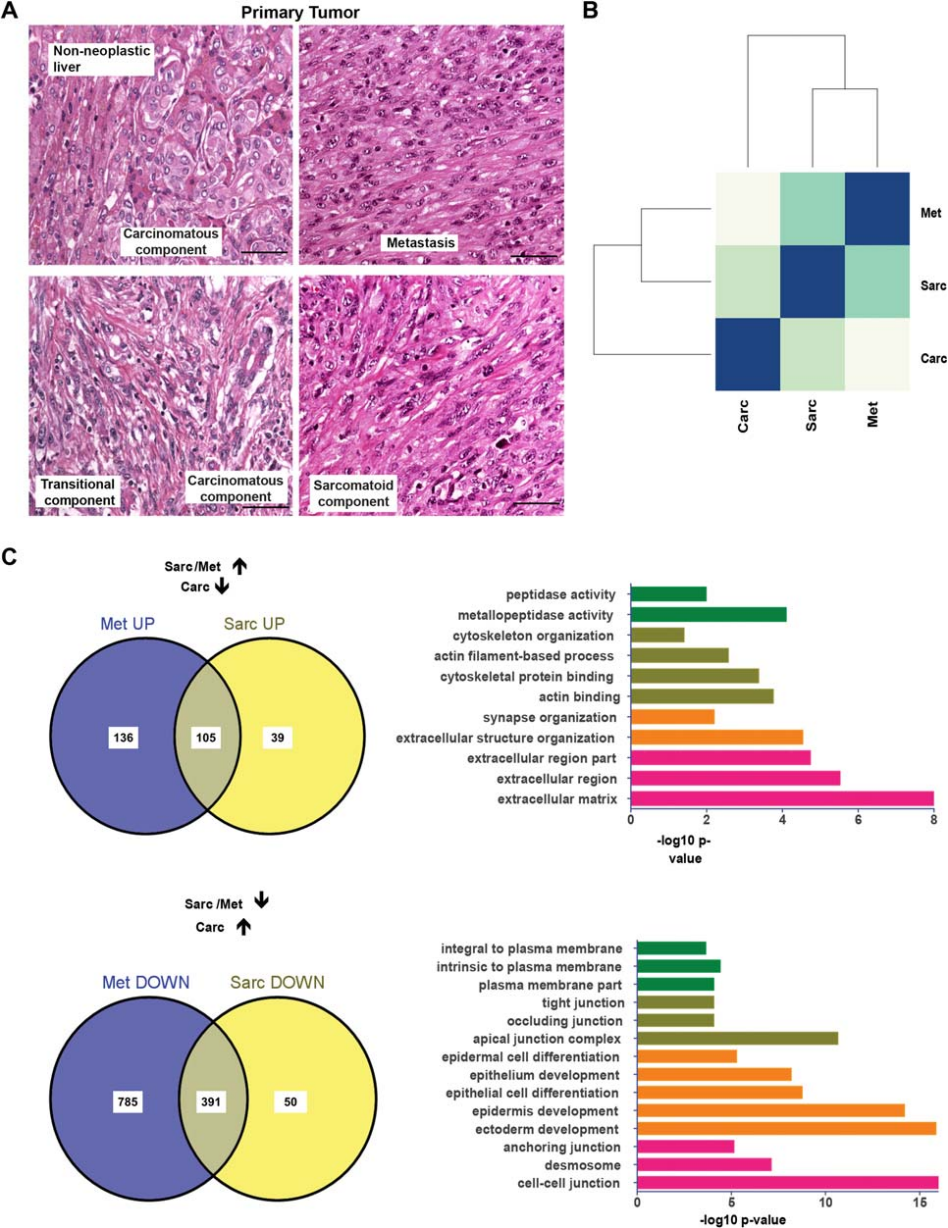
Characterization of sarcomatoid cholangiocarcinoma. (A) Hematoxylin and eosin–stained sections in primary tumor and metastasis displaying non‐neoplastic, carcinomatous, transitional, sarcomatoid, and metastatic components. Scale bar, 50 μm. (B) Heatmap and dendogram of sample‐to‐sample Euclidean distances of transcriptome profiles between different tumor components. (C) Venn diagram (upper panel) of unique and common up‐regulated genes (up arrow) from comparing the sarcomatoid component and lymph node metastasis to the carcinomatous component. Enriched gene ontology categories from the overlapping up‐regulated genes (n = 105, right). Venn diagram (lower panel) of unique and common down‐regulated genes (down arrow) by comparing the sarcomatoid component and lymph node metastasis to the carcinomatous component. Enriched gene ontology categories from the overlapping down‐regulated genes (n = 391, right). Abbreviations: Carc, carcinomatous; Met, metastatic; Sarc, sarcomatoid.

We analyzed a surgically resected intrahepatic cholangiocarcinoma with characteristics of sarcomatoid trans‐differentiation (details of the clinical features and pathological features are described in http://onlinelibrary.wiley.com/doi/10.1002/hep.29679/suppinfo and http://onlinelibrary.wiley.com/doi/10.1002/hep.29679/suppinfo). The carcinoma showed heterogeneous histopathological attributes with regionally distinct invasive tumor components (satellite nodules) and lymph node metastasis (Fig. 1A). Tumor components with tubular structures were classified as the carcinomatous component, while satellite nodules with predominant spindle cells, which were also present in the lymph node metastasis, were classified as the sarcomatoid component (Fig. 1A). Furthermore, the carcinomatous part contained areas with mixed cells showing sarcomatoid and carcinomatous features (transitional component) (Fig. 1A). Such transitional areas have been suggested to be the result of the metaplastic transformation of epithelial cells to mesenchyme‐like cells.^28^, ^40^, ^41^

To further characterize the heterogeneity in different tumor regions, we generated transcriptional profiles by RNA‐seq of each component. Comparison of the sequencing profiles showed more similarities in gene expression between sarcomatoid and metastatic than between carcinomatous and sarcomatoid or metastatic components, supporting the notion that metastasis has emerged from the sarcomatoid component (Fig. 1B). Furthermore, comparison between the carcinomatous and sarcomatoid tumor components and the carcinomatous and metastatic tumor components identified 585 and 1,418 DEGs, respectively (log_2_ fold change ≥2.0, *P*_adjusted_ < 0.01), characteristic of the invasive and metastatic tumor components.^32^, ^42^ Gene ontology analysis with functional annotation clustering of DEGs (Fig. 1B,C) in components with sarcomatoid differentiation (and/or metastatic) showed down‐regulation of cell–cell junction–related genes (DSP, JUP, PVRL4, GJB3, GJB4, GJB6, TJP3, CGN) and epithelial differentiation‐related genes (CDH1, CDH3, CRB3, OCLN, DDR1, EPCAM, CLDN3, FGFR2b), while up‐regulated genes were associated with mesenchymal differentiation (MMP2, MMP11, CDH2, VIM, MCAM, SERPINE1), invasion/migration‐related genes (PDPN, VEGFC, TLN2), EMT‐related genes (TWIST1, TGFBR1, ZEB1, ZEB2), as well as stem/self‐renewal genes (NCAM1, PROCR, ANPEP) (http://onlinelibrary.wiley.com/doi/10.1002/hep.29679/suppinfo). Furthermore, immunohistochemistry verified the expression changes of DEGs between the carcinomatous and sarcomatoid tumor components (not shown). However, we detected no apparent contribution of hepatic stem cells/progenitor cells to intratumor heterogeneity (http://onlinelibrary.wiley.com/doi/10.1002/hep.29679/suppinfo).

### INTRATUMOR HETEROGENEITY ARISES FROM A CELL SUBPOPULATION UNDERGOING STOCHASTIC SWITCHING OF DIFFERENTIATION PHENOTYPES

**Figure 2:**
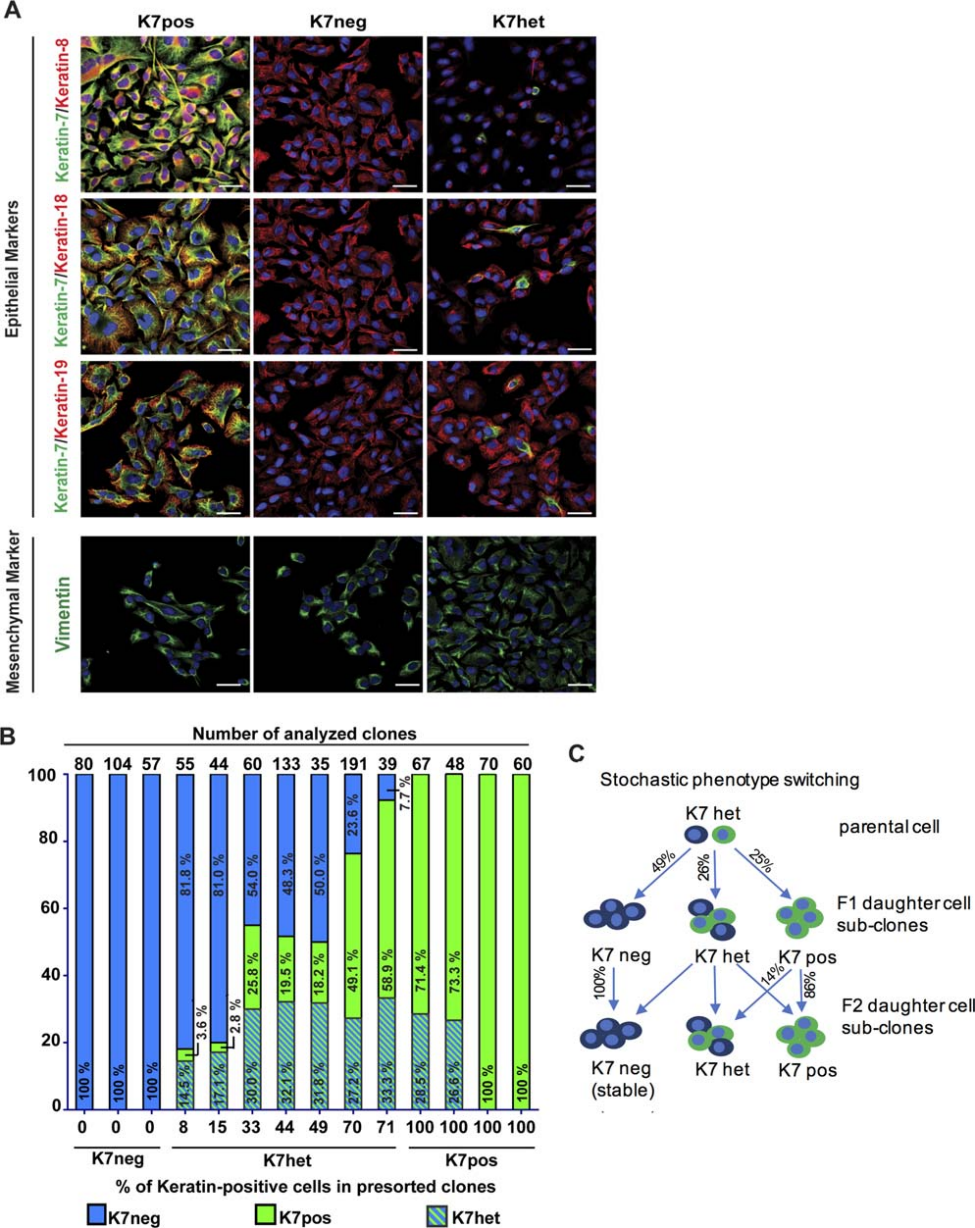
Characterization of K7pos, K7neg, and K7het clonal cell populations. (A) Immunofluorescence microscopy of different clonal phenotypes. K7pos, K7neg, and K7het cells were analyzed by with triple‐label immunofluorescence staining (keratin‐7 in combination with keratin‐8, keratin‐18, and keratin‐19 and 4′,6‐diamidino‐2‐phenylindole as nuclear stain) or double‐staining (4′,6‐diamidino‐2‐phenylindole in combination with vimentin). Scale bar, 20 μm. (B) Maintenance of keratin‐7 phenotypes in daughter cell subclones. Proportions of keratin‐7 phenotypes (*y* axis) in cells derived from single‐cell sorted parental cell clones with different K7 phenotypes (K7neg, K7het, K7pos) with a variable percentage of keratin‐7‐positive cells (numbers at *x* axis). Each column represents results from a separate experiment for which the total number of analyzed clones is indicated at the top of the column. (C) Schematic demonstration of clonal phenotypes (K7neg, K7pos, K7het) in daughter cell subclones derived from single cells of a K7het parental clone showing stochastic phenotype switching. Average percentages of phenotype propagation to daughter cell subclones are derived from 1,043 subclonal phenotype analyses shown in (B).

To characterize the mechanisms that govern intratumor phenotype heterogeneity, we investigated a primary cell culture (hereafter referred to as sarcomatoid cholangiocarcinoma parental cell culture) established from the surgically resected primary liver tumor. FACS analysis of early‐passage cell culture (clone C, passage 10, which was further used for the experiments reported) showed epithelial origin and did not reveal contamination with stromal or inflammatory cells (http://onlinelibrary.wiley.com/doi/10.1002/hep.29679/suppinfo). To demonstrate that the cell culture established is derived from the tumor, we performed targeted mutation hotspot analyses of 46 cancer‐related genes. We detected seven variants above the call threshold including a BRAF V600E mutation, which has been described in cholangiocarcinoma with poor prognosis.^43^ Importantly, the same seven variants were found in the original human tumor sample and the derived cell culture subclones (http://onlinelibrary.wiley.com/doi/10.1002/hep.29679/suppinfo). Keratin‐7 expression was used as a marker to distinguish between epithelial and mesenchymal differentiation in sarcomatoid parental cell culture and in single cell–derived subclones (Fig. 2A). We categorized the subclonal types into keratin‐7 positive (K7pos), keratin‐7 negative (K7neg), and keratin‐7 heterogeneous (K7het) based on keratin‐7 expression in single cell–derived subclones. Immunofluorescence profiling revealed the concurrent expression of epithelial cell–specific markers keratin‐8, keratin‐18, and keratin‐19, as well as expression of the mesenchymal marker vimentin (Fig. 2A), indicating the metastable epithelial and mesenchymal nature of the cell subclones.^19^, ^44^, ^45^ Interestingly, we observed that in K7neg subclones E‐cadherin expression was down‐regulated,^46^ whereas fibronectin expression was up‐regulated, suggesting that K7neg subclones were residing in a more mesenchymal state^47^ (http://onlinelibrary.wiley.com/doi/10.1002/hep.29679/suppinfo).

Furthermore, we analyzed the stability of the keratin‐7 phenotype in a total of 1,043 single cell–derived daughter subclones (Fig 2B). K7het subclones yielded daughter subclones corresponding to all three keratin‐7 subclonal types, that is, K7neg, K7pos, and K7het (Fig. 2B,C). On average, approximately 26% of all K7het daughter subclones maintained the noncommitted K7het phenotype with the ability of stochastic phenotype switching between K7pos and K7neg phenotypes. Interestingly, 14% of K7pos subclones (which were originally derived from K7het parental clones) gave rise to K7het daughter subclones, which demonstrates the reversibility of phenotype switching (Fig. 2B,C). In contrast, all K7neg subclones stably propagated their K7neg phenotypes to their daughter subclones, which suggests that K7neg subclones have acquired a stable phenotype (Fig. 2B,C).

### CLONAL KERATIN‐7 PHENOTYPES CONSTITUTE DISTINCT TRANSCRIPTIONAL PROFILES

**Figure 3:**
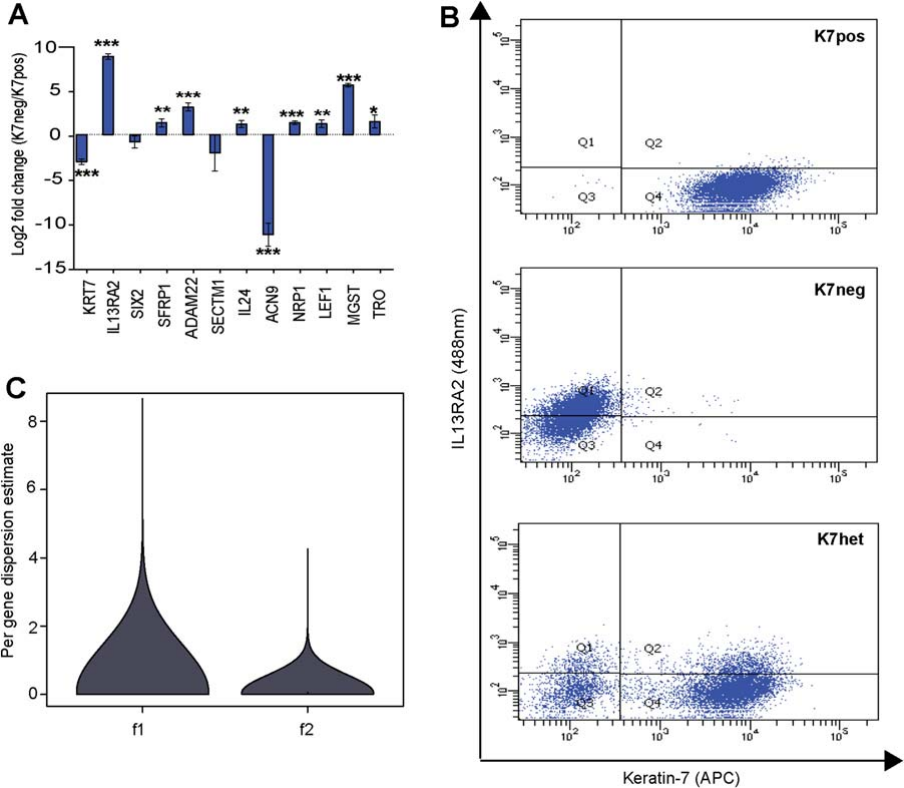
Phenotypic characterization of K7neg, K7pos, and K7het subclonal cell populations. (A) Quantitative RT‐PCR validation of 12 DEGs identified by transcriptome sequencing. Differential expression is represented in log2 scale (Student *t* test, ^*^ *P* < 0.05, ^**^ *P* < 0.01, ^***^ *P* < 0.0001; n = 3‐4 f1 clones). (B) FACS analysis of K7pos, K7neg, and K7het subclones coimmunostained with anti‐keratin‐7 and anti‐IL13RA2 antibodies. Ten K7het and four different K7pos and K7neg cell subclones were individually analyzed, and results are displayed as scatter plots in their respective panels. (C) Violin plot representation of the per‐gene variance distribution based on transcriptome sequencing of K7pos and K7neg f1 cell subclones compared to K7pos and K7neg f2 cell subclones. The f2 K7pos and K7neg subclones were derived from a single f1 cell each (n = 5 for each clonal type, Student *t* test, *P* = 1.6 × 10^–15^).

Transcriptional profiling (RNA‐seq) of K7pos and K7neg subclones identified 78 DEGs, of which 44 were significantly overexpressed and 34 underexpressed in K7neg clones compared to K7pos clones (fold change ≥2.0, *P* < 0.05). A literature‐based query characterized DEGs as cancer‐specific and prognostic markers, genes associated with cancer proliferation, migration, and invasion. Importantly, genes involved in liver carcinogenesis and malignant transformation of hepatic progenitor cells were also among the identified DEGs (http://onlinelibrary.wiley.com/doi/10.1002/hep.29679/suppinfo). The expression pattern of a set of randomly selected DEGs (n = 12) was verified by quantitative RT‐PCR (Fig. 3A). The fold changes in gene expression observed by quantitative RT‐PCR significantly correlated with the fold changes in gene expression observed by RNA‐seq (*r* = 0.886, *P* < 0.001).

To determine if the alterations in mRNA levels correlated with changes in protein levels at the single‐cell level, we selected the *IL13RA2* gene, which negatively correlated with *KRT7* mRNA expression, and performed double‐stained FACS analysis with anti‐keratin‐7 and anti‐IL13RA2 antibodies. In stable K7pos subclones nearly 100% of the cells were IL13RA2‐negative, whereas K7neg subclones contained populations of both IL13RA2‐positive and IL13RA2‐negative cells (Fig. 3B). Interestingly, K7het subclones contained a fraction of keratin‐7/IL13RA2‐positive cells, a characteristic that was not observed in either of the stable cell phenotypes (K7pos or K7neg) (Fig. 3B). These results demonstrate that K7het subclones are not simply a mixture of K7pos and K7neg cells (Fig. 3B, lower panel, quadrant Q2) but that they contain a phenotypically unstable subpopulation of cells which may generate daughter cells of different phenotypes.

To further characterize the transcriptional stability of the K7pos and K7neg subclones, we compared the per‐gene biological variance between replicates of K7pos and K7neg subclones derived from single cells from the primary sarcomatoid parental cell culture (f1 clones) and daughter subclones derived from a single f1 K7pos or K7neg subclone (f2 clones) (Fig. 3C). Interestingly, we discovered that the per‐gene variances between different f1 subclones were much higher compared to the average per‐gene variances between their daughter f2 subclones. Thus, by probing the gene expression variance between different clonal generations we observed that not only was *KRT7* gene expression stabilized in the K7pos and K7neg clones but there also exists a general mechanism leading to global transcriptome stabilization during the transition from an unstable keratin‐7 expressing cell (K7het) to a more stable keratin‐7 phenotype (K7pos and K7 neg) (Fig. 3C). Furthermore, it appears that sequential cell divisions reduce the overall noise and globally stabilize the transcriptional program of inherently phenotypically unstable cells.

### DIFFERENTIAL *KRT7* mRNA AND PROTEIN EXPRESSION IS MEDIATED BY DNA METHYLATION IN K7pos AND K7neg BUT NOT K7het CELL CLONES

**Figure 4:**
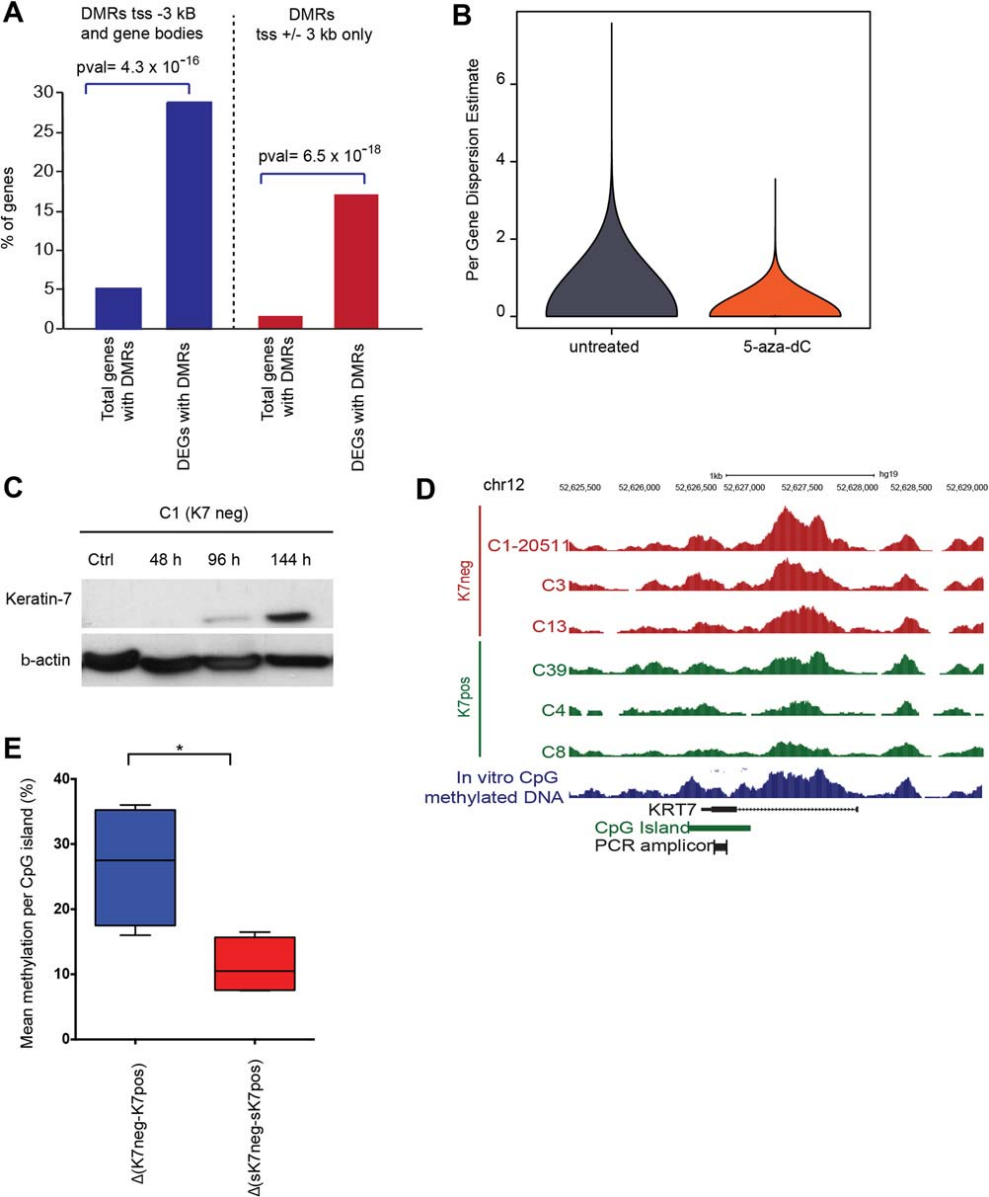
Difference in DNA methylation in stable cell subclones and subclones with stochastic phenotype switching. (A) DMRs preferentially associated with promoters (right) and promoters and gene bodies (left) of DEGs. (*P* values, hypergeometric test). (B) Violin plot representing the distribution of per‐gene variance in 5‐aza‐dC‐treated K7pos and K7neg subclones (n = 4 for each clonal type) compared to corresponding nontreated subclones (*t* test, *P* = 2.2 × 10^–16^). (C) Western blot of reexpressed keratin‐7 in K7neg cells treated with 5‐aza‐dC at indicated time points. β‐Actin was used as reference. (D) A genome browser screenshot of the *KRT7* promoter showing MeDIP‐seq data from six independent subclones, where K7neg subclones (C12205, C3, and C13; red) had increased 5‐methylcytosine levels compared to K7pos subclones (C39, C4, and C8; green). As a reference, MeDIP‐seq of *in vitro* fully CpG methylated genomic DNA is shown in blue. (E) Relative methylation differences between phenotypically stable K7pos and K7neg subclones, Δ(K7neg‐K7pos), are significantly higher compared to methylation levels between keratin‐7‐positive and keratin‐7‐negative cells, Δ (sK7 neg‐sK7pos) cell‐sorted from unstable K7het subclones (n = 4, Student *t* test, ^*^ *P* < 0.05). Abbreviation: tss, transcription start site.

Previous studies have implicated variability in DNA methylation patterns in the context of stochastic gene expression in cancer cells.^48^ To test the role of DNA methylation in global transcriptional stability, we used methylated DNA immunoprecipitation sequencing (MeDIP‐seq) of different keratin‐7 clonal types and identified 3,344 DMRs (false discovery rate <0.05). DEGs demonstrated a much higher association with DMRs than nondifferentially expressed genes, indicating that in cells with a stable phenotype the observed changes in gene expression were associated with changes in DNA methylation (Fig. 4A). Because stabilization of *KRT7* expression correlated with global transcriptional stabilization, we investigated whether gene expression in the different clonal phenotypes correlates with DNA methylated regions in the genome.^49^ RNA‐seq analysis of K7pos, K7neg, and K7het subclones (f1) treated with the DNA methyltransferase inhibitor 5‐aza‐2′‐deoxycytidine (5‐aza‐dC) revealed a remarkable decrease in variance; that is, the similarity between the 5‐aza‐dC‐treated K7pos and the K7neg subclones was greater than that observed between untreated corresponding clones (Fig. 4B). Treatment of K7neg subclones with 5‐aza‐dC caused keratin‐7 protein reexpression and increased the percentage of keratin‐7‐positive cells in a time‐dependent and dose‐dependent manner (Fig. 4C). However, reactivation of keratin‐7 expression occurred only in a subfraction of cells (http://onlinelibrary.wiley.com/doi/10.1002/hep.29679/suppinfo), suggesting cell‐to‐cell variability in responses to 5‐aza‐dC treatment. We verified these results with MeDIP‐seq of three independently derived K7pos and K7neg f1 subclones, which showed a significant increase in DNA methylation at the CpG islands associated within the *KRT7* promoter in all K7neg clones compared to K7pos cells (Fig. 4D). Further, we performed pyrosequencing of bisulfite‐treated DNA of the FACS‐sorted keratin‐7‐positive and keratin‐7‐negative cells from phenotypically unstable K7het subclones. Surprisingly, comparison of DNA methylation analysis results from phenotypically stable K7neg cells showed that methylation of the *KRT7* promoter was much more pronounced than in sorted keratin‐7‐negative cells of phenotypically unstable K7het subclones (Fig. 4E). This implies that mechanisms not related to DNA methylation are involved in regulating keratin‐7 expression in cells undergoing stochastic phenotype switching.

### PHENOTYPE SWITCHING OCCURRED DURING MITOSIS

**Figure 5:**
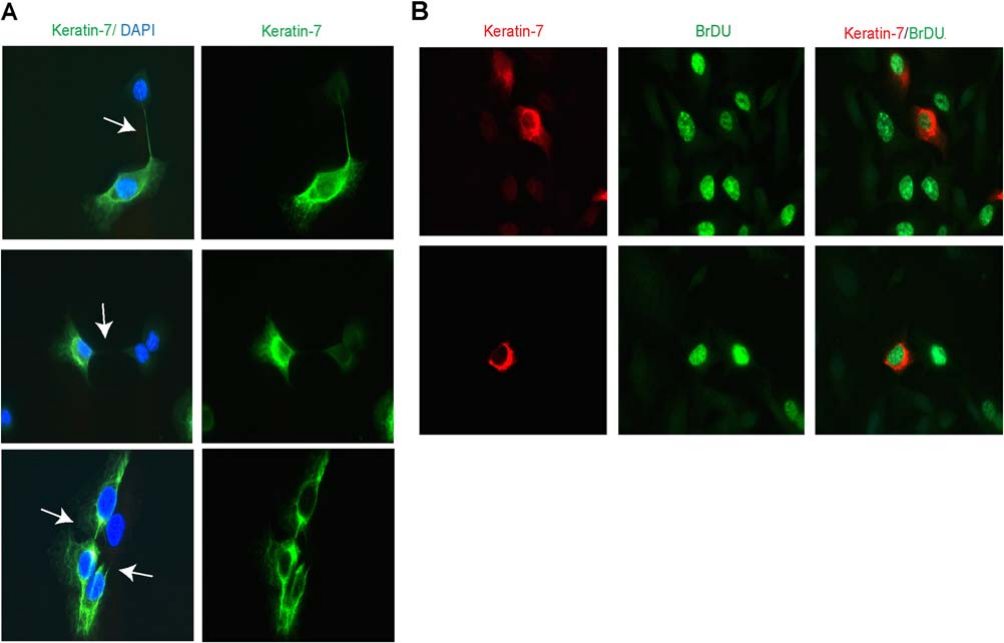
Stochastic phenotype switching and asymmetric cell division in K7het clones. (A) Immunofluorescence microscopy of keratin‐7 expression in daughter cells immediately after cell division. White arrows point to connections between daughter cells. Lower panel: Uneven expression of keratin‐7 in daughter cells (right arrow) and daughter cells with same keratin‐7 phenotypes (left arrow). Magnification: ×630. (B) Actively dividing K7het subclonal cell incubated with BrdU and double‐stained with anti‐keratin‐7 and anti‐BrdU. Abbreviation: DAPI, 4′,6‐diamidino‐2‐phenylindole.

To gain further insight into the stochastic modalities of heterogeneous keratin‐7 expression in single cells, we investigated keratin‐7 expression in daughter cells after mitosis. In several independent K7het clones, we observed different keratin‐7 staining of daughter cells during cytokinesis or immediately after the cell division phase (Fig. 5A). Such changes in keratin‐7 phenotypes were never observed in dividing K7pos and K7neg cells (not shown). To further characterize the asymmetrical partitioning of keratin‐7 expression that occurs between daughter cells, we preincubated cells with BrdU. Double staining for BrdU and keratin‐7 confirmed unequal partitioning between daughter cells (Fig. 5B).

### LOSS OF KERATIN‐7 EXPRESSION RESULTS IN INCREASED TUMORIGENICITY *IN VIVO*

**Figure 6:**
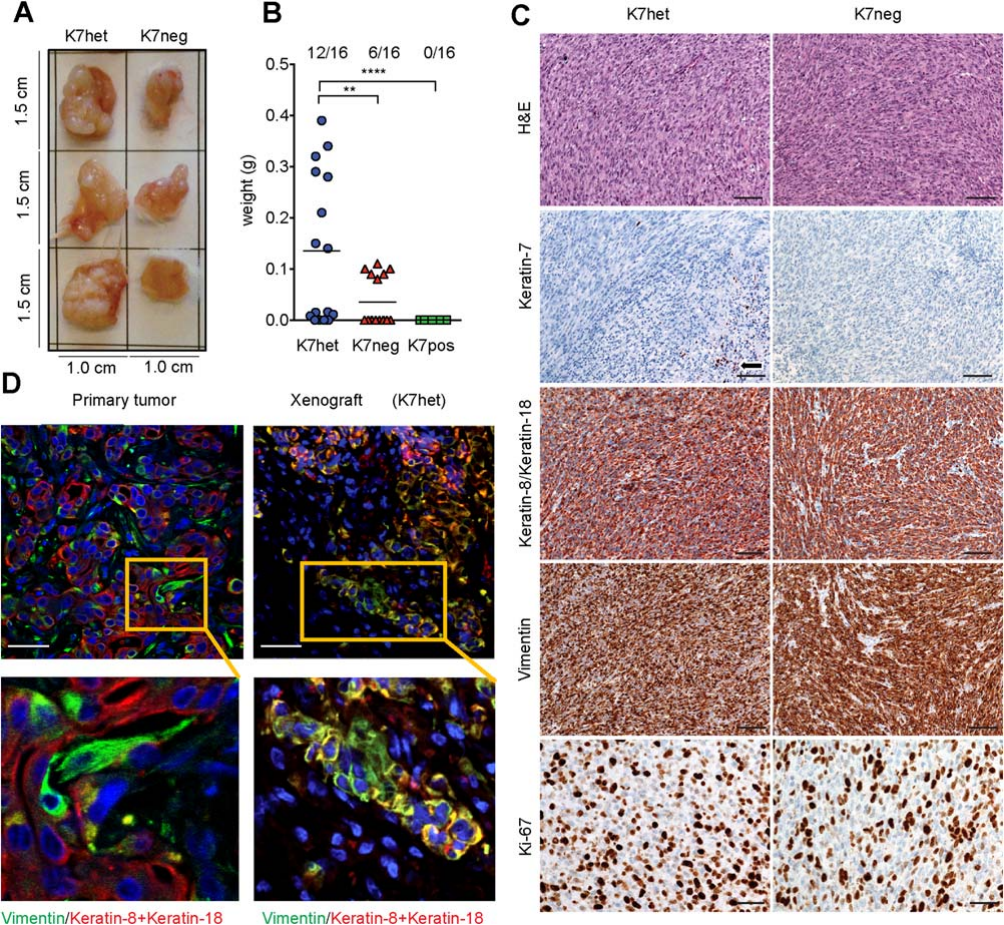
Keratin‐7‐negative subclones show increased tumorigenic potential in xenografts. (A) Representative pictures of established tumor xenografts from K7neg and K7het subclones after 33 and 22 days, respectively. (B) Mean tumor weights developed from the different clonal phenotype. Two independent subclones per clonal phenotype were injected into multiple mice (tumor weights are shown as mean ± SD; total number of developed tumors/total number of injection sites is shown at the top; one‐way analysis of variance with uncorrected Fisher's least significant difference test (^**^ *P* < 0.01, ^****^ *P* < 0.0001). (C) Hematoxylin and eosin and immunohistochemical staining of xenografted tumors. Tumors were derived from K7het and K7neg subclones and stained with anti‐keratin‐7 antibodies (arrow indicates residual keratin‐7‐positive cells associated with necrotic and apoptotic tumor cells), anti‐keratin‐8+18 antibodies, antivimentin antibodies, and the Ki67 antibody. Note that xenografts developed from K7het subclones showed a keratin‐7‐negative phenotype. Scale bar for hematoxylin and eosin, keratin‐7, keratin‐8/keratin‐18, vimentin images, 50 μm. Scale bar for Ki‐67 images, 100 μm. (D) Triple immunofluorescence staining of the primary tumor and corresponding xenografts with anti‐keratin‐8 (red) and antivimentin (green) antibodies and nuclear 4,6‐diamidino‐2‐phenylindole dye (blue). Scale bar, 20 μm; insets in upper panels indicate areas shown in higher magnification in lower panels. Abbreviation: H&E, hematoxylin and eosin.

To investigate the biological relevance of the different keratin‐7 phenotypes in cancer development, we evaluated their tumorigenic potential in a nonobese diabetic/severe combined immunodeficient mouse tumor xenograft model. The average time for the appearance of tumors after subcutaneous implantation of cells was 29 ± 6 days. Only K7het and K7neg subclones established tumors, whereas none of the K7pos subclones led to visible tumor growth at any of the injected sites (n = 8 per clonal type) (Fig. 6A). We also observed variability between clonal types and weights of the tumors after resection (Fig. 6B). Histological analysis of the developed tumors revealed mostly spindle cell morphology and an invasive growth pattern, which resembles the invasive sarcomatoid component of the primary patient tumor and the metastasis (Fig. 6C). Importantly, immunohistochemical analysis showed that all K7het‐derived tumors were composed of keratin‐7‐negative cells (Fig. 6C), except for a few scattered keratin‐7‐positive cells, most of which showed features of apoptosis and were located in necrotic tumor parts (Fig. 6C). This was a surprising result because the injected K7het subclones contained >60% keratin‐7‐positive cells (clone CCC C: 78% keratin‐7‐positive cells; CCC C1: 63% keratin‐7‐positive cells). Both K7het and K7neg‐derived tumors concomitantly expressed keratin‐8, vimentin, and Ki‐67 markers. In addition, in the xenograft environment, as in the primary patient tumor, we observed that some tumor cells lost keratin‐8 expression, while maintaining expression of vimentin (Fig. 6D).

## Discussion

Our study addressed the increasingly important issue of intratumor heterogeneity and characterized a mechanism generating heterogeneity, which is not based on genetic clonal evolution, fixed hierarchical organization, tumor stem cells, or influences of the tumor microenvironment. Rather, we propose a molecular mechanism where stochastic phenotypic switches occurring during mitosis lead to the establishment of unique transcriptional programs involved in the functional diversification of cancer cell populations. Importantly, in the investigated subclones of the primary tumor cell culture epigenetic mechanisms, such as DNA methylation, stabilized stochastically generated phenotypes, rather than generating tumor heterogeneity.

By monitoring the fate of single cells derived from a primary culture of a human liver sarcomatoid cholangiocarcinoma, we determined the phenotypic and molecular history of 1,043 single cell–derived subclones. We identified distinct self‐propagating subclones characterized either as essentially stable (K7pos or K7neg) or as unstable clones (K7het). K7het clones can stochastically either propagate to the stable phenotypes (K7pos or K7neg) or transfer their phenotypically unstable cell nature (K7het) to daughter generations. The morphology of each clonal phenotype appears stable in subsequent rounds of single‐cell sorting, including the persistent unstable nature (i.e., stochastic phenotype switching) of K7het subclones. In K7het subclones, intraclonal heterogeneity stochastically occurs in individual cells following mitosis, and it is associated with adjustments of the cell's transcriptional program. Mechanistically, we showed that hypermethylation of the *KRT7* promoter is involved in silencing *KRT7* expression in stable K7neg subclones. Interestingly, we found significantly lower methylation of the *KRT7* promoter in the keratin‐7‐negative sorted cell fraction derived from the (phenotypically unstable) K7het subclones than in the stable K7neg subclones, suggesting the existence of mechanisms other than DNA methylation in repression of keratin‐7 expression in K7het cells showing stochastic phenotype switching. We hypothesize that the uneven distribution of rate‐limiting, phenotype‐regulating factors to daughter cells^50^ leading to the reestablishment of genome‐wide reprogramming mechanisms might be responsible for these effects.

Although previous studies have suggested that epigenetic mechanisms could continuously generate sufficient diversity in clonal cell populations, our results indicate that the repressive DNA methylation mark on stable keratin‐7‐negative cells is placed after shutting off transcription, indicating that epigenetic mechanisms do not generate phenotypic variability but instead maintain the repressed state after cell division. Consistently, there has been recent recognition of the similarities in epigenetic mechanisms between cellular reprogramming and transformation of normal cells, albeit that different epigenetic mechanisms may operate in these cases.^23^, ^51^

Our RNA‐seq data analysis revealed that variance in gene expression distinguished true biological variability between different tumor cells. By taking into account the coefficients of gene expression variation over biological replicates, we further implicated the role of cellular plasticity in relation to the stability of genetic networks and heterogeneity of neoplastic phenotypes.^2^, ^8^ The transcriptional profiles of propagated subclones demonstrated a reduction of global variance in gene expression with increasing number of clonal generations derived from either heterogeneous or stable phenotypes of parental subclones. These results suggest that progressive clonal history might be reflected in the dynamic transcriptional profiles supporting the transition from an unstable toward a more stable phenotypic state, thus generating distinct stable cell populations contributing to intratumor heterogeneity. Furthermore, 5‐aza‐dC treatments greatly reduced the per‐gene variance between all clonal types, supporting the role of global changes in methylation patterns in establishing variations in gene expression profiles.

An important feature of the primary tumor cell culture and the derived subclones is the preservation of the genetic variants of the original tumor. The simultaneous expression of epithelial (e.g., keratin‐8, keratin‐18, keratin‐19) and mesenchymal (e.g., vimentin, fibronectin) markers together with the comparative transcriptomes suggested that the cells were maintained in an incomplete EMT‐like state and that acquisition of mesenchymal features in K7neg clones occurred stochastically in the absence of any external inducing factor. The possible role of EMT in tumor propagation was substantiated by the expression of a variety of markers described to be involved in EMT, such as Zeb1 and CD146/MCAM. The expression of EMT markers was similar in the primary tumor, cultured cell subclones (CD146/MCAM), and xenografts (Zeb1) (http://onlinelibrary.wiley.com/doi/10.1002/hep.29679/suppinfo). However, on the single‐cell basis no direct correlation between expression of EMT markers and keratin‐7 expression was found (not shown), indicating that EMT and stochastic phenotype switching are related but regulated by different mechanisms.

Based on the expression of keratin‐7 as an epithelial differentiation marker protein, we observed that only cells with a keratin‐7‐negative phenotype were capable of producing tumors in xenografts. Furthermore, all established tumor xenografts had an undifferentiated sarcomatoid morphology that morphologically resembled the invasive and metastatic components of the patient tumor. In this context, it was of particular interest that xenografts of K7het subclones, which contained >60% keratin‐7‐positive cells, led to tumors which were essentially negative for keratin‐7. This indicates that loss of keratin‐7 expression correlates with *in vivo* tumorigenicity. Moreover, there was a greater take rate of xenografts after injection of K7het subclones (12 tumors developed at 16 injection sites) compared to K7neg subclones (6 tumors developed at 16 injection sites) (Fig. 6), indicating that the ability of stochastic phenotype switching (as it is present in the K7het subclones) could be a feature required for better adaptation to the environment of a xenograft, thus resulting in greater tumor generation efficiency compared to cells with a stable keratin‐7‐negative phenotype. The essential absence of keratin‐7‐positive cells in xenografts developed from K7het subclones could result from a survival disadvantage of keratin‐7‐positive cells compared to keratin‐7‐negative cells or indicate that keratin‐7‐positive cells switched to a keratin‐7‐negative phenotype (Fig. 2C). The observation that K7het‐injected mice developed more and larger tumors than K7neg‐injected mice favors the latter hypothesis.

Our observations on stochastic phenotype switching highlight the importance of aberrant trans‐differentiation processes in the occurrence of intratumor heterogeneity. Importantly, these processes are cell‐autonomous because they occur in isolated single cells that can stochastically switch from one phenotype to the other by altering their transcriptional profile. Thus, the inherently unstable transcriptional status of K7het cells is not induced and/or maintained by the tumor environment or other external factors but rather has been endogenously generated early in tumor development through yet unknown mechanisms. Therefore, stochastic phenotype switching is a mechanism contributing to intratumor heterogeneity in human liver cancer, which sheds new light on the biology and medical relevance of heterogeneous tumors. The pervasive differentiation phenotype instability of cancer cells may further open opportunities for induced differentiation and/or homogenization therapies of poorly differentiated carcinomas in the process described as epigenome “reshuffling” of cancer cells.^52^

Author names in bold designate shared co‐first authorship.

## Supporting information

Additional Supporting Information may be found at http://onlinelibrary.wiley.com/doi/10.1002/hep.29679/suppinfo.

Supporting Information 1
