# Supplementary material for: Stochastic phenotype switching leads to intratumor heterogeneity in human liver cancer
Source: Hepatology. 2018 Feb 1;68(3):933–48. doi: 10.1002/hep.29679 (PMC6175233; doi:10.1002/hep.29679)
Supplement: Supplementary file 1 — Supporting Information 1 [file HEP-68-933-s001.pdf]

Supporting Information

Supporting Text

Supporting Material and Methods

Immunohistochemistry and immunofluorescence microscopy

Immunohistochemistry was performed on formalin-fixed and paraffin-embedded (FFPE) tissue sections (2 µm). Deparaffinized and hydrated sections were exposed to heat-induced antigen retrieval in citrate buffer (0.1%, pH 6.0) for 5 minutes in a pressure cooker at 125°C and gradually cooled in the cooker for 10 minutes. Endogenous peroxidase activity was blocked with hydrogen peroxide (1.0%) for 10 min at RT. Sections were incubated with primary antibodies overnight at 4°C. After washing in PBS, the slides were incubated with biotinylated secondary antibodies (a summary of all antibodies used is shown in **Supporting Table S1**). After washing in PBS, bound secondary antibodies were detected and developed with the ABC system (Dako) with AEC or DAB (0.06% 3,3'-diaminobenzidine with PBS, 0.003% H_2O_2) as chromogenic signal detectors. Slides were counterstained with Hematoxylin and mounted in water-soluble Aquafix solution. As a negative control, primary antibodies were replaced by PBS.

For indirect immunofluorescence staining, cells were either grown on coverslips and stained or directly stained in 96-well plates. Cells were fixed in ice-cold methanol for 5 minutes and acetone for 30 seconds. Cells were washed in PBS and incubated with primary antibodies for 30 minutes to 1 hour at room temperature. Secondary antibodies used were either Alexa Fluor 488 or 594-conjugated antibodies (Invitrogen). Samples were mounted in a Dako Mounting medium with DAPI (Invitrogen). Immunofluorescence signals were detected by Zeiss AxioPhot fluorescence microscope or by Zeiss LSM 510 META scanning laser confocal microscope.

FACS analysis

For characterization of cellular composition of Clone C cultures, cells of passage 10 culture were treated with trypsin/EDTA (Gibco/Thermo Fisher), washed and re-suspended in PBS. Cells ($5x10^5$) were then labeled either with unconjugated antibodies directed to CD68, CD3, Ve-Cadherin or Ep-CAM in combination with FITC-secondary antibody or APC-conjugated antibody directed to CD19 for 20 min at room temperature. Fluorescence antibody-stained cells were re-suspended in washing buffer and analyzed using CytoFlex S flow cytometer (Beckman Coulter).

For FACS analysis (FACSCalibur; BD Biosciences) and FACS sorting (FACS Aria; BD Biosciences), single cell suspensions were fixed by Cytofix-Cytoperm kit (BD Biosciences) for 15 minutes at 4°C, followed by staining with anti-keratin-7 antibody (DAKO) and Alexa Fluor 488–conjugated secondary antibodies (Invitrogen). Double staining was performed with anti-keratin-7 antibody and Alexa Fluor 594-conjugated secondary antibodies (Invitrogen), and fluorescein-conjugated human IL-13 R alpha 2 antibody (R&D Systems).

Ion Torrent Amplicon Sequencing

Ion Torrent sequencing was performed on DNA from cryopreserved primary tumor samples, non-neoplastic liver and cellular sub-clones. DNA was extracted with the QIAamp® DNA Mini kit (Qiagen). Ten nanograms of DNA were used for highly multiplexed PCR of 190 amplicons covering 739 mutations in 46 cancer-related genes (Ion AmpliSeq Cancer Panel Primer Pool, Life Technologies). Subsequent processing was performed according to the manufacturer's protocols for library construction and emulsion PCR using the Ion OneTouch system. Sequencing was done on the Ion Torrent Personal Genome Machine (Life Technologies). Data analysis, including base calling, alignment to the hg19 human reference genome and variant calling, was done using Ion Torrent Suite Software (Life Technologies).

Western Blots

Whole cell protein extracts from each clonal cell type were separated by electrophoresis on 12% SDS gels. Proteins were transferred onto nitrocellulose pore membranes using

a semi-dry iBlot transfer device (Life Technologies). Binding of the monoclonal keratin-7 antibody (Clone OV-TL 12/30, Dako) and anti-β-actin antibody (Clone AC-74, Sigma-Aldrich) as a loading control were detected with a horseradish peroxidase-conjugated secondary antibody using ECL detection reagents (GE Healthcare Life Sciences).

Supporting results

Clinical features of the patient

A 72-year old female reported in the last 6 months a weight loss of approx. 10 kg with a feeling of increasing fatigue. The patient underwent an upper abdominal ultrasound showing a lesion about 5 x 6 cm without bone metastasis but several nodules in the lungs. CT showed a 9 cm expansion in the right liver lobe that was analyzed by biopsy and operated. Laboratory tests performed at the day before the operation showed the following results: CA19-9, 5700U/ml; AFP, normal; slight anemia (Hb 10.9 g/dL), slight thrombocytosis (463,000 cells per mm^3) monocytosis (1200 cells per mm^3), alkaline phosphatase (314 U/L), GGT (137U/L), fibrinogen (1000 mg/L) and CRP (147mg/L). The patient was not subjected to chemotherapy. One month after surgery, the patient was hospitalized again because of dyspnoea and thoracic pain. CT of thorax showed a massive pleural effusion as well as multiple nodular lesions measuring up to 7 mm. Due to the poor general condition, no chemotherapy was undertaken, and the patient died 4 weeks after surgery.

Pathological features of the tumor

The tissues were fixed in buffered formalin and embedded in paraffin and 2-4 µm thick sections were subjected to histopathological and immunohistochemical analyses. The eight different tumor blocks either showed predominantly epithelial structures, classified as the carcinomatous component (Carc), or satellite nodules with predominant undifferentiated, spindle-like cells that were classified as the sarcomatoid component (Sarc). Furthermore, in two blocks (blocks 3 and 5) a transition from the Carc to the

Sarc component was seen. These areas were designated as transitional component (Trans). The surgically resected liver tumor was diagnosed as intrahepatic cholangiocarcinoma with a sarcomatoid component. A lymph node metastasis (Met) showed sarcomatoid features (an overview on the FFPE blocks and their morphological features is provided in **Supporting Fig. S3**).

Immunohistochemical characterization of the tumor

Markers for biliary epithelial cells (keratin-8 and keratin-18, keratin-7, keratin-19 and MUC-1) and cell-cell adhesion proteins (CLDN4, E-cadherin, β-catenin) were strongly expressed in the carcinomatous component, indicating polarized and epithelial differentiation (**Supporting Table S3**). The sarcomatoid tumor component and the metastasis showed weak or negative expression of the above-mentioned proteins, while displaying increased expression of the mesenchymal marker vimentin. The sarcomatoid component and the metastasis showed increased staining with the cellular proliferation marker Ki67, which suggests an increased malignant phenotype (**Supporting Table S3**).

Furthermore, we analyzed markers reported to be associated with cholangiocarcinoma progression or poor patient survival. Membranous expression of the HGF receptor (c-MET) and CD44 and overexpressed p53 in the nucleus were observed in all tumor components. Carcinoembryonic antigen (CEA) staining was confined to the carcinomatous component with no expression in sarcomatoid component and the metastasis (**Supporting Table S3**).

IHC analysis for hepatic stem/progenitor cell markers (NCAM1, CD34, CgA, CD117 and Nestin) and hepatocytic lineage associated markers (Hep Par 1 and AFP) were negative in tumor cells (**Supporting Table S3**). The results suggest that the primary tumor was derived from the intrahepatic biliary duct epithelial cells.

Characterization of the tumor cell culture

Initially, 5 culture dishes with cells outgrowing from the tumor were generated. The primary tumor cell culture most likely was a mixture of different cell types, such as malignant, non-malignant epithelial and mesenchymal cells. However, cells of only one of the dishes could be further propagated (dish C which gave rise to clone C), whereas cells of the other dishes stopped dividing, most likely because they were non-tumor cells.

To exclude the possibility that the clone C isolated from tumor tissue is not of epithelial origin or is a mixture of epithelial and non-epithelial cells, we performed FACS characterization of early passage clone C (passage 10) using the following antibodies: positive control: EpCAM (epithelial cell marker), CD68 (macrophage marker), CD19 (B-cell marker), CD3 (T-cell marker), Ve-cadherin (endothelial marker). Furthermore, to exclude the presence of cells with muscle differentiation we have performed immunofluorescence staining on cultured cells with antibodies directed to alpha smooth muscle actin (alpha-SMA; data not shown). As expected, the Ep-CAM antibody stained cells positively, while markers to lymphocytes, macrophages, endothelial and smooth muscle cells were negative (**Supporting Fig. S4**).

Therefore, all further studies were performed with cells derived from the primary culture dish C and cellular sub-clones generated by single cell cloning. Phenotypic characterization of cells derived from dish C by using antibodies to keratins-8, 18, 19 and 7 (to distinguish biliary epithelial cells from hepatocytes) showed heterogeneous keratin-7 expression. Therefore, our first hypothesis to explain different phenotypes was that the culture is a mixture of different cell types. To test this hypothesis, we have performed single cell cloning. Surprisingly, most of the sub-clones established from single cells showed also a mixture of phenotypes, which led to another hypothesis that the obtained tumor cell culture contains tumor stem cells that may differentiate into phenotypically different cells. However, further phenotypic characterization did not confirm stemness of the tumor cell culture, but some markers indicated a possible relation to epithelial to mesenchymal transition. Further characterization of the phenotypes and functional studies led finally to the conclusion that the established

tumor cell culture and their derived sub-clones show stochastic phenotype switching (for an overview see **Supporting Fig. S1**). There is multiple evidence that the isolated cell clones are derived from the tumor and show features of malignancy, such as the ability to develop cell clones from single cells, tumorigenicity in nude mice xenografts, and the fact that the same genetic variants were identified by NGS in the tumor and the derived cultured sub-clones.

Supporting Figures

Supporting Fig. S1. Graphical abstract

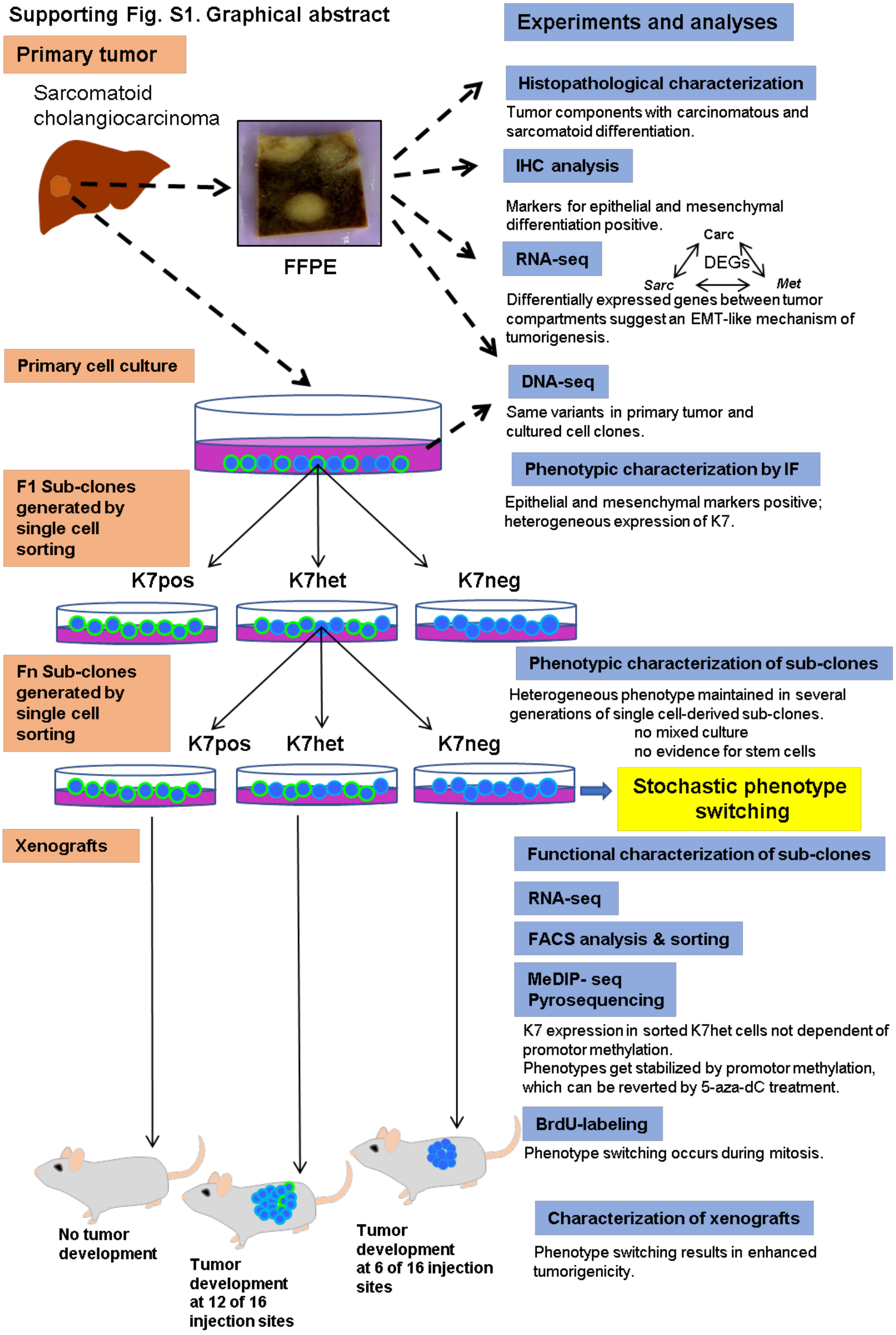

Supporting Fig. S2. Representative tissue blocks and control H&E stained slides used for isolating RNA from different tumor components.

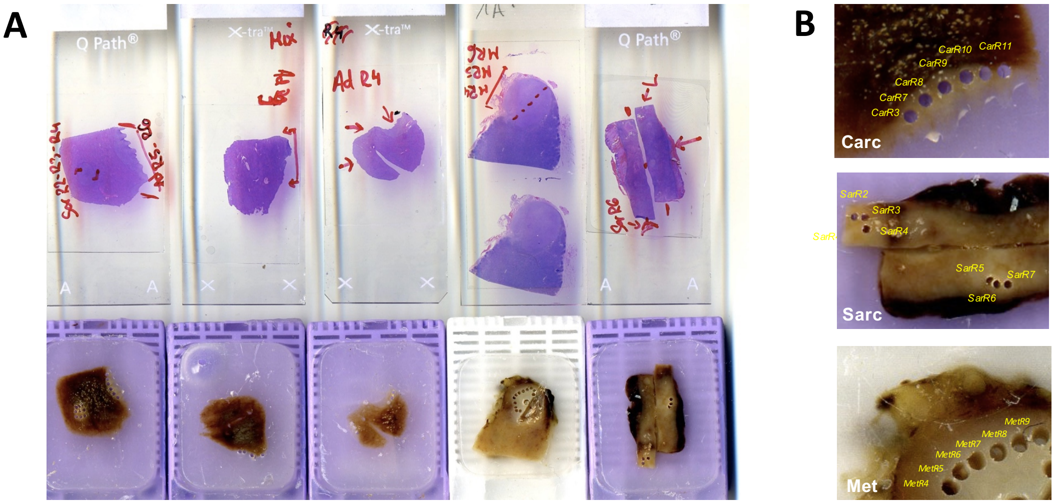

A) FFPE tumor blocks and corresponding hematoxylin and eosin (HE) stained slides used for isolating RNA from different tumor components and metastasis for RNA-sequencing. B) FFPE blocks with tumor areas with carcinomatous component (Carc), sarcomatoid component (Sarc) and lymph node metastasis (Met) showing areas with core holes from which RNA was isolated.

Supporting Fig. S3. Overview of samples analyzed from the sarcomatoid cholangiocarcinoma.

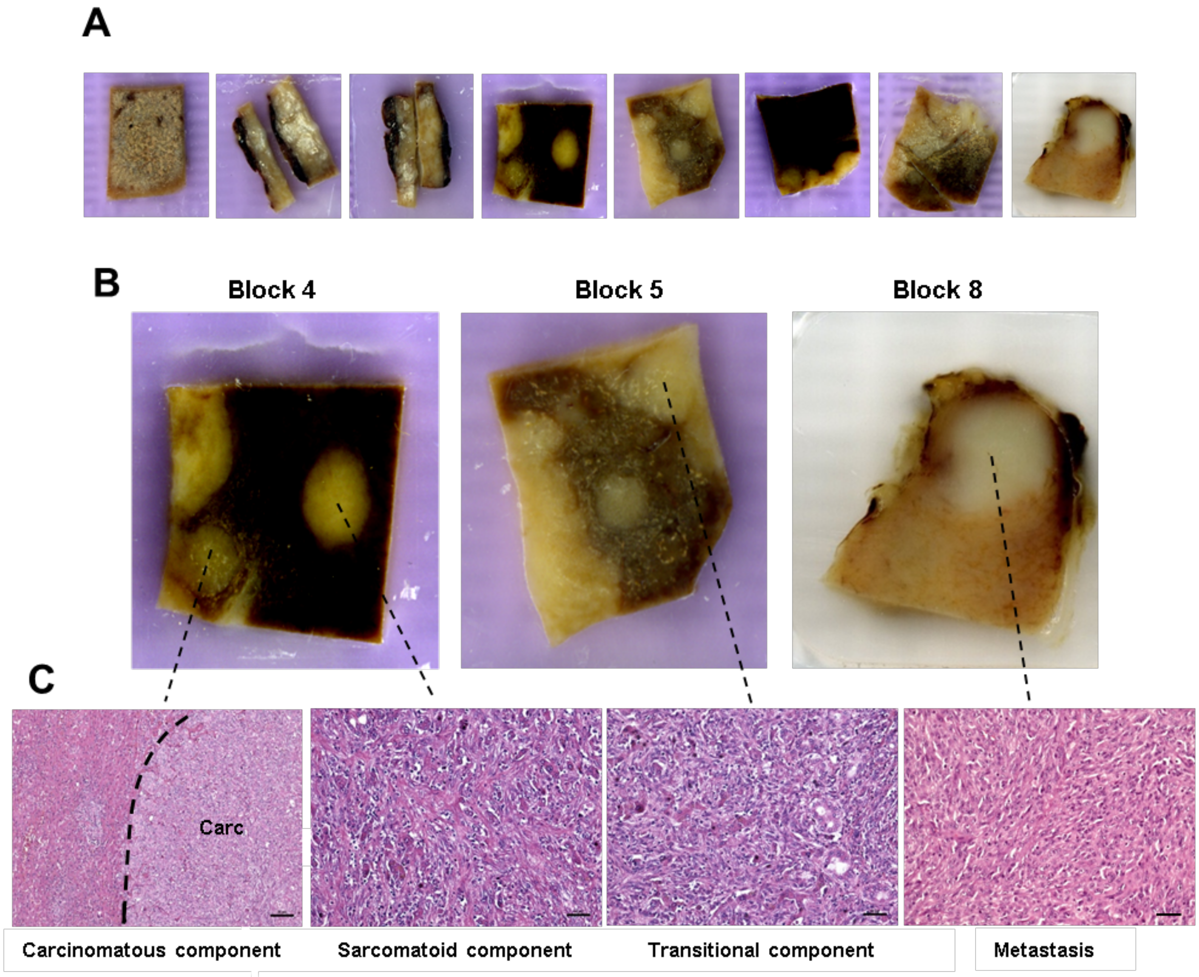

A) FFPE blocks used for IHC and RNA-seq analysis. Carcinomatous features are found in tumor blocks 4, 5, 6 and 7; sarcomatoid features are found in tumor blocks 2, 3, 4, 5 and 8 (metastasis); Transitional features are found in tumor blocks 3 and 5. Non-neoplastic liver=Block 1. B and C) Higher magnification of distinct areas of the tumor displaying carcinomatous component (Block 4; dashed line marks the border between non-neoplastic liver and the carcinomatous component), sarcomatoid component (Block 4) and transitional component (Block 5) with mixed carcinomatous and sarcomatoid elements. Block 8 with the lymph node metastasis showing sarcomatoid differentiation. Scale bar: 50 µm

Supporting Fig. S4. Phenotypic characterization of clone C by FACS analysis.

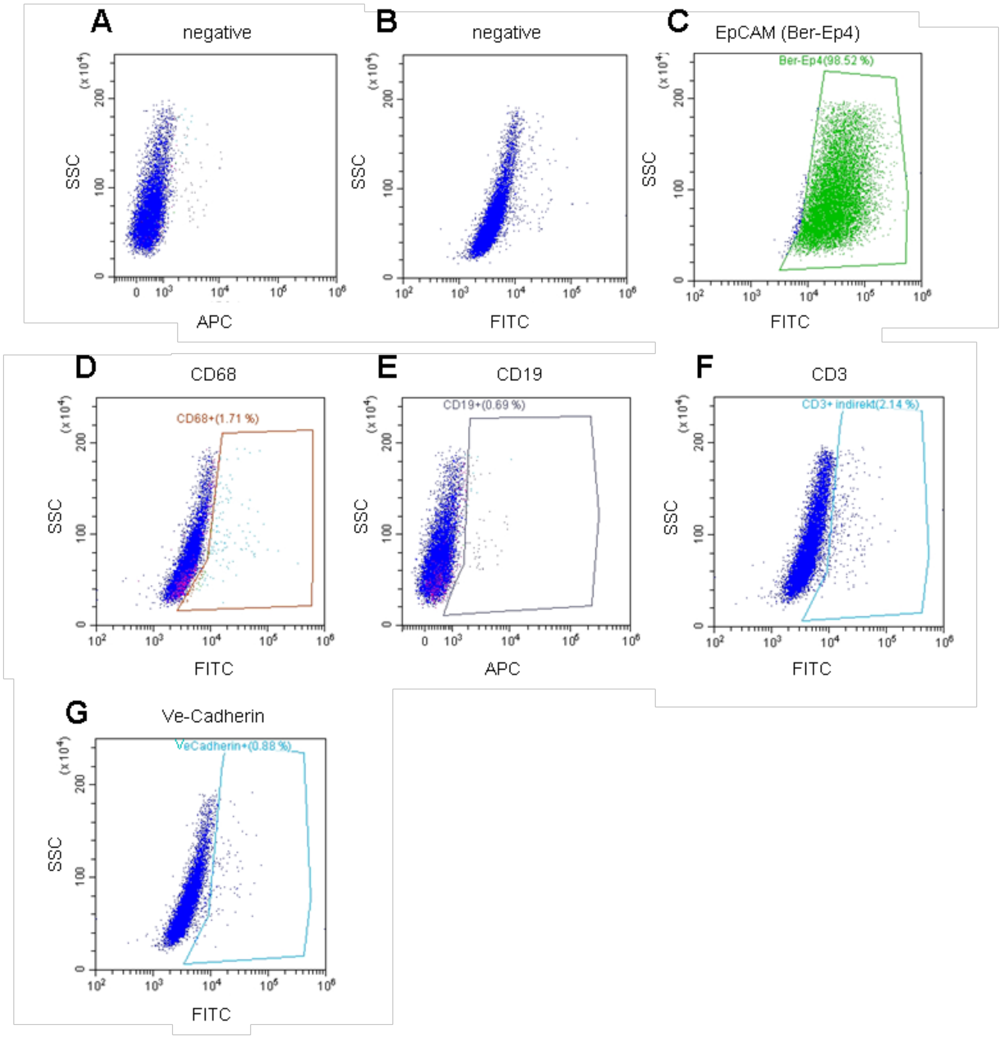

Cultured cells of clone C were analyzed by FACS for expression of differentiation markers. A-B) Negative controls: A) unstained cells, B) secondary fluorescence antibody only; C) Positive control: epithelial marker (EpCAM); D) macrophage marker (CD68); E-F) immune cells markers (CD19 and CD3); G) endothelial cell marker (Ve-Cadherin). (APC: allophycocyanin; FITC: fluorescein isothiocyanate).

Supporting Fig. S5. Variant frequency in the primary tumor (Sarc), K7pos and K7neg sub-clones.

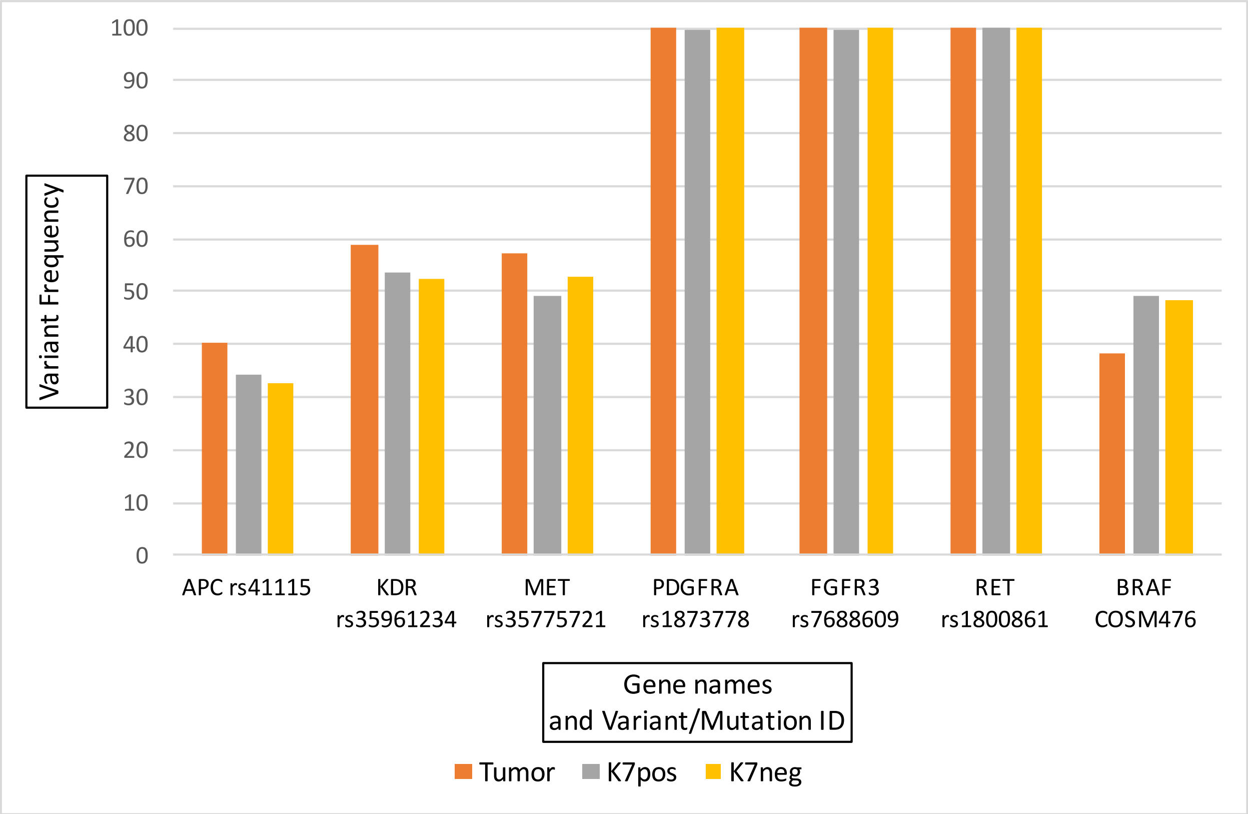

Representative variant frequencies in K7pos and K7neg sub-clones and primary tumor (Sarc). Gene names and Variant/Mutation ID are displayed on the horizontal axis.

Supporting Fig. S6. Immunofluorescence characterization of different keratin-7 sub-clonal phenotypes.

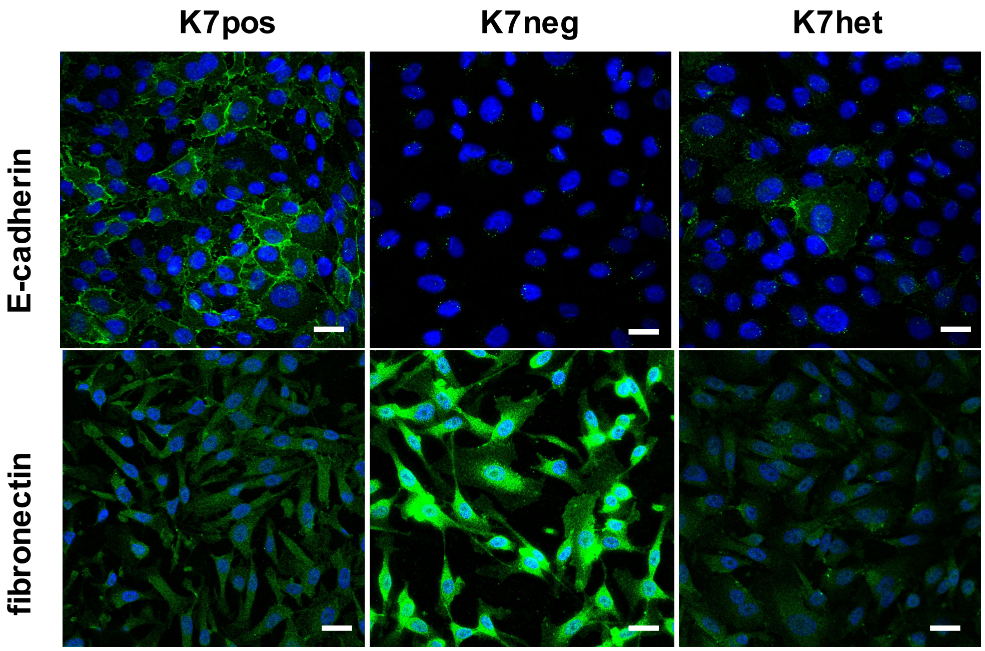

Immunofluorescence microscopic characterization of different keratin-7 sub-clonal phenotypes using antibodies against E-cadherin and fibronectin (green). Nuclei were stained with DAPI. Scale bar: 20 μm.

Supporting Fig. S7. Keratin-7 expression is reactivated upon treatment with 5-aza-deoxycytidine (5-aza-dC).

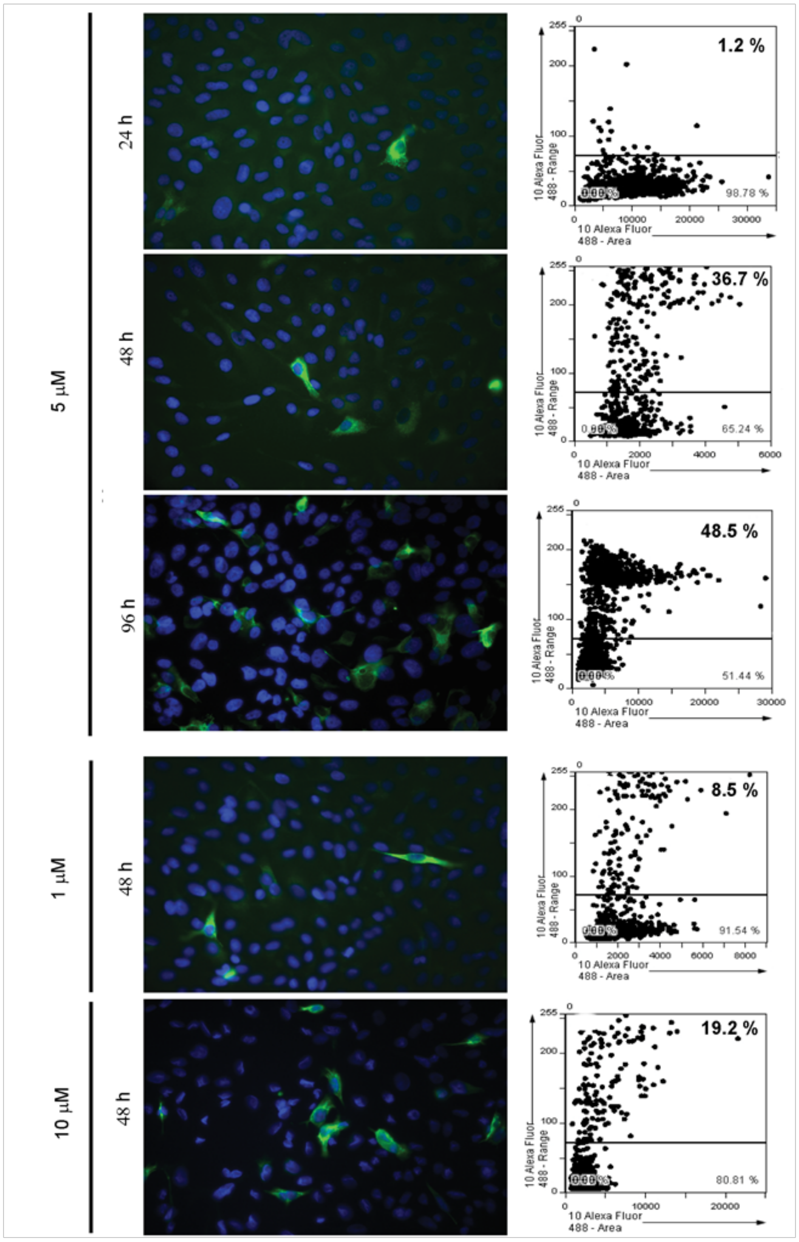

Immunofluorescence-stained cells representing time and dose dependent re-expression of keratin-7 protein in K7neg clones after 5-aza-dC treatment. Right: Scatter plots representing single cells with re-expressed KRT7 protein relative to nuclear DAPI positive cells. Cells were analyzed with TissueFAXS in each of the conditions. The percentages of positive cells were derived from the upper right corner in each scatterplot determined by the threshold lines and represent averages of at least 30,000 counted cells.

Supporting Fig. S8. Expression of EMT transcription factor, Zeb1 in different primary tumor components and mouse xenograft.

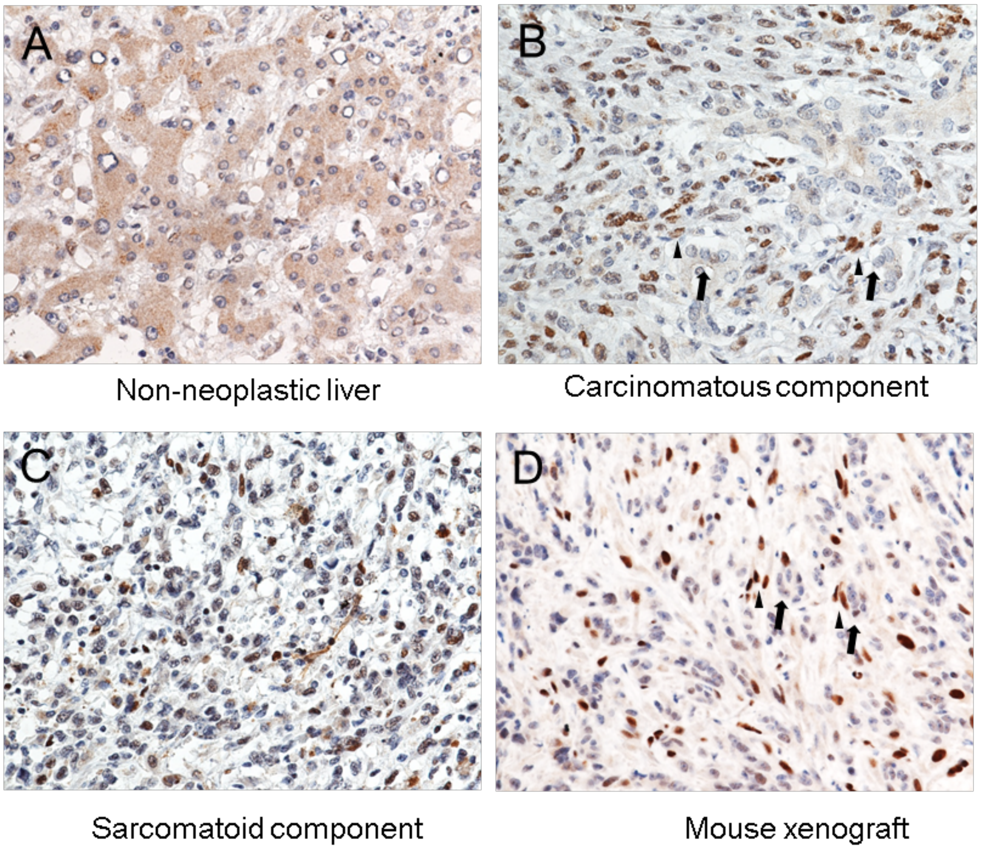

A-D) Immunohistochemical detection of Zeb1 expression in different primary tumor components and mouse xenograft. A) Non-neoplastic liver; B) Carcinomatous tumor component; C) Sarcomatous tumor component; E) Nude mouse tumor xenograft. Arrows in B) and D) indicate tumor cells with epithelial differentiation which are negative for Zeb1, whereas cells in proximity with mesenchymal differentiation (arrowheads in B and D) are positive for Zeb1. Note that similar situations are seen in the carcinomatous component of the primary tumor and the mouse xenograft.

(Magnification 400x)

Supporting Fig. S9. CD146/MCAM expression in primary tumor components and cultured sub-clones.

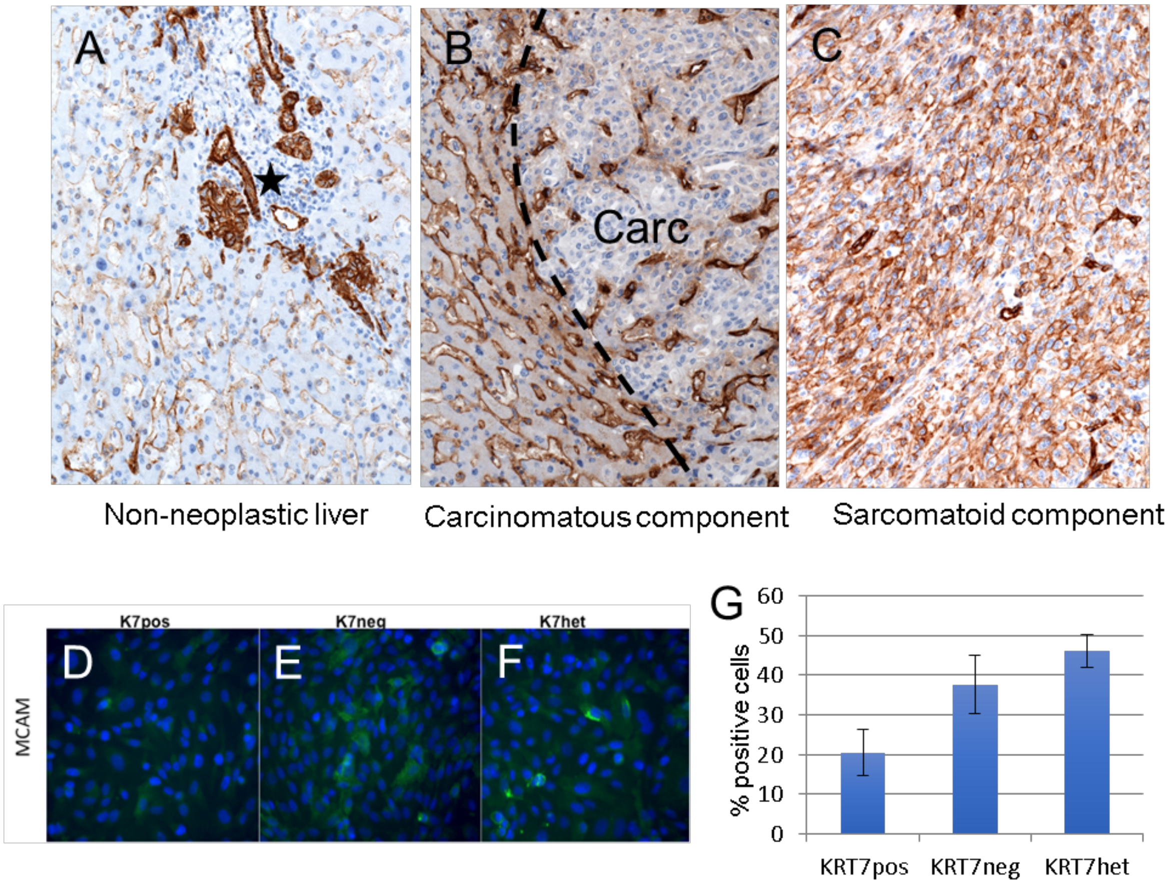

A-C) Immunohistochemical detection of CD146/MCAM in different primary tumor components A) Non-neoplastic liver, asterisk indicates portal tract; B) Carcinomatous tumor component [Carc]; dashed line indicates border between non-neoplastic liver and Carc; C) Sarcomatoid component. Note that CD146 shows no membranous staining in the Carc component whereas the sarcomatoid component shows strong membranous reaction (Magnification: 200x). D-E) IF characterization of CD146/MCAM expression (green) in K7 sub-clones showing different phenotypes (KRT7pos, KRT7neg, KRT7het), and DAPI (blue) as nuclear stain. G) FACS analysis of CD146/MCAM expression in clonal populations of the different keratin-7-expressing phenotypes (KRT7pos, KRT7neg, KRT7het), (n=6).

Supporting Tables

Supporting Table S1. List of antibodies used for immunohistochemistry immunofluorescence and FACS analysis.

Antibodies used for Immunohistochemistry on FFPE primary tumor samples					
**Antibody**	**Cat #**	**Company**	**Isotype**	**Dilutions**	**Block**
K7	M7018	DAKO	Mouse / IgG1	1:200	block 2-7
K8+18	NCL-L-CK8-TS1/MA5-12104	Novocastra/Thermo Fisher Scientific	Mouse / IgG1	1:200	block 4-8
K19	M0888	DAKO	Mouse monoclonal/ IgG1	1:200	block 4-8
VIM	61013	Progen	mouse monoclonal /IgG2a kappa	1:100	block 4-8
c-MET	ab51067	Abcam	Rabbit Monoclonal	1:200	block 1-8
Claudin 4	RB-9266-P0	Thermo Fisher Scientific	Rabbit Polyclonal	1:50	block 4-6, and 8
Muc1	NCL-MUC-1	Novocastra	Mouse monoclonal	1:100	block 1, 7 and 8
E-cadherin	610181	BD Transduction LaboratoriesTM	Mouse IgG2a	1:200	block 4-8
β-catenin	610153	BD Transduction LaboratoriesTM	Mouse IgG1	1:200	block 2-8
CEA	MS613-P0	Thermo Fisher Scientific	mouse monoclonal /IgG2a kappa	1:200	block 2-8
CD44	MS-668-P0	Thermo Fisher Scientific	mouse IgG2a	1:400	block 4-8
P53	M7001	DAKO	mouse monoclonal	1:100	block 2, 5, 7 and 8
Ki67	M7240	DAKO	mouse monoclonal	1:200	block 2-8
NCAM1	NCL-L-CD56-1B6	Novocastra	mouse monoclonal IgG1	1:50	block 2-8
CgA	MS-382-P0	Thermo Fisher Scientific	mouse monoclonal/IgG1	1:200	block 1, 4-7
CD117	A4502	DAKO	rabbit polyclonal	1:200	block 3-7

CD34	MS-363-P0	Thermo Fisher Scientific	mouse monoclonal IgG1	1:200	block 4,5 and 7
Nestin	sc23927	Santa Cruz	mouse monoclonal	1:50	block 4-8
EpCam (ESA)	NCL-ESA	Novocastra	mouse monoclonal	1:100	block 4-8
Hep Par 1	M7158	DAKO	mouse monoclonal	1:50	block 4-7
AFP	A0008	DAKO	rabbit polyclonal	1:100	block 4-7
Zeb1	NBP1-05987	Thermo Fisher Scientific	Rabbit IgG	1:50	block 2,3
Muc18	ab75769	Abcam	Rabbit monoclonal	1:200	block 4,7 and 8
**Antibodies used for staining cells grown on coverslips**					
K7	M7018	DAKO	Mouse / IgG1	1:200	
K8	NCL-L-CK8-TS1/	Novocastra	Mouse / IgG1	1:200	
K18	MA5-12104	Thermo Fisher Scientific	Mouse / IgG1	1:200	
K19	M0888	DAKO	Mouse / IgG1	1:200	
VIM	61013	Progen	mouse monoclonal /IgG2a kappa	1:50	
β-catenin	610153	BD Transduction LaboratoriesTM	Mouse IgG1	1:200	
EpCam (ESA)	NCL-ESA	Novocastra	mouse monoclonal	1:100	
E-cadherin	MS-9470-P0	Thermo Fisher Scientific	Mouse monoclonal	1:100	
Zeb1	NBP1-05987	Thermo Fisher Scientific	Rabbit IgG	1:50	
Muc18	ab75769	Abcam	Rabbit monoclonal	1:200	
anti-BrdU	ab6326	Abcam	Rat monoclonal	1:250	
alpha-SMA	ab5694	Abcam	Rabbit polyclonal	1:100	
**Antibodies used for FACS analysis**					
Anti- Human IL-13 R alpha 2	#FAB614F	R&D Systems	goat	1:50	
K7	M7018	DAKO	Mouse / IgG1	1:100	
Muc18	ab75769	Abcam	Rabbit monoclonal	1:200	

Ve-Cadherin	sc9989	Santa Cruz	mouse monoclonal	1:50	
CD68	M0814	DAKO	mouse monoclonal	1:25	
CD19	555415	BD Pharmingen	Mouse monoclonal-APC-conjugated	1:20	
CD3	M7254	DAKO	mouse monoclonal	1:30	
EpCAM/ Ber-Ep4	M0804	DAKO	mouse monoclonal	1:20	

Supporting Table S2. List of differentially expressed genes (DEGs) in the tumor

DEGs between the sarc/carc tumor component are shown in (A) and between the met/carc tumor component in (B) arranged by log2 fold change. Criteria for selection DEG: minimum expression level: 1;; minimum fold change: 2.0;; p--value (adjusted)<0.01.

**A. List of differentially expressed genes between the sarc/carc tumor component**			
	**Gene**	**log2FoldChange (sarc/carc)**	**p-value (adjusted)**
1	TMPRSS11E	-8.665612296	3.71E-22
2	TCN1	-8.600737812	1.18E-20
3	PRR15	-8.250262349	1.12E-15
4	KLK6	-8.08466757	2.29E-07
5	TSPAN8	-7.905439698	4.30E-13
6	COL9A3	-7.733693975	1.48E-40
7	TMPRSS4	-7.562962634	4.20E-34
8	SERPINB5	-7.292289518	3.49E-38
9	C19orf21	-6.957159596	3.31E-31
10	MUC16	-6.914971424	1.22E-32
11	AGR2	-6.835966181	4.53E-22
12	TSPAN1	-6.807146054	5.44E-31
13	SFTA2	-6.695940593	6.29E-07
14	HTR2C	-6.63598714	2.58E-07
15	GABRP	-6.62517074	2.30E-29
16	CLDN4	-6.50402004	2.84E-59
17	CAPN8	-6.474450358	2.79E-26
18	ANKRD56	-6.348853214	5.29E-14
19	NIPAL1	-6.323210836	2.98E-13
20	ENTPD2	-6.286637804	1.14E-08
21	SLC9A2	-6.27448586	2.67409E-04
22	SMEK3P	-6.238174304	6.60E-08
23	FAM83A	-6.15665022	6.60E-15
24	ERN2	-6.079652513	3.21E-13
25	ISM2	-6.044545801	0.009138702

DEGs between the sarc/carc tumor component are shown in (A) and between the met/carc tumor component in (B) arranged by log2 fold change. Criteria for selection DEG: minimum expression level: 1;; minimum fold change: 2.0;; p--value (adjusted)<0.01.

26	TSPAN2	-5.997179733	9.20E-13
27	HOXC13	-5.956297425	0.008934244
28	OVOL2	-5.912679725	0.008784394
29	FIBCD1	-5.822288526	3.87E-32
30	CEACAM6	-5.818431417	3.12E-32
31	RAB25	-5.803280422	1.65E-32
32	SLC6A14	-5.796411031	1.78E-09
33	CDH3	-5.782774932	4.25E-35
34	LOC100505967	-5.764531769	9.19E-06
35	OVOL1	-5.763924095	1.27E-05
36	C20orf151	-5.734745248	0.00273725
37	CDS1	-5.642185983	3.86E-30
38	CST4	-5.63872224	0.006385414
39	TACSTD2	-5.626638851	1.34E-71
40	TFPI2	-5.613937855	2.06E-09
41	KLK7	-5.541446316	0.006617849
42	MAGEA1	-5.525216262	9.71E-07
43	EHF	-5.488464721	2.25E-88
44	VGLL1	-5.455496847	4.79E-06
45	FA2H	-5.425421177	4.61E-18
46	MMP7	-5.288475673	2.85E-62
47	CDH19	-5.273943029	0.00192423
48	VENTXP1	-5.20386688	5.61E-07
49	IL1A	-5.155719399	4.23E-07
50	CACNG4	-5.147098556	4.80E-05
51	GPR143	-5.145778725	0.002142726
52	MAL2	-5.087505353	4.60E-70
53	MUC5B	-5.069712981	1.24E-09

DEGs between the sarc/carc tumor component are shown in (A) and between the met/carc tumor component in (B) arranged by log2 fold change. Criteria for selection DEG: minimum expression level: 1;; minimum fold change: 2.0;; p--value (adjusted)<0.01.

54	STYK1	-5.058063489	3.05E-12
55	FBN3	-5.008891492	7.70761E-04
56	CCDC64B	-4.949995097	6.07E-05
57	EPCAM	-4.919498903	1.66E-57
58	VWA2	-4.881741625	7.62E-12
59	FGF19	-4.857412913	8.95E-05
60	BARX2	-4.850800071	2.80E-10
61	RBM20	-4.847465841	0.004409511
62	PTPRZ1	-4.838745904	7.82E-07
63	SCNN1A	-4.81203655	2.79E-16
64	C19orf59	-4.79235889	2.53130E-04
65	FAAH2	-4.775474825	6.35E-06
66	FAM83B	-4.756851418	4.46E-14
67	ESRP1	-4.746772057	3.49E-38
68	SLPI	-4.744053057	3.24E-10
69	CDH1	-4.739384587	5.02E-39
70	LYPD3	-4.729288927	4.09645E-04
71	AIM1L	-4.727419901	4.21E-19
72	HPGD	-4.720660278	2.77E-28
73	FUT3	-4.718579507	4.99E-17
74	SGPP2	-4.706963006	1.64E-10
75	KLK10	-4.703633571	7.29E-30
76	PRSS22	-4.689293148	8.97E-17
77	FXYD3	-4.687591241	1.07E-13
78	EPHX3	-4.662072618	3.91E-06
79	AP1M2	-4.657962719	8.30E-21
80	KRT4	-4.647086157	0.004096275
81	BIK	-4.625681066	6.75E-10

DEGs between the sarc/carc tumor component are shown in (A) and between the met/carc tumor component in (B) arranged by log2 fold change. Criteria for selection DEG: minimum expression level: 1;; minimum fold change: 2.0;; p--value (adjusted)<0.01.

82	LCN2	-4.620507905	1.12E-12
83	EPS8L1	-4.595820846	1.25E-19
84	AKR7A3	-4.587322778	4.39E-05
85	MACC1	-4.583771112	8.62E-71
86	PROM1	-4.528517839	9.14E-38
87	HRASLS5	-4.524643016	0.004019167
88	GPR35	-4.518034288	3.77E-21
89	CRB3	-4.4961355	9.13E-06
90	C2orf54	-4.454067521	1.61488E-04
91	FAM83E	-4.447025271	1.22E-08
92	SPINT1	-4.365721488	5.71E-24
93	SCEL	-4.364590278	2.06E-38
94	INPP4B	-4.363754584	9.83E-26
95	PDZK1IP1	-4.345349547	1.81E-05
96	FREM2	-4.335409169	8.96E-06
97	PADI1	-4.30974524	1.75E-10
98	TFAP2C	-4.305789457	0.00417762
99	PPP1R9A	-4.2764861	2.10E-38
100	CRABP2	-4.233908784	3.99E-11
101	GCNT3	-4.2282858	1.96E-08
102	KLK11	-4.220028755	3.16E-16
103	ACPP	-4.203543272	8.67E-05
104	GRHL2	-4.200681028	3.74E-09
105	ITGB6	-4.165395645	6.75E-10
106	WNT7A	-4.164992252	3.03E-13
107	MUC15	-4.164145752	5.45E-07
108	CRLF1	-4.159838434	4.30026E-04
109	SCIN	-4.156391996	5.02E-06

DEGs between the sarc/carc tumor component are shown in (A) and between the met/carc tumor component in (B) arranged by log2 fold change. Criteria for selection DEG: minimum expression level: 1;; minimum fold change: 2.0;; p--value (adjusted)<0.01.

110	GPR110	-4.153282359	3.29E-10
111	BSPRY	-4.139685581	1.99E-17
112	KRT19	-4.127299852	8.93E-29
113	CST6	-4.09650189	0.006234209
114	CX3CL1	-4.071442558	1.31E-13
115	PLEKHG6	-4.068637967	6.36E-12
116	SLC5A1	-4.052049428	1.25E-06
117	KRT6A	-4.032885797	5.14714E-04
118	ADAM2	-4.017393084	0.00798659
119	EPHB6	-4.011533127	2.75E-24
120	PCDHA12	-4.004527714	0.003938307
121	GALNT3	-3.997506659	9.99E-30
122	CLDN7	-3.993235121	2.59E-30
123	P2RY2	-3.967357946	2.50E-15
124	KRT6B	-3.956715258	3.44E-06
125	COL17A1	-3.943564175	5.54E-10
126	TGFA	-3.94308254	2.27E-32
127	CALB2	-3.942865326	1.19E-13
128	LMO3	-3.906502548	1.85265E-04
129	SPDEF	-3.890252136	0.003435408
130	DKK1	-3.874130344	0.005685256
131	DPP4	-3.870386137	1.44E-24
132	KRT7	-3.863320105	2.25E-16
133	C1orf116	-3.852750039	1.46E-16
134	ANKRD22	-3.837433997	3.06E-16
135	POU6F2	-3.831981575	6.83E-05
136	TOX3	-3.816919661	4.38E-10
137	PLS1	-3.806834775	7.14E-16

DEGs between the sarc/carc tumor component are shown in (A) and between the met/carc tumor component in (B) arranged by log2 fold change. Criteria for selection DEG: minimum expression level: 1;; minimum fold change: 2.0;; p--value (adjusted)<0.01.

138	LEMD1	-3.80361826	1.32995E-04
139	PRSS8	-3.801775102	2.77E-26
140	ELF3	-3.790268526	1.78E-18
141	PPP1R14C	-3.784390665	4.87E-08
142	MYO5C	-3.780351699	5.05E-29
143	PKP1	-3.775631512	1.10E-05
144	KIAA1244	-3.77014123	4.05E-24
145	PRR15L	-3.76913875	1.24E-06
146	SLC15A1	-3.767256225	1.02E-28
147	PKD1L2	-3.757781761	9.12E-06
148	CYP24A1	-3.737512761	1.20E-05
149	PSCA	-3.704159095	3.68955E-04
150	PHACTR3	-3.703195228	6.57450E-04
151	FAM83F	-3.694809179	1.33E-05
152	CLIC5	-3.691233184	9.71E-19
153	CAPN13	-3.687091278	5.23660E-04
154	LOC100131726	-3.679317793	0.002028034
155	ARL14	-3.670895188	8.74E-05
156	SPINT2	-3.664881115	1.57E-34
157	RHOV	-3.662812142	4.11E-13
158	CBLC	-3.662172287	1.48E-08
159	SPRR3	-3.647703861	0.003228582
160	EPN3	-3.639024508	2.32E-07
161	C1orf172	-3.63849436	0.002203766
162	SMPDL3B	-3.632684345	1.91548E-04
163	TFCP2L1	-3.625060277	7.48E-23
164	NLGN4X	-3.623400815	7.63034E-04
165	IRF6	-3.621957295	2.62E-32

DEGs between the sarc/carc tumor component are shown in (A) and between the met/carc tumor component in (B) arranged by log2 fold change. Criteria for selection DEG: minimum expression level: 1;; minimum fold change: 2.0;; p--value (adjusted)<0.01.

166	SLITRK1	-3.616338948	1.97538E-04
167	NEURL3	-3.612219695	0.004196107
168	ANO3	-3.582121834	0.008847329
169	STK32A	-3.579799141	8.25478E-04
170	CEACAM1	-3.574742498	1.24E-27
171	ITGB4	-3.567422644	8.80E-13
172	LAMB3	-3.557605059	8.33E-45
173	SYT13	-3.549935578	2.52E-29
174	ZG16B	-3.541566221	0.001032238
175	TMEM30B	-3.53359546	1.09E-18
176	CGN	-3.531051228	4.43E-15
177	B3GNT3	-3.524665349	1.58E-33
178	PGBD5	-3.52404577	1.03697E-04
179	TJP3	-3.512106172	4.69E-12
180	WNK2	-3.507810777	3.42E-12
181	BCAS1	-3.505409742	6.36E-10
182	MPZL2	-3.50060015	5.84E-29
183	TMEM139	-3.494246675	1.99E-11
184	LPHN3	-3.48864558	9.15144E-04
185	MYH14	-3.45976031	5.30E-30
186	ANKRD1	-3.458023875	2.44E-12
187	CTSE	-3.450694813	5.72E-06
188	KRT8	-3.440860566	1.01E-21
189	ARHGEF38	-3.435847905	1.21E-13
190	CNGA1	-3.422267646	3.29E-06
191	RNF43	-3.393791165	1.32E-09
192	SOX2	-3.389750758	0.009073854
193	LOC100130705	-3.365348772	4.22E-06

DEGs between the sarc/carc tumor component are shown in (A) and between the met/carc tumor component in (B) arranged by log2 fold change. Criteria for selection DEG: minimum expression level: 1;; minimum fold change: 2.0;; p--value (adjusted)<0.01.

194	FRAS1	-3.357201179	3.39E-11
195	EYA2	-3.356731627	0.001709391
196	KCP	-3.351662536	3.47E-11
197	MYEOV	-3.347112853	7.62E-20
198	CNKSR1	-3.334251945	6.57E-09
199	KRT17	-3.312917929	9.43E-25
200	PADI2	-3.309643348	3.44E-07
201	SLC7A11	-3.301187067	3.92E-11
202	ST14	-3.290727373	5.25E-17
203	ABCA12	-3.283120815	1.96E-05
204	TMEM125	-3.278401309	1.33107E-04
205	KCNH8	-3.271022966	1.18E-05
206	WFDC2	-3.262955255	5.03E-05
207	SLC5A12	-3.25544164	2.09E-05
208	FAM160A1	-3.245834746	2.31E-14
209	PTGS2	-3.245014819	2.42E-05
210	SDR42E1	-3.233284575	2.58509E-04
211	DAPP1	-3.224016885	1.57E-11
212	TRIM29	-3.211989119	0.003754811
213	ENPP5	-3.21134067	4.62311E-04
214	IL1R2	-3.207800583	0.007205152
215	SYTL1	-3.205737398	9.44E-06
216	LLGL2	-3.204772508	3.31E-12
217	CYP4X1	-3.202782535	1.45E-06
218	IVL	-3.193526131	2.52304E-04
219	PROM2	-3.193412722	8.09E-16
220	LAMC2	-3.173853758	1.10E-20
221	TESC	-3.173076734	3.19E-17

DEGs between the sarc/carc tumor component are shown in (A) and between the met/carc tumor component in (B) arranged by log2 fold change. Criteria for selection DEG: minimum expression level: 1;; minimum fold change: 2.0;; p--value (adjusted)<0.01.

222	RASSF6	-3.167510769	9.32E-14
223	PLA2G4A	-3.164765132	2.60680E-04
224	CHST4	-3.163314448	8.44335E-04
225	DEFB1	-3.140284291	7.33355E-04
226	TMC4	-3.127079914	4.92E-14
227	FERMT1	-3.123930708	1.00E-19
228	HGD	-3.118757656	2.10E-06
229	SH3BGRL2	-3.114915211	4.13E-09
230	GREB1L	-3.114787322	2.88E-12
231	KCNK1	-3.106542249	1.79E-07
232	CEACAM5	-3.103994374	1.90478E-04
233	CTSL2	-3.086229297	4.22E-06
234	HHIP	-3.084221633	2.84E-06
235	HSD17B2	-3.080423417	1.15E-05
236	LIPC	-3.07795787	0.003490461
237	COBL	-3.069163048	7.61E-15
238	EDN1	-3.067803316	2.15E-08
239	MUC1	-3.059738529	1.93E-29
240	TNFRSF10C	-3.056175422	5.87571E-04
241	ZDHHC23	-3.050452653	1.32065E-04
242	ADAP1	-3.046039997	9.22E-08
243	EPS8L3	-3.027748261	1.56E-09
244	GRHL1	-3.02365459	2.88E-10
245	EREG	-3.015976493	9.87E-25
246	C9orf125	-3.009563939	0.002765025
247	WNT10A	-3.006899391	6.27E-06
248	LOC100131096	-2.991452893	3.80E-09
249	GDA	-2.989989902	3.30E-11

DEGs between the sarc/carc tumor component are shown in (A) and between the met/carc tumor component in (B) arranged by log2 fold change. Criteria for selection DEG: minimum expression level: 1;; minimum fold change: 2.0;; p--value (adjusted)<0.01.

250	VTCN1	-2.95623515	3.49E-08
251	C9orf140	-2.9534504	3.96E-05
252	PAK6	-2.947478674	4.19E-06
253	HOOK1	-2.934463278	7.91E-14
254	EPHA1	-2.933098875	5.13E-06
255	RASGEF1A	-2.925661839	0.003381239
256	GJB3	-2.916193487	2.57E-09
257	PLLP	-2.915874124	0.001630017
258	FAM174B	-2.912560725	4.62E-08
259	ICA1	-2.907445906	2.82E-11
260	KLK8	-2.90608835	0.002518738
261	CRISPLD1	-2.904749741	0.001291204
262	CYBA	-2.903587141	2.66E-10
263	SPINK5	-2.870233338	4.14E-08
264	UNC13D	-2.869900173	2.96E-12
265	SEMA3E	-2.862689882	1.50E-11
266	DRP2	-2.853320835	8.99782E-04
267	LOC641364	-2.838824637	1.06E-08
268	KRT18	-2.832292287	3.87E-07
269	LRRN1	-2.826797668	0.003004564
270	RAPGEF3	-2.820907193	2.22E-09
271	KRT13	-2.818952173	5.63634E-04
272	SHISA2	-2.805040508	1.14E-13
273	CLDN3	-2.793552612	4.82E-06
274	CHN2	-2.7915904	9.39E-06
275	PKP3	-2.780073774	4.01E-15
276	ESRP2	-2.769895839	6.96E-15
277	PLEKHA6	-2.769307685	1.44E-17

DEGs between the sarc/carc tumor component are shown in (A) and between the met/carc tumor component in (B) arranged by log2 fold change. Criteria for selection DEG: minimum expression level: 1;; minimum fold change: 2.0;; p--value (adjusted)<0.01.

278	DSP	-2.768032058	6.45E-35
279	CXCL5	-2.764514145	1.16E-05
280	ESCO2	-2.746771836	5.29856E-04
281	STEAP4	-2.739170253	1.20E-07
282	SPIN4	-2.735940521	3.76E-08
283	IGF2BP1	-2.734568319	2.46993E-04
284	RASAL1	-2.722965484	7.71E-06
285	KLHDC7A	-2.718347213	0.001013551
286	FLJ45974	-2.713035949	0.009278378
287	EVPL	-2.70610676	2.63E-06
288	DUSP4	-2.690509956	4.98E-25
289	ENPP4	-2.683990549	4.32E-11
290	TNFRSF11A	-2.68396065	0.001145441
291	PLAC8	-2.67715809	1.47471E-04
292	GRAMD1C	-2.666456842	2.52828E-04
293	MAOB	-2.654258724	6.66E-14
294	TM4SF18	-2.652392225	7.60E-10
295	C15orf41	-2.648948536	1.78E-06
296	C1orf126	-2.646635509	6.79196E-04
297	PBOV1	-2.642299332	4.65131E-04
298	DMBT1	-2.64018398	0.001812301
299	TMEM144	-2.624984264	2.02E-07
300	TMTC2	-2.623162481	1.74E-15
301	REPS2	-2.622437486	2.43E-09
302	AMIGO2	-2.615350697	1.19E-16
303	ELMO3	-2.602348178	3.51E-10
304	LAMA3	-2.596019299	2.34E-19
305	GJB4	-2.593694089	8.60E-09

DEGs between the sarc/carc tumor component are shown in (A) and between the met/carc tumor component in (B) arranged by log2 fold change. Criteria for selection DEG: minimum expression level: 1;; minimum fold change: 2.0;; p--value (adjusted)<0.01.

306	ILDR1	-2.593368365	0.00606903
307	CYP2S1	-2.585449715	0.00148422
308	TMPRSS2	-2.575820407	2.01E-06
309	VILL	-2.570453442	3.12E-12
310	LSR	-2.566308998	1.14E-07
311	CDCA7	-2.562301878	0.001893682
312	ALS2CL	-2.559128335	1.31E-13
313	GPR56	-2.553275208	4.18E-21
314	GINS2	-2.550353603	1.67E-05
315	MAGEC1	-2.550092871	0.004247562
316	PLEKHB1	-2.549455268	4.21E-05
317	FAM111B	-2.549015091	2.28E-07
318	LTK	-2.544379488	2.82E-06
319	WDR72	-2.543856757	2.36E-05
320	KIAA1543	-2.542767901	1.90E-06
321	LOC100128338	-2.535303454	3.33E-08
322	LOC219731	-2.528500363	0.004327074
323	HSH2D	-2.5280008	5.18E-05
324	SLC14A1	-2.523009442	3.71E-10
325	CD24	-2.521194482	1.34E-21
326	SMPD3	-2.517683563	0.005013923
327	ADAM23	-2.517034761	2.85E-05
328	DMKN	-2.511076213	6.05E-10
329	CDC42BPG	-2.499445224	2.71E-12
330	SHROOM2	-2.497448475	1.92E-11
331	PDE8B	-2.497161688	7.12E-05
332	ELOVL7	-2.482270463	1.08149E-04
333	PLCH2	-2.481570736	0.005420078

DEGs between the sarc/carc tumor component are shown in (A) and between the met/carc tumor component in (B) arranged by log2 fold change. Criteria for selection DEG: minimum expression level: 1;; minimum fold change: 2.0;; p--value (adjusted)<0.01.

334	SLC37A1	-2.47923727	5.08E-09
335	LOC100130899	-2.478403265	0.005580292
336	SAMD12	-2.469430044	1.17E-10
337	KLF5	-2.465409371	1.34E-17
338	GOLT1A	-2.462767112	0.001530782
339	TTC9	-2.461250866	1.47E-14
340	VAMP8	-2.459818788	2.14E-10
341	DACT2	-2.459497624	0.007046821
342	LOC439990	-2.439602243	0.001165846
343	DHRS9	-2.425768502	0.004245419
344	MGST1	-2.421656888	3.54E-07
345	PNPLA3	-2.419028589	1.21E-05
346	TOB1	-2.410242083	5.32E-14
347	NYNRIN	-2.403483109	8.93E-10
348	MIA2	-2.401894684	9.05E-06
349	C10orf81	-2.400305047	2.73E-06
350	MAP7	-2.393771719	1.04E-11
351	TPD52	-2.388464042	1.66E-11
352	LRRC16A	-2.383795279	5.85E-15
353	LIMCH1	-2.378607625	5.99E-11
354	ACOT11	-2.375384521	2.03557E-04
355	SOX13	-2.360857696	4.66E-11
356	PKHD1	-2.358222504	4.38E-19
357	VSIG1	-2.3398512	2.65104E-04
358	HS3ST1	-2.338607136	2.33E-07
359	ODZ1	-2.330462748	0.009597337
360	IL20RA	-2.325332905	3.04E-06
361	PAX9	-2.322844741	0.004554014

DEGs between the sarc/carc tumor component are shown in (A) and between the met/carc tumor component in (B) arranged by log2 fold change. Criteria for selection DEG: minimum expression level: 1;; minimum fold change: 2.0;; p--value (adjusted)<0.01.

362	LONRF2	-2.32259466	9.34E-08
363	AKR1C3	-2.318824869	0.004830186
364	A2ML1	-2.317301624	4.34E-07
365	ACSL5	-2.315091491	7.15E-13
366	NET1	-2.312797716	3.26E-05
367	SERPINB1	-2.305261737	2.97E-15
368	CYB5R2	-2.298504076	0.002142726
369	DTX4	-2.295443196	1.66E-11
370	EPDR1	-2.294802711	5.74E-07
371	FMN1	-2.292983419	5.00E-19
372	C20orf54	-2.28603363	0.001800008
373	FAM108C1	-2.27855399	8.71E-10
374	SH3TC2	-2.266325794	1.49E-12
375	EPHB3	-2.266232517	0.003100468
376	TM4SF1	-2.254499272	1.89E-23
377	RHPN2	-2.25213042	2.89E-10
378	ERBB3	-2.251119087	1.16E-16
379	CHDH	-2.249766831	4.03E-08
380	SH2D4A	-2.242007855	1.23E-09
381	TRPV6	-2.241780545	0.00694857
382	PPL	-2.241541428	3.16081E-04
383	PRKCZ	-2.24072779	6.24784E-04
384	CAPS	-2.239774799	4.84E-06
385	ATP1B1	-2.236696497	5.98E-18
386	ALDH1A3	-2.218289822	1.43E-10
387	THRB	-2.217564668	5.09E-06
388	UBXN10	-2.213385636	1.10494E-04
389	STRA6	-2.210211373	5.50E-09

DEGs between the sarc/carc tumor component are shown in (A) and between the met/carc tumor component in (B) arranged by log2 fold change. Criteria for selection DEG: minimum expression level: 1;; minimum fold change: 2.0;; p--value (adjusted)<0.01.

390	SYNGR2	-2.207250196	5.09E-16
391	NUP210	-2.195968139	2.57E-12
392	RAB11FIP1	-2.193997214	6.86E-08
393	SLC12A2	-2.193612203	6.71E-18
394	RPS6KA2	-2.192994481	1.34E-13
395	CCDC68	-2.191339564	4.12E-10
396	MUC20	-2.190511832	6.12436E-04
397	MANSC1	-2.188093039	9.85E-12
398	LY75	-2.181768948	1.96737E-04
399	XDH	-2.168231665	1.56E-07
400	DDAH1	-2.167214055	8.10E-10
401	SPNS2	-2.162058207	7.02110E-04
402	FOXQ1	-2.161668856	1.54E-06
403	RAB27B	-2.155116826	3.45E-10
404	RAP1GAP	-2.150000805	3.94E-11
405	PDE9A	-2.147977046	4.09645E-04
406	FHDC1	-2.147371726	1.57E-05
407	CXCL16	-2.146680886	1.04E-12
408	MAPK13	-2.143057846	1.28E-09
409	OXTR	-2.136387019	1.23E-09
410	FLJ23867	-2.134206765	2.89E-09
411	RAD51	-2.126405062	0.007136874
412	TNFRSF21	-2.120281459	3.79E-19
413	ADAM28	-2.119603882	6.23E-05
414	GPR126	-2.117771443	2.27E-11
415	C15orf48	-2.113609807	9.85E-05
416	NBEAL2	-2.111846613	6.21E-07
417	MAP3K1	-2.10745035	8.88E-14

DEGs between the sarc/carc tumor component are shown in (A) and between the met/carc tumor component in (B) arranged by log2 fold change. Criteria for selection DEG: minimum expression level: 1;; minimum fold change: 2.0;; p--value (adjusted)<0.01.

418	FAM110C	-2.106523326	6.75E-08
419	ANK1	-2.100754826	4.16E-11
420	POF1B	-2.096716177	3.37E-08
421	ANKRD18A	-2.088443131	1.07E-05
422	MCM10	-2.086857221	0.006846858
423	VANGL2	-2.081490719	7.52E-05
424	GCOM1	-2.078543742	5.43295E-04
425	TK1	-2.069711537	0.003838568
426	TC2N	-2.069700349	2.04E-06
427	ANO5	-2.062522272	0.007836554
428	BMP6	-2.060501977	5.23E-05
429	RGL3	-2.054940572	6.11E-07
430	FBP1	-2.047326393	7.52E-05
431	EZR	-2.038086292	7.62E-20
432	GPC4	-2.037804016	5.45E-07
433	FAM3C	-2.030964279	3.52E-18
434	GATA3	-2.019117269	3.64E-06
435	DEPTOR	-2.019093307	0.002903292
436	C8orf73	-2.017481666	1.16392E-04
437	LRRC1	-2.011675669	7.94E-08
438	RFC3	-2.009740044	0.008847329
439	TTC22	-2.00756484	0.001004486
440	CNNM1	-2.007149569	2.05481E-04
441	ZWINT	-2.002495105	0.001450924
442	FHL1	2.003594883	5.30E-12
443	THBD	2.007805846	7.49641E-04
444	MIR100HG	2.011360881	1.68E-07
445	MAPK8IP1	2.014422358	0.005905361

DEGs between the sarc/carc tumor component are shown in (A) and between the met/carc tumor component in (B) arranged by log2 fold change. Criteria for selection DEG: minimum expression level: 1;; minimum fold change: 2.0;; p--value (adjusted)<0.01.

446	MAP1B	2.01467703	8.24E-21
447	RPSAP52	2.019784152	0.001400425
448	WNT5A	2.023152705	2.53E-12
449	NF2	2.024624427	2.42E-20
450	LDLRAD3	2.026613028	6.92E-05
451	C14orf37	2.037740582	5.45E-05
452	RAB31	2.042338499	1.48E-17
453	THEMIS	2.045425894	8.60E-05
454	LRIG1	2.048482309	3.82E-12
455	FBN1	2.05546562	8.25E-18
456	C13orf33	2.057054344	5.25E-08
457	DIXDC1	2.057973554	1.48E-11
458	PTGER2	2.072422829	0.002241766
459	E2F7	2.072468537	3.10E-16
460	SMOC1	2.075485972	3.94E-11
461	GPR137C	2.076311219	0.006165817
462	NEXN	2.077761309	7.45E-06
463	ITK	2.084347965	0.002072893
464	FILIP1L	2.084797845	9.88E-16
465	SYT11	2.08561423	8.05E-14
466	SYNC	2.088602225	0.009562505
467	ANTXR1	2.091905036	1.23E-14
468	TRPC1	2.104510952	6.97E-07
469	FAM171A1	2.104812793	3.85E-09
470	CNIH3	2.111592401	4.20E-07
471	IL31RA	2.115425472	5.67E-12
472	RHOQ	2.118132831	1.49E-20
473	EGR1	2.125222941	0.005776832

DEGs between the sarc/carc tumor component are shown in (A) and between the met/carc tumor component in (B) arranged by log2 fold change. Criteria for selection DEG: minimum expression level: 1;; minimum fold change: 2.0;; p--value (adjusted)<0.01.

474	KHDRBS3	2.127562495	5.78E-07
475	CCL18	2.128894203	0.005479008
476	LTBP4	2.129700354	3.06E-17
477	DZIP1L	2.130482524	7.33E-08
478	PKIB	2.132371619	3.22E-07
479	MPP7	2.146117465	1.49E-09
480	SMAD1	2.165388278	2.34E-12
481	ABCC4	2.179871537	9.67E-12
482	DENND2A	2.185907246	0.001030923
483	FGF5	2.185927247	7.82E-19
484	RGMB	2.187354997	6.74E-15
485	C14orf132	2.193451863	8.69E-17
486	ADAMTS14	2.210947819	1.19E-15
487	STARD9	2.216513591	6.60E-07
488	CD2	2.225437553	7.51499E-04
489	DNM1	2.228953049	7.14E-13
490	PRICKLE1	2.241336425	9.12E-17
491	IGDCC4	2.243387281	8.29E-10
492	STEAP3	2.248410508	1.95E-20
493	DKK3	2.252321893	2.47E-16
494	TMOD2	2.25356133	9.50E-18
495	NEGR1	2.258119426	1.17E-07
496	ADAMTSL1	2.262773554	1.32E-13
497	BNC2	2.276238011	5.72E-20
498	KCNIP3	2.280142761	9.80E-05
499	MMP24	2.281440336	8.44E-15
500	SLC9A10	2.282137483	2.89E-08
501	FLNC	2.285187583	3.85E-21

DEGs between the sarc/carc tumor component are shown in (A) and between the met/carc tumor component in (B) arranged by log2 fold change. Criteria for selection DEG: minimum expression level: 1;; minimum fold change: 2.0;; p--value (adjusted)<0.01.

502	STX1B	2.300609434	1.04E-08
503	MGAT5B	2.310751475	4.69E-06
504	PLTP	2.319354973	3.86E-07
505	ITPKA	2.326633861	6.50E-13
506	AKAP12	2.331877357	2.50E-27
507	GLIPR2	2.335026855	3.57E-08
508	MFAP2	2.33990387	9.12E-05
509	TGFBI	2.348502633	5.79E-29
510	SACS	2.35996818	2.75E-24
511	GLIPR1	2.375250023	3.66E-13
512	ACPL2	2.390796447	1.12E-15
513	F13A1	2.407175533	5.15E-05
514	NDRG1	2.419621812	1.66E-13
515	CA12	2.424811592	9.38E-15
516	ANPEP	2.43174091	1.43E-13
517	PTPN22	2.432827149	1.04E-10
518	HES6	2.442850187	4.70363E-04
519	PRUNE2	2.444004376	4.01E-13
520	KIAA1462	2.449829327	3.58E-20
521	VEGFC	2.479226242	1.51E-15
522	STMN3	2.481005438	4.16E-17
523	NTNG1	2.483061715	2.70E-08
524	DAB2	2.486986248	6.07E-27
525	ARHGAP20	2.515896222	5.77E-09
526	SRPX	2.549241458	5.50E-09
527	PLEKHG4	2.550214988	1.81E-16
528	SPON2	2.554360488	2.52E-12
529	SCG5	2.55789735	1.46E-09

DEGs between the sarc/carc tumor component are shown in (A) and between the met/carc tumor component in (B) arranged by log2 fold change. Criteria for selection DEG: minimum expression level: 1;; minimum fold change: 2.0;; p--value (adjusted)<0.01.

530	SYNGR3	2.614538165	0.001193118
531	EPB41L3	2.624564541	6.42E-20
532	SUSD5	2.645341377	1.71E-21
533	PROCR	2.652952781	2.45E-07
534	PCDH9	2.674664197	1.87E-05
535	ADAMTS16	2.69937266	2.75E-12
536	LOC643401	2.701595492	2.10E-12
537	PTPRS	2.70832977	1.27E-26
538	PAPPA	2.741877028	3.25E-34
539	STC1	2.765599651	2.42E-34
540	B3GALT1	2.805523671	0.002933113
541	CREB5	2.823515478	1.30E-35
542	CORO2B	2.84338356	2.97E-13
543	FABP5	2.847352988	7.25E-06
544	MOXD1	2.8554335	2.35E-06
545	C6orf174	2.873982903	0.007185771
546	WNT2B	2.876579999	0.004318141
547	PCDHGA3	2.881084732	2.17E-07
548	NKD2	2.933727772	1.27E-06
549	ITGB3	2.944624265	3.80E-26
550	HRH2	2.946116555	7.55E-07
551	OLFML2A	2.952271099	1.19E-28
552	FAM126A	2.973327945	1.52E-23
553	SLC1A3	3.006183595	9.87E-25
554	PAEP	3.013981577	2.98E-15
555	TMEM158	3.030129328	2.76E-12
556	MEG3	3.065611646	1.17E-26
557	LTBP1	3.067809231	9.49E-48

DEGs between the sarc/carc tumor component are shown in (A) and between the met/carc tumor component in (B) arranged by log2 fold change. Criteria for selection DEG: minimum expression level: 1;; minimum fold change: 2.0;; p--value (adjusted)<0.01.

558	LOC84856	3.100715194	1.15714E-04
559	GZMA	3.207904494	0.001045284
560	LOXL1	3.217144411	1.49E-12
561	GLIS1	3.242058143	6.06345E-04
562	ADH1B	3.279332263	1.08E-05
563	ADAMTS6	3.281122767	4.05E-32
564	PDPN	3.293506765	7.88E-32
565	PLD5	3.293876082	1.27E-06
566	SPEG	3.325018743	1.85E-14
567	CCL21	3.351337548	2.74E-06
568	HS3ST3A1	3.353407503	2.08E-06
569	GZMK	3.354667889	0.008227942
570	BDKRB2	3.384770902	5.56E-24
571	CHST2	3.41459055	3.86E-14
572	CYGB	3.495156529	5.39E-10
573	H19	3.496659351	0.009336371
574	SCG2	3.528373135	0.004423994
575	SERPINE1	3.556704339	8.05E-52
576	EDIL3	3.642692787	1.50E-05
577	VAT1L	3.887162943	1.30E-16
578	IL6	3.91307322	3.19E-06
579	CPNE7	4.072697327	1.48E-17
580	GFPT2	4.152039521	1.54E-63
581	SPOCK3	4.183557267	3.51E-15
582	MIAT	4.257458532	2.25E-50
583	CLMP	5.037689462	2.11E-63
584	DNER	5.368251052	6.67E-40
585	SLN	5.836004586	1.83E-16

DEGs between the sarc/carc tumor component are shown in (A) and between the met/carc tumor component in (B) arranged by log2 fold change. Criteria for selection DEG: minimum expression level: 1;; minimum fold change: 2.0;; p--value (adjusted)<0.01.

**B. List of differentially expressed genes between the met/carc tumor component**			
	**Gene**	**log2FoldChange (met/carc)**	**p-value (adjusted)**
1	GABRP	-11.65111051	3.94E-58
2	MAL2	-10.83093148	2.04E-203
3	OLFM4	-10.79603934	9.92E-09
4	LCN2	-10.56392697	2.34E-32
5	SLPI	-10.46227389	7.35E-24
6	LBP	-10.3275365	1.02E-27
7	KRT19	-10.0364886	1.46E-91
8	TMPRSS4	-9.42581794	6.78E-51
9	KLK6	-9.153254901	1.72E-10
10	GDA	-9.098246896	7.43E-43
11	CDH1	-9.089218136	4.91E-91
12	FGA	-9.035212153	1.91E-27
13	GPR110	-8.947882555	6.62E-25
14	COBL	-8.914823621	8.34E-42
15	MUC16	-8.868953559	2.61E-52
16	FGG	-8.813138807	3.32E-21
17	KRT6B	-8.621494999	3.86E-15
18	PROM1	-8.614655762	5.53E-106
19	C8B	-8.600069432	4.99E-15
20	CAPN8	-8.412537637	1.36E-49
21	MUC15	-8.382204796	5.95E-15
22	WFDC2	-8.338005674	9.81E-15
23	CLDN4	-8.328309686	4.78E-89
24	AP1M2	-8.263239176	6.79E-49

DEGs between the sarc/carc tumor component are shown in (A) and between the met/carc tumor component in (B) arranged by log2 fold change. Criteria for selection DEG: minimum expression level: 1;; minimum fold change: 2.0;; p--value (adjusted)<0.01.

25	CEACAM6	-8.230129464	5.64E-56
26	EHF	-8.225310688	2.87E-186
27	PLG	-8.20252639	1.90E-11
28	FUT3	-8.18078281	6.75E-32
29	TM4SF4	-8.175474303	9.81E-22
30	B3GNT3	-8.11574604	2.78E-101
31	ESRP1	-8.101290874	3.95E-99
32	CST4	-8.056017988	1.77E-05
33	APCS	-8.030060286	7.98E-18
34	FGF19	-8.023024939	1.51E-08
35	F2	-8.017065479	4.75E-14
36	LOC100129520	-7.978152958	2.51E-07
37	TMPRSS2	-7.948639708	1.16E-26
38	HABP2	-7.929458589	2.96E-20
39	TSPAN8	-7.925318896	5.03E-19
40	PKHD1	-7.909438534	8.14E-142
41	ELF3	-7.859088143	1.70E-49
42	CREB3L3	-7.839149679	2.45E-09
43	ALB	-7.751952191	8.00E-38
44	TCN1	-7.691642408	2.16E-26
45	CYP2C18	-7.691104713	1.05E-18
46	APOB	-7.65088606	2.08E-25
47	MGST1	-7.621103935	2.93E-33
48	C19orf21	-7.583677509	1.77E-44
49	ORM2	-7.579356188	3.66E-12
50	SFTA2	-7.521280826	1.87E-13
51	C8A	-7.499830728	1.06E-12
52	SLC6A14	-7.499084583	4.44E-16

DEGs between the sarc/carc tumor component are shown in (A) and between the met/carc tumor component in (B) arranged by log2 fold change. Criteria for selection DEG: minimum expression level: 1;; minimum fold change: 2.0;; p--value (adjusted)<0.01.

53	KLK11	-7.493057096	1.77E-38
54	TSPAN1	-7.43149956	1.18E-42
55	MMP7	-7.395485906	5.92E-115
56	SERPINB5	-7.392567842	5.51E-73
57	ITIH1	-7.391462421	5.71E-10
58	TACSTD2	-7.388881471	2.56E-108
59	ARL14	-7.384756889	3.66E-12
60	CRP	-7.381922928	3.65E-14
61	ORM1	-7.371855095	4.76E-06
62	HP	-7.370124969	4.09E-34
63	GRHL2	-7.301913496	6.94E-20
64	FAM83A	-7.292664821	2.65E-22
65	HSD17B2	-7.285250685	1.07E-22
66	MBL2	-7.280448577	1.79E-11
67	FAM110C	-7.269963317	4.85E-35
68	EPCAM	-7.218897219	1.36E-93
69	HTR2C	-7.21460096	1.77E-11
70	TRIM29	-7.211975678	4.55E-08
71	KLK10	-7.202828586	3.04E-68
72	PDZK1IP1	-7.18245663	4.52E-12
73	FGL1	-7.152092003	8.07E-12
74	PPP1R14C	-7.145249072	1.95E-21
75	FXYD3	-7.112903473	2.86E-32
76	C4BPA	-7.104703161	7.71E-16
77	SLC5A1	-7.082511608	1.31E-14
78	PWRN2	-7.054073344	4.06E-05
79	CTSE	-7.046364565	7.08E-15
80	TOX3	-7.044428286	6.60E-22

DEGs between the sarc/carc tumor component are shown in (A) and between the met/carc tumor component in (B) arranged by log2 fold change. Criteria for selection DEG: minimum expression level: 1;; minimum fold change: 2.0;; p--value (adjusted)<0.01.

81	LOC100505967	-7.030589372	1.33E-09
82	DACT2	-6.997153249	1.13E-10
83	PCSK9	-6.995587651	9.21E-08
84	S100P	-6.993092797	2.86E-09
85	PIK3C2G	-6.975525375	1.42E-18
86	MYH14	-6.905256938	2.23E-75
87	SYT13	-6.887108281	8.22E-80
88	MAGEA1	-6.83806438	9.73E-11
89	CP	-6.831144951	1.86E-46
90	APOH	-6.826292366	6.02E-06
91	CBLC	-6.800824747	2.17E-25
92	CCDC135	-6.776896081	2.89E-05
93	CST6	-6.767532903	2.68E-05
94	CGN	-6.760065656	1.19E-39
95	C1orf116	-6.746545254	2.57E-33
96	PRR15	-6.693412034	1.97E-26
97	MUC5B	-6.679907633	1.42E-16
98	SYT8	-6.659429349	2.68E-05
99	VENTXP1	-6.648181501	1.44E-11
100	AGTR1	-6.626101828	2.41E-16
101	C1orf168	-6.619896607	4.37E-08
102	MAGEC1	-6.611216656	2.26E-09
103	C9	-6.576485981	4.77E-05
104	LOC201651	-6.551947179	2.99E-07
105	RMST	-6.546687785	9.50E-07
106	SCNN1A	-6.544214946	2.32E-26
107	RAB25	-6.529369094	2.21E-60
108	CYP4F3	-6.526037677	2.06E-12

DEGs between the sarc/carc tumor component are shown in (A) and between the met/carc tumor component in (B) arranged by log2 fold change. Criteria for selection DEG: minimum expression level: 1;; minimum fold change: 2.0;; p--value (adjusted)<0.01.

109	PLA2G4D	-6.524665679	0.001237632
110	FAM83E	-6.506423679	3.80E-15
111	UCA1	-6.494128052	0.001679502
112	GJB4	-6.466879785	3.11E-29
113	MAGEB18	-6.449187539	4.85E-05
114	EPPK1	-6.433223076	2.88E-42
115	OVOL1	-6.42189223	2.75E-11
116	PRSS22	-6.397167762	6.00E-36
117	DCAF8L2	-6.396639998	3.29712E-04
118	MAGEA12	-6.394902496	1.37E-05
119	MASP1	-6.38911352	1.92E-12
120	C9orf79	-6.388046098	0.001164087
121	ANO3	-6.364318137	8.61E-06
122	FGB	-6.362642392	2.28E-12
123	MTTP	-6.357519606	4.80E-11
124	DRD1	-6.34771567	3.39E-06
125	F7	-6.33971576	1.30E-05
126	FA2H	-6.317532252	8.06E-27
127	AGBL4	-6.317073132	4.62384E-04
128	CYP2C9	-6.316902902	9.49E-07
129	CFTR	-6.313189139	6.84E-07
130	LOC157627	-6.308861107	9.65E-05
131	UGT2A3	-6.298270339	1.20E-16
132	ITIH3	-6.297342176	6.55E-24
133	IGFBP1	-6.281860008	4.32E-18
134	CAPN13	-6.257861805	1.24E-09
135	ITIH2	-6.251931879	8.32E-13
136	HGD	-6.249439024	1.93E-19

DEGs between the sarc/carc tumor component are shown in (A) and between the met/carc tumor component in (B) arranged by log2 fold change. Criteria for selection DEG: minimum expression level: 1;; minimum fold change: 2.0;; p--value (adjusted)<0.01.

137	LOC100131726	-6.244563509	2.85E-08
138	FGFBP1	-6.237570217	0.00318499
139	PKD1L2	-6.191178302	2.40E-12
140	ENTPD2	-6.174732144	6.22E-15
141	RHOV	-6.117834962	9.72E-27
142	UGT2B4	-6.117685752	3.64E-09
143	OVOL2	-6.116510586	1.50899E-04
144	CRISP3	-6.092865679	2.00E-05
145	GCNT3	-6.075866781	6.09E-16
146	PON1	-6.070780738	5.76E-05
147	ANKRD56	-6.067377558	1.12E-21
148	SMEK3P	-6.052023513	2.89E-12
149	LOC100507651	-6.033088705	0.00309194
150	SLC17A4	-6.028906333	7.70E-07
151	CD24	-6.025086372	6.33E-82
152	ASGR2	-6.018282958	4.30E-13
153	AGT	-6.013212812	4.89E-13
154	FAM83B	-6.00977336	1.02E-23
155	PPP1R9A	-5.999484003	1.34E-62
156	CRB3	-5.998831151	1.27E-12
157	IVL	-5.962426487	1.11E-08
158	KLK8	-5.960000851	3.55E-09
159	PRSS8	-5.957137722	2.53E-58
160	SLC9A2	-5.957017294	2.49E-06
161	OIT3	-5.931879604	0.001562137
162	F11	-5.915714913	1.88331E-04
163	LOC100505583	-5.906631005	0.009449402
164	PGLYRP4	-5.89039603	0.002059093

DEGs between the sarc/carc tumor component are shown in (A) and between the met/carc tumor component in (B) arranged by log2 fold change. Criteria for selection DEG: minimum expression level: 1;; minimum fold change: 2.0;; p--value (adjusted)<0.01.

165	CYP4B1	-5.875626503	0.003386501
166	PCK1	-5.862741402	1.24E-19
167	SLC38A3	-5.862687891	0.002238954
168	CSAG1	-5.839904015	3.22038E-04
169	WNK2	-5.832258712	1.16E-36
170	ABCB11	-5.825326109	5.30E-15
171	A1CF	-5.818273745	6.32E-30
172	C4BPB	-5.802281707	1.12E-10
173	FOXI2	-5.754527221	0.004496194
174	AZGP1	-5.747418951	7.24E-15
175	KRT5	-5.74693501	0.001877233
176	COL9A3	-5.713964277	6.74E-51
177	MAT1A	-5.711285175	1.44E-09
178	S100A14	-5.710227827	4.17E-06
179	MSLN	-5.708422192	1.04E-05
180	KLK7	-5.704084421	6.70077E-04
181	C8orf51	-5.697081154	0.00164435
182	CYP4F12	-5.696196432	2.13E-10
183	FBN3	-5.691097766	1.96E-05
184	LOC286189	-5.685011745	0.005945479
185	HNF4A	-5.674465651	1.04E-30
186	C2orf72	-5.669705384	1.66E-07
187	NDNF	-5.664476043	9.57E-06
188	LOC642929	-5.655199122	0.004108106
189	KIAA1244	-5.65449177	1.88E-49
190	FAM47A	-5.646625063	0.00077657
191	MAGEB6	-5.646353102	0.002655145
192	PADI1	-5.640649632	2.82E-19

DEGs between the sarc/carc tumor component are shown in (A) and between the met/carc tumor component in (B) arranged by log2 fold change. Criteria for selection DEG: minimum expression level: 1;; minimum fold change: 2.0;; p--value (adjusted)<0.01.

193	HPN	-5.639316949	1.68E-15
194	AHSG	-5.632923535	0.003704041
195	POU4F2	-5.625342755	0.008098372
196	DMKN	-5.624671877	1.17E-33
197	SLC30A10	-5.623395249	4.73E-07
198	NR1I2	-5.617816426	1.10E-06
199	DCAF4L2	-5.612080924	7.07956E-04
200	SLC25A41	-5.602638609	0.005941023
201	OR5P3	-5.595837044	6.89682E-04
202	KRT16	-5.592380153	8.98E-26
203	WDR72	-5.586538435	6.30E-18
204	AADAC	-5.585105021	2.92E-09
205	C6orf15	-5.579224057	7.28172E-04
206	C3	-5.57094298	5.53E-07
207	MAPK4	-5.570758504	2.04195E-04
208	LOC154860	-5.558877515	8.43E-05
209	ITGB4	-5.550071767	1.40E-25
210	KRT23	-5.531416837	9.18E-23
211	BK250D10.8	-5.528147881	0.009227201
212	IL8	-5.519121945	2.07E-12
213	AAA1	-5.509167762	1.26E-09
214	ARHGEF38	-5.506279469	1.73E-27
215	MAGEA8	-5.499555242	2.02838E-04
216	CHST4	-5.497979288	1.09E-13
217	TJP3	-5.495703945	4.52E-32
218	GABRA4	-5.489804756	8.08865E-04
219	KIR2DL1	-5.486249016	0.00732945
220	UGT2B7	-5.478919418	6.72E-09

DEGs between the sarc/carc tumor component are shown in (A) and between the met/carc tumor component in (B) arranged by log2 fold change. Criteria for selection DEG: minimum expression level: 1;; minimum fold change: 2.0;; p--value (adjusted)<0.01.

221	TNS4	-5.473615894	3.25201E-04
222	LOC400940	-5.471586809	1.79E-05
223	RAP1GAP	-5.467257057	1.06E-60
224	DMBT1	-5.465610659	8.21E-10
225	CRABP2	-5.465057407	2.20E-18
226	GPR50	-5.458427329	1.03E-05
227	MACC1	-5.442962033	4.68E-136
228	MGC34034	-5.435764988	0.004496245
229	PIP	-5.411072326	0.003722428
230	CCDC144NL	-5.40624936	0.004839008
231	C10orf81	-5.403792929	9.67E-21
232	C20orf151	-5.401278017	5.03E-06
233	LGALS4	-5.399045895	1.98E-05
234	ERN2	-5.397363601	1.75E-15
235	CNGA4	-5.384011255	0.006079012
236	ST8SIA6	-5.374794588	0.007040862
237	KRT4	-5.374283348	1.35E-05
238	SSX8	-5.3707808	0.007690577
239	TESC	-5.338444206	3.57E-40
240	CDH3	-5.324461692	1.48E-40
241	ABCC11	-5.305415113	0.008969882
242	TF	-5.304807549	5.26E-22
243	SEC14L6	-5.283939756	0.008724406
244	WSCD2	-5.255152938	5.56E-05
245	SPINK1	-5.246680802	5.27E-16
246	F9	-5.243680922	3.84605E-04
247	POF1B	-5.242912117	2.37E-29
248	CYP2J2	-5.22660868	2.64E-07

DEGs between the sarc/carc tumor component are shown in (A) and between the met/carc tumor component in (B) arranged by log2 fold change. Criteria for selection DEG: minimum expression level: 1;; minimum fold change: 2.0;; p--value (adjusted)<0.01.

249	HPX	-5.226605852	1.48049E-04
250	SCEL	-5.21774309	3.29E-80
251	GSTA7P	-5.21073422	4.07E-05
252	MPZL2	-5.21004312	2.33E-70
253	PHF2P1	-5.208561344	6.49E-06
254	USH1C	-5.208355609	3.66E-06
255	TRIM15	-5.205495957	2.42E-07
256	CLDN7	-5.203306901	1.63E-50
257	KRT6A	-5.199069569	2.27E-06
258	SAA1	-5.189399023	0.001689206
259	SHANK2	-5.180690383	1.12E-18
260	LOC339685	-5.17877808	0.005883596
261	BSPRY	-5.156159373	3.79E-34
262	BARX2	-5.155746542	1.79E-19
263	AIM1L	-5.154629233	5.01E-37
264	NR1H4	-5.148260083	3.15E-09
265	CPB2	-5.148205205	4.77E-07
266	TMEM125	-5.143016552	2.13E-12
267	C19orf59	-5.136888435	6.84E-07
268	LOC442459	-5.122154638	1.66913E-04
269	LYPD6	-5.122012621	2.57045E-04
270	SCIN	-5.107129167	2.00E-10
271	GREM2	-5.099061578	1.63E-13
272	ALDOB	-5.087974135	4.15E-05
273	TM4SF5	-5.082805937	1.21E-06
274	KRT7	-5.082641651	6.96E-27
275	UGT2B11	-5.067707556	0.007714051
276	MYEOV	-5.066478054	1.04E-40

DEGs between the sarc/carc tumor component are shown in (A) and between the met/carc tumor component in (B) arranged by log2 fold change. Criteria for selection DEG: minimum expression level: 1;; minimum fold change: 2.0;; p--value (adjusted)<0.01.

277	FRAS1	-5.066030828	4.67E-23
278	KRT13	-5.065807887	1.94E-08
279	C8G	-5.063431458	1.94559E-04
280	EPS8L3	-5.060060219	6.60E-24
281	SFTA1P	-5.052610171	1.56E-05
282	FTCD	-5.047470154	4.89E-07
283	NIPAL1	-5.047307315	4.61E-17
284	ZG16B	-5.045036339	7.07E-08
285	FAM180A	-5.033689871	6.00E-06
286	ERBB3	-5.017730827	6.33E-82
287	TRIM31	-5.014055941	4.60495E-04
288	GPR35	-5.008907444	6.90E-42
289	ALDH3B2	-5.008245515	5.94288E-04
290	IGSF1	-5.003805751	3.20693E-04
291	AGXT2	-4.995208904	0.003127337
292	CACNG4	-4.991187583	3.08E-09
293	PLA2G4F	-4.987839431	1.42E-05
294	IRF6	-4.985916725	2.08E-82
295	FIBCD1	-4.979171015	6.34E-48
296	FAM160A1	-4.976589964	1.13E-41
297	PSCA	-4.963625301	1.91E-06
298	EREG	-4.957169429	3.65E-75
299	LMO3	-4.951725827	4.28E-11
300	DPYS	-4.950713921	2.00596E-04
301	TFPI2	-4.947130716	4.71E-11
302	STK32A	-4.942269054	2.53E-07
303	SPAM1	-4.936609546	0.005918395
304	ZNF541	-4.93505822	0.004176746

DEGs between the sarc/carc tumor component are shown in (A) and between the met/carc tumor component in (B) arranged by log2 fold change. Criteria for selection DEG: minimum expression level: 1;; minimum fold change: 2.0;; p--value (adjusted)<0.01.

305	GSG1L	-4.927554903	2.17E-09
306	OVCH2	-4.926390898	2.65438E-04
307	GOLT1A	-4.917943942	1.98E-10
308	SORCS3	-4.897696399	0.00952516
309	HMGB3P1	-4.878366388	0.009482406
310	MAOA	-4.876607722	4.75E-12
311	RDH8	-4.865938086	0.002212527
312	KRT17	-4.858502686	2.31E-44
313	WNT7A	-4.856810349	1.45E-25
314	AQP2	-4.856207737	0.006793271
315	MAGEC2	-4.85228426	0.002212527
316	ACSM1	-4.84398691	5.96E-05
317	AADACL3	-4.841002807	0.002079778
318	ZIC4	-4.834913135	2.84E-05
319	PCDH20	-4.827410463	0.001510994
320	NRAP	-4.814764057	0.005100009
321	ZP4	-4.814464816	0.00702203
322	MB	-4.811537606	5.24531E-04
323	AGR3	-4.811373894	7.77767E-04
324	CYP4F11	-4.803383926	3.72E-18
325	CLDN6	-4.80308428	2.39E-05
326	FREM2	-4.802293972	2.50E-09
327	GEMC1	-4.786890441	0.001700964
328	SLC22A12	-4.785756647	0.0025299
329	C10orf71	-4.783522859	0.003707926
330	AGXT2L1	-4.77470334	0.001656682
331	TEX15	-4.769093581	0.008912824
332	TMEM30B	-4.760894394	8.06E-42

DEGs between the sarc/carc tumor component are shown in (A) and between the met/carc tumor component in (B) arranged by log2 fold change. Criteria for selection DEG: minimum expression level: 1;; minimum fold change: 2.0;; p--value (adjusted)<0.01.

333	C8orf47	-4.757457135	7.34E-05
334	TMEM139	-4.744393851	6.62E-28
335	SGPP2	-4.741624199	7.51E-15
336	NBPF22P	-4.74153672	0.001842236
337	C8orf75	-4.737249711	0.006427715
338	SAA2	-4.734411018	1.98E-05
339	CPA6	-4.730242908	0.001268499
340	ISM2	-4.727183894	3.73865E-04
341	CYP4F2	-4.725076574	0.008531842
342	CYP26A1	-4.71309759	0.00194512
343	SULT2B1	-4.711900505	1.28E-05
344	MAGEB1	-4.699409143	0.003111431
345	GABARAPL3	-4.696630416	0.006815317
346	PAK6	-4.692504487	9.39E-15
347	TMEM40	-4.685662847	0.004577294
348	SPINT1	-4.681980042	1.03E-30
349	F5	-4.681604056	7.73E-85
350	GPR144	-4.681379627	0.005777519
351	KRT8	-4.672305838	2.35E-35
352	DKFZp451B082	-4.669883737	0.001844497
353	CLDN3	-4.669392862	7.97E-12
354	SLC5A8	-4.669282041	2.11287E-04
355	SERPINF2	-4.656274787	9.37E-12
356	CRNN	-4.65504267	0.007853857
357	MYO5C	-4.651347457	1.33E-43
358	SLC5A12	-4.650229755	2.82E-09
359	AMDHD1	-4.648783271	3.81231E-04
360	ESPN	-4.648427999	2.35E-09

DEGs between the sarc/carc tumor component are shown in (A) and between the met/carc tumor component in (B) arranged by log2 fold change. Criteria for selection DEG: minimum expression level: 1;; minimum fold change: 2.0;; p--value (adjusted)<0.01.

361	TGFA	-4.647177555	1.75E-70
362	PADI4	-4.645948667	0.007938435
363	GPR26	-4.642351428	6.66E-05
364	CES1	-4.633708148	1.09E-10
365	VNN1	-4.628634445	2.70E-40
366	SEMA3E	-4.628468258	2.25E-27
367	EPS8L1	-4.622675482	1.01E-23
368	TNR	-4.614758392	0.005359178
369	SPINT2	-4.601590054	6.83E-51
370	TNFRSF10C	-4.598133882	2.20E-08
371	DRD2	-4.593111967	0.00569369
372	CCDC64B	-4.587395363	4.07E-06
373	FLJ46257	-4.586198218	0.002063041
374	RND1	-4.581650811	8.17E-05
375	RBM20	-4.569215445	3.47839E-04
376	CDS1	-4.568782492	1.38E-43
377	TCL6	-4.568383317	5.32E-05
378	HPGD	-4.563241396	1.28E-47
379	DPP4	-4.559523116	4.39E-38
380	EPN3	-4.556305037	1.55E-14
381	ABCA12	-4.554249087	2.03E-10
382	IL36RN	-4.553146797	0.006003512
383	BCMO1	-4.546662109	3.41E-06
384	KIAA1543	-4.537226932	4.33E-20
385	LRP1B	-4.534899092	1.42E-05
386	DKK1	-4.526965254	3.37E-05
387	MMP15	-4.526364178	3.42E-44
388	MIA2	-4.520989294	1.33E-24

DEGs between the sarc/carc tumor component are shown in (A) and between the met/carc tumor component in (B) arranged by log2 fold change. Criteria for selection DEG: minimum expression level: 1;; minimum fold change: 2.0;; p--value (adjusted)<0.01.

389	KCNK1	-4.516184379	1.37E-23
390	INHBC	-4.516002631	0.001745363
391	AKR7A3	-4.512860467	6.47E-08
392	LOC100130238	-4.511643841	4.24882E-04
393	C11orf87	-4.506119377	0.008277862
394	CNGA1	-4.504171441	3.55E-11
395	GALNT3	-4.50289402	6.90E-42
396	PKP1	-4.495185176	4.23E-13
397	CACNG3	-4.491821648	4.80315E-04
398	CEACAM1	-4.489744047	1.23E-47
399	KCNH5	-4.47776198	0.006447385
400	FAM169B	-4.473269226	6.17E-08
401	CCL16	-4.470113779	0.001199306
402	VTN	-4.465441289	0.002252352
403	PCDHA12	-4.461022144	7.92E-05
404	MUC17	-4.445637778	0.001946288
405	CCDC155	-4.445009239	0.00167993
406	CNTN5	-4.437716465	7.76E-05
407	PROM2	-4.434891354	1.14E-26
408	PLA2G5	-4.432207613	1.27455E-04
409	HOXA11	-4.410912343	0.009199526
410	ATP6V0A4	-4.404954253	0.001835226
411	CPN1	-4.39801202	0.00913033
412	ABCG8	-4.39717296	3.64E-05
413	PROL1	-4.395542739	0.001216931
414	PKD2L1	-4.395507247	0.006792322
415	ABCC8	-4.392230639	0.003334827
416	RACGAP1P	-4.387766375	0.002652819

DEGs between the sarc/carc tumor component are shown in (A) and between the met/carc tumor component in (B) arranged by log2 fold change. Criteria for selection DEG: minimum expression level: 1;; minimum fold change: 2.0;; p--value (adjusted)<0.01.

417	SLITRK3	-4.381856431	0.004523762
418	SGK2	-4.380789798	6.76E-08
419	CDH19	-4.378714929	1.89726E-04
420	RORC	-4.377032464	7.10E-07
421	LOC340515	-4.376367201	0.003508353
422	MAG	-4.372160979	0.00403761
423	LOC339505	-4.369160945	8.98764E-04
424	FOXA1	-4.368973228	6.37E-07
425	FAM75D1	-4.366773557	1.66798E-04
426	IL1RAPL2	-4.364114492	0.001149913
427	LOC348120	-4.361500644	0.003720409
428	LIMCH1	-4.361132897	1.82E-29
429	CYP24A1	-4.359374546	4.24E-10
430	PRDM9	-4.359196035	0.007070519
431	CTSL2	-4.357049871	5.03E-11
432	STOX1	-4.354547138	0.00403761
433	PPARGC1A	-4.35003755	2.07E-10
434	GJB3	-4.347522922	6.56E-17
435	KLHL31	-4.343800828	4.71527E-04
436	NRG3	-4.337724244	4.29E-07
437	AMBP	-4.33717703	1.90146E-04
438	CA10	-4.33533545	0.002216534
439	ANKRD1	-4.33301608	2.44E-20
440	FLJ42289	-4.328981265	0.009808065
441	GPR179	-4.327260241	0.002187591
442	PAH	-4.325029289	2.26E-05
443	GJB6	-4.32391503	1.06E-06
444	C1orf111	-4.319004094	0.003897891

DEGs between the sarc/carc tumor component are shown in (A) and between the met/carc tumor component in (B) arranged by log2 fold change. Criteria for selection DEG: minimum expression level: 1;; minimum fold change: 2.0;; p--value (adjusted)<0.01.

445	ITIH4	-4.317999835	1.14E-08
446	C7orf52	-4.30878619	0.008357332
447	COL17A1	-4.303263341	8.54E-14
448	DUOX2	-4.302683964	1.22E-19
449	PLS1	-4.291898243	8.81E-23
450	MAOB	-4.290051965	2.10E-35
451	EN2	-4.28655961	6.61886E-04
452	TNFRSF11B	-4.286056668	1.83E-07
453	HOTAIR	-4.285620415	3.69329E-04
454	CRLF1	-4.284136503	1.94E-08
455	RGL3	-4.279781921	4.06E-23
456	LIPC	-4.276011049	2.21E-08
457	ESRP2	-4.274529231	1.02E-39
458	CHDH	-4.273164472	4.36E-40
459	C9orf29	-4.272667129	0.002744613
460	VWA2	-4.270626033	1.27E-17
461	FSTL5	-4.270062267	7.74E-06
462	LHX9	-4.26729745	0.00045644
463	ADH6	-4.25764978	5.57194E-04
464	MUC1	-4.246103193	8.09E-73
465	C20orf54	-4.234306518	3.51E-10
466	KCP	-4.230686439	4.22E-21
467	FGF12	-4.228159897	7.42E-07
468	STEAP4	-4.224720607	6.38E-25
469	SYBU	-4.222254733	1.45E-08
470	CCL28	-4.216802226	8.73291E-04
471	PATE4	-4.209012171	0.003781327
472	AKR1C3	-4.207411685	2.69E-10

DEGs between the sarc/carc tumor component are shown in (A) and between the met/carc tumor component in (B) arranged by log2 fold change. Criteria for selection DEG: minimum expression level: 1;; minimum fold change: 2.0;; p--value (adjusted)<0.01.

473	ADRA2B	-4.20728702	0.005345966
474	AIF1L	-4.206266017	6.09133E-04
475	EMR3	-4.195731053	0.004325121
476	OGDHL	-4.194521658	0.003119506
477	LOC442497	-4.194290356	0.002860872
478	PTPRZ1	-4.192424681	7.91E-10
479	SPDEF	-4.192387289	1.28E-05
480	HCAR2	-4.191060774	0.004438055
481	MAP7	-4.190849479	1.48E-35
482	CREG2	-4.184051629	2.15E-06
483	TDRD1	-4.181700162	9.80749E-04
484	TRPV6	-4.17553361	1.75E-09
485	GPR112	-4.173028058	0.001188138
486	RNF43	-4.172407688	9.49E-20
487	TFR2	-4.15627725	6.32E-13
488	FAM83F	-4.154781476	9.69E-11
489	DPP10	-4.1528409	0.008373211
490	CYP3A4	-4.148680234	0.0006021
491	LRG1	-4.14658403	3.82E-30
492	HOXC13	-4.143319467	3.12355E-04
493	NPC1L1	-4.143033837	5.54E-08
494	GRIK3	-4.139909584	0.001921764
495	GPA33	-4.138283656	0.001775055
496	OR5E1P	-4.137445949	0.003051905
497	HAO2	-4.136505427	0.003180533
498	NEFL	-4.117612888	0.001901128
499	SOX2	-4.117542259	5.20E-07
500	BTBD16	-4.117465589	0.009846547

DEGs between the sarc/carc tumor component are shown in (A) and between the met/carc tumor component in (B) arranged by log2 fold change. Criteria for selection DEG: minimum expression level: 1;; minimum fold change: 2.0;; p--value (adjusted)<0.01.

501	C6	-4.113840376	5.38E-11
502	PRG4	-4.11356028	7.22E-12
503	OR7E91P	-4.110839329	0.009820041
504	LPO	-4.108989388	0.003370792
505	MYOM2	-4.10810803	0.005560982
506	CCDC164	-4.101291755	0.007578863
507	P2RY2	-4.100609278	1.15E-29
508	CLIC5	-4.096044873	9.23E-25
509	CHD5	-4.082140826	2.09871E-04
510	ARSF	-4.081696041	0.003292265
511	SH3BGRL2	-4.076574125	4.17E-16
512	DSP	-4.074250214	3.15E-61
513	TMPRSS13	-4.073766001	1.81E-07
514	SLITRK1	-4.068349365	2.34E-06
515	STYK1	-4.065993207	3.58E-17
516	SLC22A20	-4.057523221	0.001045144
517	SH2D4B	-4.049818232	0.001495526
518	LLGL2	-4.047216748	5.94E-18
519	CACNG2	-4.042685427	0.007722012
520	TTC22	-4.042625187	7.73E-11
521	CEACAM5	-4.041789902	2.84E-07
522	SYT6	-4.035840287	0.008004314
523	TMC4	-4.03263889	4.07E-28
524	HOOK1	-4.032470876	2.60E-37
525	GC	-4.020968587	9.98E-09
526	PNMA5	-4.007511921	0.007690577
527	TTPA	-4.003253446	0.005081412
528	KRT18	-4.001580461	3.92E-12

DEGs between the sarc/carc tumor component are shown in (A) and between the met/carc tumor component in (B) arranged by log2 fold change. Criteria for selection DEG: minimum expression level: 1;; minimum fold change: 2.0;; p--value (adjusted)<0.01.

529	CLDN2	-4.001176338	1.63084E-04
530	TFCP2L1	-3.99964151	5.71E-44
531	FLJ45974	-3.998705741	2.30E-05
532	TUBBP5	-3.997218093	2.60E-05
533	CALB2	-3.99422774	3.63E-19
534	ANP32C	-3.993822441	0.004203595
535	PRKAA2	-3.990097387	1.16E-25
536	GRHL1	-3.988951358	6.87E-25
537	ACSM5	-3.987878057	2.81E-05
538	KCNH8	-3.987520687	3.03E-08
539	ST14	-3.982649472	7.14E-24
540	HPD	-3.978664572	2.83E-06
541	BTC	-3.976259159	3.61503E-04
542	SPTSSB	-3.969788153	7.62E-07
543	SP8	-3.963601208	0.003232687
544	DPCR1	-3.955201076	0.001717865
545	CSMD1	-3.952153735	0.00629977
546	VIPR1	-3.949110082	3.44E-05
547	TMC5	-3.948775588	5.69E-12
548	SPTA1	-3.948136767	0.005917381
549	EPHB6	-3.94700962	1.14E-29
550	RPH3A	-3.939264566	0.007551765
551	MUC7	-3.938289786	6.72E-06
552	COL2A1	-3.926620954	0.008979363
553	KNG1	-3.914754942	1.94E-05
554	TRDN	-3.914377684	5.07363E-04
555	ETV3L	-3.914005414	4.22531E-04
556	LSR	-3.904643591	9.55E-14

DEGs between the sarc/carc tumor component are shown in (A) and between the met/carc tumor component in (B) arranged by log2 fold change. Criteria for selection DEG: minimum expression level: 1;; minimum fold change: 2.0;; p--value (adjusted)<0.01.

557	VIL1	-3.904261335	2.69E-06
558	LAMB3	-3.902675494	2.54E-68
559	ACSM2A	-3.900656237	4.07E-06
560	C6orf223	-3.89511177	0.001656682
561	C12orf67	-3.890097802	0.001280347
562	PFKFB1	-3.886382134	0.00047064
563	BCHE	-3.884677834	7.69311E-04
564	C1orf173	-3.882498692	9.29733E-04
565	EDAR	-3.872794436	0.003978518
566	CDH8	-3.872136129	0.006952914
567	CASP14	-3.871925305	5.09526E-04
568	LOC100131320	-3.870865967	0.006318512
569	C9orf170	-3.869159157	0.009016277
570	KRT1	-3.859482556	0.001502562
571	RBP4	-3.858095394	0.008076481
572	CUX2	-3.852633708	8.66E-07
573	SCN4A	-3.851775413	0.004516945
574	FCN3	-3.850519722	0.007593218
575	G6PC	-3.84936349	7.34E-05
576	HKDC1	-3.847706902	3.56E-16
577	KLHL30	-3.847164359	0.001246144
578	CHAT	-3.846202823	0.005898204
579	C2orf71	-3.846158176	0.005751732
580	LOC100133311	-3.841652852	0.002831509
581	MT1X	-3.838902028	1.07E-12
582	INPP4B	-3.832243697	5.38E-28
583	LOC388564	-3.830390163	0.002675487
584	BCAS1	-3.827624552	1.69E-14

DEGs between the sarc/carc tumor component are shown in (A) and between the met/carc tumor component in (B) arranged by log2 fold change. Criteria for selection DEG: minimum expression level: 1;; minimum fold change: 2.0;; p--value (adjusted)<0.01.

585	CX3CL1	-3.827141516	1.79E-16
586	GATA4	-3.825398482	2.55E-05
587	OTOL1	-3.824764083	0.004397971
588	ALS2CL	-3.820618363	1.13E-24
589	PAX9	-3.809508356	7.17E-08
590	ATG9B	-3.804891128	1.82E-07
591	RAB17	-3.804822425	2.19E-08
592	PNPLA3	-3.804298262	3.56E-15
593	CDC42BPG	-3.803120781	1.75E-26
594	ARMC3	-3.80105854	7.35723E-04
595	RASAL1	-3.800894676	7.49E-13
596	WFDC1	-3.800783607	6.21E-08
597	ATP1A4	-3.798624228	2.34E-06
598	GCK	-3.795903806	0.005809957
599	BIK	-3.791662072	8.58E-12
600	SHISA2	-3.78956015	1.94E-29
601	ABCC6P1	-3.78634768	0.001582057
602	ALDH1L1	-3.784230573	2.51E-06
603	LOC284233	-3.780115904	1.02E-09
604	FAM155B	-3.773281696	0.001372096
605	CNGB1	-3.770752826	0.006306792
606	CAPN6	-3.760204435	3.40782E-04
607	PDE9A	-3.757425613	1.20E-13
608	DIO3	-3.757085846	0.001698067
609	ITIH5L	-3.745090387	0.003621938
610	SPRNP1	-3.743881119	4.48E-06
611	ICA1	-3.738147867	2.46E-26
612	KCNH4	-3.737905658	2.12E-05

DEGs between the sarc/carc tumor component are shown in (A) and between the met/carc tumor component in (B) arranged by log2 fold change. Criteria for selection DEG: minimum expression level: 1;; minimum fold change: 2.0;; p--value (adjusted)<0.01.

613	POU6F2	-3.73762031	6.61E-06
614	GREB1L	-3.73540919	5.39E-26
615	BAAT	-3.73396655	6.46E-06
616	SLC25A21	-3.733080223	1.55E-05
617	BCRP3	-3.73289276	0.003389134
618	ELOVL7	-3.7264857	1.05E-09
619	TBX5	-3.723774308	9.64918E-04
620	ZAN	-3.720499279	2.41842E-04
621	UGT3A1	-3.716260668	0.00250927
622	CD1C	-3.711661345	6.11E-05
623	EYA4	-3.710247237	5.94E-05
624	IGLON5	-3.709043625	3.81E-05
625	psiTPTE22	-3.707885172	9.69E-06
626	MYO3A	-3.70456171	2.67453E-04
627	SLC28A3	-3.703741563	6.74E-09
628	ANKRD30A	-3.700441339	0.001191713
629	SLC14A1	-3.698753868	2.03E-19
630	CCDC11	-3.688040243	0.008193603
631	DSCAM-AS1	-3.683987805	0.007244752
632	FAM83C	-3.681495391	0.00024895
633	ANO5	-3.681090792	2.31E-06
634	HHIP	-3.679382371	1.40E-09
635	MUC12	-3.677551466	0.004516945
636	MUC4	-3.674633615	3.23E-07
637	C3P1	-3.672820641	5.27E-05
638	TM4SF18	-3.66720438	1.67E-27
639	FAM75C1	-3.6636289	0.007750948
640	IYD	-3.657619543	0.003664228

DEGs between the sarc/carc tumor component are shown in (A) and between the met/carc tumor component in (B) arranged by log2 fold change. Criteria for selection DEG: minimum expression level: 1;; minimum fold change: 2.0;; p--value (adjusted)<0.01.

641	CYP4X1	-3.654482542	6.26E-13
642	C1orf226	-3.651611336	1.58E-08
643	SP7	-3.646273784	1.91432E-04
644	ART4	-3.6450101	5.79179E-04
645	HAL	-3.643832269	5.31E-07
646	PDX1	-3.642244264	8.98E-06
647	KCND3	-3.639597285	0.004466013
648	SLC15A1	-3.636147143	2.95E-42
649	DPP6	-3.635013986	0.004523762
650	BAIAP2L2	-3.634145719	1.51E-08
651	EDN1	-3.633569853	3.52E-13
652	GYLTL1B	-3.63320109	5.48353E-04
653	MNX1	-3.629899789	2.75E-05
654	C1orf172	-3.627406981	6.63E-07
655	ZNF804B	-3.624315835	0.007803693
656	ABCA13	-3.620048399	3.12719E-04
657	CPN2	-3.615817285	0.001816123
658	IL17RE	-3.596228126	4.04E-19
659	VSX2	-3.593514361	0.006989578
660	FAM183B	-3.588253783	2.01782E-04
661	PLEKHA6	-3.587330375	2.36E-32
662	LOC100131551	-3.58332278	4.69421E-04
663	INHBE	-3.58317094	6.33E-06
664	RSPO4	-3.577629801	0.009420472
665	UGT2B15	-3.575275747	3.28E-05
666	EMX2	-3.574930052	4.45E-05
667	SH3TC2	-3.574837312	1.63E-37
668	MUM1L1	-3.57213672	0.001811564

DEGs between the sarc/carc tumor component are shown in (A) and between the met/carc tumor component in (B) arranged by log2 fold change. Criteria for selection DEG: minimum expression level: 1;; minimum fold change: 2.0;; p--value (adjusted)<0.01.

669	NEFM	-3.566855819	0.0007318
670	GPR133	-3.560231071	1.22081E-04
671	ENPP5	-3.55416733	1.55E-08
672	LOC100130705	-3.54697018	2.06E-09
673	ABCG5	-3.54030268	0.009956218
674	PCDH15	-3.535358563	3.16305E-04
675	HRASLS5	-3.527216301	0.001351535
676	PIGR	-3.526486709	2.06E-07
677	C1orf115	-3.524886513	3.59E-17
678	FAAH	-3.520160435	2.91E-05
679	GPR143	-3.519851864	5.11801E-04
680	TCEAL2	-3.513957123	3.85518E-04
681	EPHX3	-3.509567338	1.36E-07
682	HAO1	-3.506185843	4.34471E-04
683	LAMA3	-3.50357252	5.97E-34
684	SLCO4C1	-3.502997172	1.57E-05
685	DUSP27	-3.490407069	0.001037569
686	SLC1A1	-3.490289104	5.03E-19
687	DIO3OS	-3.48908374	0.00185849
688	GPR56	-3.482144224	8.21E-53
689	SLC13A3	-3.478023895	2.92E-05
690	PPL	-3.476925398	6.84E-08
691	KLHDC7A	-3.47352573	1.77E-08
692	AKR7L	-3.472243733	1.98E-05
693	KIF1A	-3.471564343	9.19591E-04
694	CYP39A1	-3.468312407	0.005647842
695	MST1R	-3.462191136	2.71E-34
696	EVPL	-3.456105541	2.06E-09

DEGs between the sarc/carc tumor component are shown in (A) and between the met/carc tumor component in (B) arranged by log2 fold change. Criteria for selection DEG: minimum expression level: 1;; minimum fold change: 2.0;; p--value (adjusted)<0.01.

697	MYT1L	-3.447441608	0.00705014
698	SLC12A3	-3.446048135	0.00719012
699	CXCL2	-3.440653164	8.54E-16
700	KCNQ4	-3.439481376	6.57E-06
701	MPZL3	-3.438207622	5.39E-09
702	KLKB1	-3.435832197	0.001943687
703	ADAP1	-3.433978154	1.01E-10
704	MGAM	-3.432536742	0.007551246
705	ILDR1	-3.430808791	4.90E-07
706	CRISPLD1	-3.43073772	3.99E-05
707	VAX1	-3.428411411	0.00269811
708	FGFR2	-3.425837004	1.10E-06
709	NXF4	-3.423761885	3.76024E-04
710	PRSS16	-3.416748102	5.31069E-04
711	C9orf125	-3.416556419	1.70E-06
712	RTN4R	-3.416066867	2.47033E-04
713	WNT10A	-3.397780032	1.52E-07
714	SLC24A2	-3.393823286	0.005039822
715	PLEKHB1	-3.388806196	7.16E-11
716	ONECUT1	-3.387652955	1.71E-06
717	NOS1AP	-3.386948744	2.12E-06
718	NPTX1	-3.386741736	4.90997E-04
719	EPHX4	-3.385511039	1.26E-05
720	NXPH4	-3.378243291	3.03449E-04
721	LGR6	-3.376922437	8.04376E-04
722	PHACTR3	-3.370759959	1.34529E-04
723	MLXIPL	-3.369806886	2.91E-08
724	VWA3A	-3.366537932	0.00541587

DEGs between the sarc/carc tumor component are shown in (A) and between the met/carc tumor component in (B) arranged by log2 fold change. Criteria for selection DEG: minimum expression level: 1;; minimum fold change: 2.0;; p--value (adjusted)<0.01.

725	GPR126	-3.362949435	1.71E-27
726	GABRA2	-3.357394839	0.004383347
727	PCLO	-3.356288278	1.56E-12
728	MAP7D2	-3.34778401	0.00342247
729	VSIG10L	-3.344230915	0.00013002
730	SERINC2	-3.343449342	1.75E-39
731	KSR2	-3.342567253	0.009767155
732	RP1L1	-3.342409515	0.004648341
733	LYPD4	-3.330980386	8.78957E-04
734	ANKRD18A	-3.324179347	3.57E-14
735	ATP2B3	-3.315482621	0.009129936
736	ADAMTS20	-3.314763739	0.0066419
737	SEMA3G	-3.312528263	0.003520909
738	CECR2	-3.31092759	6.29E-06
739	IGFBP3	-3.292108906	1.65E-29
740	SLC19A3	-3.273902428	2.47784E-04
741	PAX1	-3.267858061	0.00366092
742	TP63	-3.267311022	6.08309E-04
743	UNC13D	-3.263491187	8.97E-16
744	VWA1	-3.263137131	1.05E-07
745	LOC645323	-3.259408799	0.004186441
746	PRR15L	-3.257867874	3.68E-09
747	NEURL3	-3.256957314	1.96E-05
748	LOC100130899	-3.253896323	3.03E-05
749	CNNM1	-3.24651164	5.75E-09
750	FAM174B	-3.24589585	1.20E-16
751	CX3CR1	-3.243904495	0.008464029
752	FGFR3	-3.239176372	1.28E-10

DEGs between the sarc/carc tumor component are shown in (A) and between the met/carc tumor component in (B) arranged by log2 fold change. Criteria for selection DEG: minimum expression level: 1;; minimum fold change: 2.0;; p--value (adjusted)<0.01.

753	CLDN9	-3.229643143	0.009524775
754	RD3	-3.229594412	0.002403489
755	PLAT	-3.226157716	3.46E-09
756	SDR42E1	-3.224752446	8.51E-06
757	SLC1A2	-3.215670485	3.93E-08
758	SLC7A11	-3.215644433	4.76E-12
759	TPRX1	-3.212157104	0.009126974
760	ATP2B2	-3.20782785	0.005998837
761	LOC641364	-3.205037577	2.32E-14
762	LOC643623	-3.203716124	0.006160836
763	PKP2	-3.197396673	3.31E-26
764	LOC147646	-3.196949104	0.002022669
765	UNC5CL	-3.19641256	1.23E-08
766	IGF2BP1	-3.195012753	3.34E-06
767	LMOD2	-3.185445922	0.007811522
768	SOX13	-3.178760176	3.73E-26
769	ESRRG	-3.178337512	2.18458E-04
770	SLC9A7P1	-3.175344852	5.27659E-04
771	LOC100128682	-3.171575765	0.008451622
772	PALM3	-3.169503166	2.41E-07
773	SPINK5	-3.160478141	3.59E-17
774	G0S2	-3.156703144	4.98E-13
775	LOC283432	-3.154292052	0.003896641
776	DHRS9	-3.146337868	9.90E-07
777	LEMD1	-3.145784568	2.44E-06
778	MUC2	-3.144118334	1.41368E-04
779	C1orf130	-3.138396586	7.80E-07
780	STAP2	-3.137980872	1.75E-17

DEGs between the sarc/carc tumor component are shown in (A) and between the met/carc tumor component in (B) arranged by log2 fold change. Criteria for selection DEG: minimum expression level: 1;; minimum fold change: 2.0;; p--value (adjusted)<0.01.

781	CXCR2	-3.136707253	0.008797875
782	SDK2	-3.136135589	1.67E-06
783	ELMO3	-3.127160366	9.73E-21
784	MANSC1	-3.12456402	6.07E-30
785	FCGBP	-3.122029754	0.002526408
786	IP6K3	-3.117768499	0.002293633
787	METTL7B	-3.115145829	7.64E-09
788	SLC28A1	-3.11440709	7.70597E-04
789	TTC9	-3.109422011	1.92E-31
790	ZIC1	-3.102247632	9.30991E-04
791	HS3ST1	-3.095236955	1.31E-14
792	MIPOL1	-3.091982156	3.41E-19
793	SLC34A2	-3.087379735	0.001309578
794	ASS1	-3.083857121	1.34E-13
795	PRDM16	-3.083564076	0.005427056
796	KIAA1671	-3.072216352	8.07E-51
797	RHPN2	-3.060329196	5.64E-20
798	SCRT2	-3.060165005	2.67746E-04
799	KLC3	-3.055006863	7.96366E-04
800	LAMC2	-3.053374088	9.11E-24
801	ANKRD22	-3.05180232	7.28E-15
802	CHI3L1	-3.048852437	3.71E-07
803	SHROOM2	-3.048639205	9.64E-27
804	FAM107A	-3.046190278	0.003901524
805	ESRG	-3.045419018	3.92928E-04
806	SPAG17	-3.042208738	0.002516106
807	ANKS4B	-3.03948501	5.44044E-04
808	APOA2	-3.033998508	0.001246144

DEGs between the sarc/carc tumor component are shown in (A) and between the met/carc tumor component in (B) arranged by log2 fold change. Criteria for selection DEG: minimum expression level: 1;; minimum fold change: 2.0;; p--value (adjusted)<0.01.

809	C12orf36	-3.0318491	4.48824E-04
810	C8orf73	-3.031687386	4.40E-08
811	PDK4	-3.030298523	1.32E-09
812	XDH	-3.03015631	4.58E-16
813	PBOV1	-3.022735633	6.46E-07
814	SCN2B	-3.019918821	0.008022846
815	PTGS2	-3.015737681	1.44E-07
816	GDPD2	-3.013637553	0.002394775
817	SBSN	-3.011793261	0.004648018
818	HMGN5	-3.011166872	0.00081784
819	GOLGA7B	-3.001049787	3.23E-06
820	GLDN	-2.998260966	2.50691E-04
821	BMP4	-2.998152945	3.44E-09
822	MEGF6	-2.996620156	2.55E-20
823	LOC100507003	-2.994266429	0.002369062
824	CELF3	-2.994226893	0.003670138
825	FLJ43879	-2.993696368	9.66E-05
826	EPB41L4B	-2.976694592	2.76E-15
827	ALPK3	-2.972251805	4.46731E-04
828	LOC100188947	-2.966137961	0.006165703
829	GOLGA6L6	-2.9648073	0.001636739
830	AGMO	-2.964214431	7.56E-09
831	UPB1	-2.951933835	0.004224158
832	CHRNB4	-2.951798911	0.00647654
833	RASGRF1	-2.950027186	0.003594821
834	TMEM169	-2.94578491	1.65879E-04
835	OTOGL	-2.945208738	0.001586167
836	CXCL3	-2.944112496	0.0004375

DEGs between the sarc/carc tumor component are shown in (A) and between the met/carc tumor component in (B) arranged by log2 fold change. Criteria for selection DEG: minimum expression level: 1;; minimum fold change: 2.0;; p--value (adjusted)<0.01.

837	GOLGA6L1	-2.936856319	7.88876E-04
838	SLC26A9	-2.932288127	0.002116968
839	LOC100128593	-2.930750526	0.002661319
840	IL1R2	-2.930223067	9.95871E-04
841	TNFRSF11A	-2.927101229	7.29E-06
842	A2ML1	-2.926291322	5.32E-11
843	LOC100131176	-2.920366953	0.008576474
844	PRRG2	-2.920123908	5.64563E-04
845	PLIN5	-2.916645332	4.71E-05
846	KIF12	-2.908583935	1.36E-08
847	LOC100133957	-2.907118361	0.005299688
848	KIAA1804	-2.904593241	1.40E-12
849	CORO2A	-2.903906187	5.96E-32
850	DGCR5	-2.903898862	0.002428405
851	VILL	-2.901010812	7.50E-19
852	KRT9	-2.89710938	5.04264E-04
853	TMPRSS3	-2.893856208	8.02E-08
854	SFRP1	-2.893114914	2.85E-06
855	SLCO4A1	-2.892596255	6.33E-14
856	RAB27B	-2.891078338	9.20E-18
857	ANK1	-2.890922669	3.83E-28
858	TFAP2B	-2.884850639	0.009227079
859	LOC100129794	-2.884779071	0.00486304
860	EFCAB6	-2.88449583	0.008764394
861	C9orf50	-2.883170084	0.009286198
862	SYT12	-2.882012003	0.001603772
863	C15orf48	-2.870888492	1.30E-09
864	IL1A	-2.86874216	7.18E-06

DEGs between the sarc/carc tumor component are shown in (A) and between the met/carc tumor component in (B) arranged by log2 fold change. Criteria for selection DEG: minimum expression level: 1;; minimum fold change: 2.0;; p--value (adjusted)<0.01.

865	LOC283299	-2.868525199	4.28E-07
866	MALL	-2.866825315	4.03E-23
867	CCDC108	-2.859273215	0.00404104
868	FLJ23867	-2.85757468	3.32E-21
869	IGSF9	-2.857545	6.51E-07
870	ADAM23	-2.855883419	4.88E-09
871	PGBD5	-2.845060371	5.15E-05
872	PHEX	-2.842537956	0.002652819
873	CCDC68	-2.839374236	4.79E-19
874	MUC20	-2.834749021	1.36E-06
875	EEF1DP3	-2.830132606	0.004830813
876	RNF208	-2.829998148	0.001509889
877	RAPGEF3	-2.829974358	3.27E-14
878	GPR111	-2.828807615	4.90997E-04
879	PLLP	-2.828660148	8.56E-06
880	FAM164C	-2.827802232	2.63025E-04
881	LRAT	-2.824830602	6.91884E-04
882	MARVELD3	-2.821518192	1.71355E-04
883	DES	-2.818056895	7.44E-07
884	C17orf55	-2.815309064	1.70E-05
885	SLC4A11	-2.814063307	0.00454349
886	TMEM213	-2.810340597	0.008408536
887	ANKRD20A5P	-2.809853696	1.00E-08
888	CPXM2	-2.809244868	1.95E-06
889	SYT3	-2.808797714	0.007730313
890	LEP	-2.804998554	9.15247E-04
891	IGFN1	-2.803078484	8.04E-05
892	ATP1B1	-2.800525292	9.06E-47

DEGs between the sarc/carc tumor component are shown in (A) and between the met/carc tumor component in (B) arranged by log2 fold change. Criteria for selection DEG: minimum expression level: 1;; minimum fold change: 2.0;; p--value (adjusted)<0.01.

893	PITX2	-2.796985539	0.006093431
894	SLC6A1	-2.790322241	0.007551246
895	SCN4B	-2.789592637	0.00187004
896	FLJ22184	-2.789553542	8.17471E-04
897	ASGR1	-2.788562322	4.94178E-04
898	DUSP4	-2.785553715	3.96E-30
899	RIPK4	-2.782077762	1.88E-16
900	BFSP1	-2.781592645	1.87E-06
901	NOXO1	-2.771931198	7.07491E-04
902	LNX1	-2.771244262	1.62E-10
903	FAM201A	-2.769138291	0.001352134
904	CXCL1	-2.763440321	4.06E-05
905	MYH3	-2.761906875	0.003210695
906	TCHH	-2.761553451	1.49304E-04
907	ELOVL2	-2.758383341	0.003686901
908	NCCRP1	-2.740960565	2.24E-05
909	GAP43	-2.739924769	0.001111967
910	VSIG1	-2.73773149	1.87E-06
911	TSPAN12	-2.736977742	2.40E-06
912	GCOM1	-2.736757368	3.34E-09
913	NLGN4X	-2.735282886	9.40327E-04
914	SLC47A1	-2.731613584	7.21E-05
915	TPD52L1	-2.723512075	7.84E-05
916	PPM1H	-2.720895364	2.90E-07
917	MSI1	-2.716794258	0.006215603
918	ATP2C2	-2.714302681	2.40E-08
919	LONRF2	-2.712201386	1.30E-16
920	C5	-2.708956056	6.52E-16

DEGs between the sarc/carc tumor component are shown in (A) and between the met/carc tumor component in (B) arranged by log2 fold change. Criteria for selection DEG: minimum expression level: 1;; minimum fold change: 2.0;; p--value (adjusted)<0.01.

921	ZNF185	-2.702188712	5.91E-32
922	FAM131B	-2.702131588	0.008409208
923	KRTCAP3	-2.700701728	3.80E-08
924	DYX1C1	-2.699183719	0.004092083
925	PDE11A	-2.69880968	1.84E-08
926	GPR37	-2.696999534	1.84831E-04
927	ITGB6	-2.694709947	1.14E-07
928	THSD7A	-2.694140831	9.14E-08
929	STEAP1	-2.693939444	1.72E-13
930	THRB	-2.689711689	7.04E-10
931	PON3	-2.688916153	1.26E-05
932	LRRN1	-2.687519439	4.69304E-04
933	SYN1	-2.686720143	2.94E-05
934	KLF5	-2.672023563	7.49E-36
935	PVRL4	-2.671946336	9.98E-25
936	PKP3	-2.669663989	6.24E-21
937	FERMT1	-2.662313538	1.39E-18
938	SPNS2	-2.660786157	5.79E-06
939	TOB1	-2.659231218	7.46E-25
940	DCLK3	-2.655996189	0.002120197
941	CYLC2	-2.655516067	0.003137124
942	BMP3	-2.653321254	0.007685046
943	TSPAN15	-2.652206487	6.16E-27
944	CDO1	-2.648798484	0.007179514
945	LOC442132	-2.646832041	0.002496972
946	F12	-2.641218581	0.007485031
947	LRRC1	-2.639111787	1.69E-18
948	FBP1	-2.634624553	3.39E-10

DEGs between the sarc/carc tumor component are shown in (A) and between the met/carc tumor component in (B) arranged by log2 fold change. Criteria for selection DEG: minimum expression level: 1;; minimum fold change: 2.0;; p--value (adjusted)<0.01.

949	F3	-2.630047659	1.02E-19
950	FLJ12825	-2.629635076	0.008311235
951	DOCK3	-2.628145414	0.004278965
952	BAIAP3	-2.62458892	1.33E-10
953	GRB7	-2.624422282	2.25E-19
954	LYPD3	-2.62306355	8.69147E-04
955	TNFRSF21	-2.616134009	4.92E-38
956	TRPV4	-2.613452455	3.16E-07
957	PITPNM3	-2.612609278	3.65E-14
958	FANK1	-2.612353698	7.15E-06
959	CLU	-2.609086782	7.49E-06
960	TGM2	-2.608675905	8.56E-08
961	NOSTRIN	-2.607115665	2.04E-08
962	C1orf210	-2.602679168	0.005714628
963	C2CD4A	-2.602653296	6.57E-10
964	TSPAN2	-2.596818756	5.25E-07
965	OXTR	-2.594735068	7.76E-22
966	LOC100128675	-2.592946058	0.009893142
967	TMTC2	-2.582078612	1.06E-18
968	ARHGAP40	-2.581933727	2.95E-06
969	ABCB4	-2.581318921	2.86992E-04
970	PARD6B	-2.580547217	1.51E-16
971	PALMD	-2.579745726	3.42E-08
972	DSCR6	-2.572340021	0.002607112
973	ENTPD3	-2.571402163	0.005613678
974	TM4SF1	-2.571017321	3.63E-40
975	PNMA2	-2.570806339	3.09387E-04
976	MYADML	-2.57066252	0.008321066

DEGs between the sarc/carc tumor component are shown in (A) and between the met/carc tumor component in (B) arranged by log2 fold change. Criteria for selection DEG: minimum expression level: 1;; minimum fold change: 2.0;; p--value (adjusted)<0.01.

977	GIPC2	-2.56582262	4.34E-05
978	SLC12A2	-2.564726382	3.18E-28
979	ABAT	-2.559250048	9.88E-09
980	GABRB3	-2.558054007	2.84E-07
981	FSIP2	-2.552922599	4.55E-08
982	LOC100128338	-2.549359123	4.38E-13
983	ANXA3	-2.542557434	1.33E-24
984	REPS2	-2.541682944	5.33E-15
985	BCL2L15	-2.540090535	2.52E-06
986	GRAMD1C	-2.539785403	9.34E-07
987	CNKSR1	-2.537725835	4.80E-11
988	STOX2	-2.533058805	1.03E-05
989	GPRC5A	-2.530264749	2.91E-27
990	HES1	-2.52972861	8.51E-10
991	C16orf74	-2.526622498	1.50E-06
992	PLA2G16	-2.517385776	2.01E-24
993	CYP3A5	-2.517005855	7.11E-24
994	RPS6KA2	-2.516984713	5.05E-28
995	FUT1	-2.513674137	4.69421E-04
996	WSCD1	-2.509208191	9.36829E-04
997	TSPAN13	-2.507836382	8.13E-07
998	MYH15	-2.489880251	4.11024E-04
999	SHROOM3	-2.488335868	8.46E-35
1000	PTGES	-2.478663698	2.28E-05
1001	VWDE	-2.478287689	1.28E-14
1002	PRKCZ	-2.477193484	3.85E-06
1003	PLAC8	-2.475195057	4.87E-05
1004	SERPINE2	-2.472302194	3.10E-09

DEGs between the sarc/carc tumor component are shown in (A) and between the met/carc tumor component in (B) arranged by log2 fold change. Criteria for selection DEG: minimum expression level: 1;; minimum fold change: 2.0;; p--value (adjusted)<0.01.

1005	RASD1	-2.469921175	0.002940463
1006	MESTIT1	-2.468420408	6.55026E-04
1007	RAB11FIP1	-2.467741387	1.23E-11
1008	XIRP1	-2.463361226	0.004659413
1009	C1orf126	-2.462833436	2.15E-05
1010	OCLN	-2.46173882	5.96E-32
1011	C10orf11	-2.450970533	0.001691373
1012	FMN1	-2.449190656	1.83E-33
1013	TMEM45A	-2.447498969	2.17E-11
1014	SLC13A5	-2.44652869	3.02E-07
1015	ERP27	-2.445121643	0.007179514
1016	NGEF	-2.44489795	4.06E-14
1017	PTPRD	-2.439188455	3.11E-05
1018	FOXQ1	-2.435642979	8.09E-09
1019	ATOH8	-2.433738825	0.005022887
1020	LOC100128531	-2.432140738	0.001583608
1021	GPRC5C	-2.424192644	2.07E-10
1022	SIRPB1	-2.423427169	0.005812644
1023	FAM108C1	-2.420291476	3.95E-18
1024	UBD	-2.419218426	9.07E-11
1025	RASSF7	-2.413932577	1.01E-18
1026	MPV17L	-2.413382873	0.001307602
1027	PCDP1	-2.410886169	0.008920715
1028	RGN	-2.410800826	0.006976387
1029	LOC100127888	-2.409260294	0.001973658
1030	CHST6	-2.408389626	8.26E-11
1031	HYDIN	-2.406611016	0.001525287
1032	TMEM184A	-2.406373188	1.73E-07

DEGs between the sarc/carc tumor component are shown in (A) and between the met/carc tumor component in (B) arranged by log2 fold change. Criteria for selection DEG: minimum expression level: 1;; minimum fold change: 2.0;; p--value (adjusted)<0.01.

1033	NOXA1	-2.406327674	7.54E-08
1034	FAM133A	-2.40359853	0.002926856
1035	LOC728643	-2.39535594	0.003264395
1036	ADRA1A	-2.395272068	0.00202459
1037	SORL1	-2.395086185	1.41E-21
1038	CA2	-2.391850555	0.005359767
1039	RASSF9	-2.391522347	0.002602115
1040	GATA3	-2.383414789	5.15E-12
1041	CAPS	-2.38338732	2.53E-11
1042	LOC646324	-2.376940016	0.001838819
1043	FAM3C	-2.376813894	9.24E-27
1044	FST	-2.375454944	1.42034E-04
1045	SAMD12	-2.369102034	2.47E-15
1046	OSGIN1	-2.367267959	0.001569421
1047	GGT1	-2.364870957	5.78E-05
1048	LOC643650	-2.355927673	1.34E-06
1049	BHMT2	-2.353912894	4.43E-05
1050	COX6B2	-2.352914848	0.004499127
1051	EFHD1	-2.347539496	0.009389145
1052	EEPD1	-2.346911293	1.93E-17
1053	TFAP2C	-2.346676097	0.008797875
1054	SEMA3B	-2.345610315	7.89E-11
1055	NR5A2	-2.345208831	1.86E-06
1056	C17orf77	-2.344746452	0.009767155
1057	CHMP4C	-2.344291438	7.98E-12
1058	CATSPER1	-2.342293518	0.001147119
1059	ANGPT1	-2.334103908	0.003104073
1060	PRAM1	-2.332288027	0.001261239

DEGs between the sarc/carc tumor component are shown in (A) and between the met/carc tumor component in (B) arranged by log2 fold change. Criteria for selection DEG: minimum expression level: 1;; minimum fold change: 2.0;; p--value (adjusted)<0.01.

1061	HSPB6	-2.329587067	0.004158717
1062	ARRDC2	-2.322706267	2.20E-21
1063	NPNT	-2.319873556	8.46097E-04
1064	SH3YL1	-2.318646022	6.04E-10
1065	PARD6A	-2.317982406	0.008969882
1066	COL21A1	-2.316620354	4.17767E-04
1067	C5orf27	-2.315630617	0.001184295
1068	DDAH1	-2.315249449	4.80E-15
1069	MDFI	-2.314068595	7.99E-06
1070	SMAD6	-2.309664975	5.14E-05
1071	MUC6	-2.306985518	0.001916796
1072	EMCN	-2.302782933	0.0003001
1073	EGLN3	-2.302249368	1.06E-07
1074	C3orf15	-2.300706143	2.92532E-04
1075	PLEKHG6	-2.300585504	1.70E-10
1076	ST6GAL2	-2.29693454	4.91E-05
1077	WDR66	-2.292427037	1.48E-05
1078	SH2D3A	-2.288989403	7.48E-14
1079	MARVELD2	-2.288112776	7.25E-12
1080	LRRC56	-2.286770742	0.002051038
1081	NBEAL2	-2.282441008	6.99E-10
1082	AMIGO2	-2.278056067	1.93E-20
1083	FHDC1	-2.276622824	1.05E-08
1084	LOC439990	-2.276354237	8.29E-06
1085	AP1S3	-2.265917149	4.45E-25
1086	ARGFX	-2.264147583	0.005300664
1087	EPB41L1	-2.261474255	1.02E-13
1088	PADI2	-2.256743583	5.87E-06

DEGs between the sarc/carc tumor component are shown in (A) and between the met/carc tumor component in (B) arranged by log2 fold change. Criteria for selection DEG: minimum expression level: 1;; minimum fold change: 2.0;; p--value (adjusted)<0.01.

1089	PTPRR	-2.254978133	0.001855732
1090	UBXN10	-2.252485225	8.67E-08
1091	VAMP8	-2.250489141	1.25E-15
1092	P2RY1	-2.245649544	3.45645E-04
1093	C8orf55	-2.239263985	1.04E-05
1094	SLC37A1	-2.238339323	1.05E-10
1095	DTX4	-2.236429818	6.05E-19
1096	NET1	-2.234352405	8.60E-06
1097	SMPDL3B	-2.232119753	0.00013892
1098	IFITM10	-2.230095545	3.30032E-04
1099	DOK4	-2.229755866	1.79E-13
1100	ACPP	-2.223699281	9.29733E-04
1101	CYP2S1	-2.221892685	1.65523E-04
1102	RHPN1	-2.220527259	2.87E-06
1103	MREG	-2.217530945	0.005655516
1104	TNNI1	-2.210400369	0.001107838
1105	CRIP1	-2.209551293	2.23E-12
1106	FRRS1	-2.202404489	0.004812966
1107	PTK6	-2.200009356	2.80E-17
1108	SNCA	-2.198466815	0.004512499
1109	TC2N	-2.189426366	6.97E-10
1110	TMPRSS6	-2.187435215	3.25201E-04
1111	JAG2	-2.1873407	2.51E-05
1112	DMBX1	-2.184834983	5.02523E-04
1113	ABCC6	-2.181314271	9.03814E-04
1114	ITIH5	-2.180207201	5.57E-06
1115	PRRG4	-2.171478411	2.91E-10
1116	NYNRIN	-2.167933969	6.35E-11

DEGs between the sarc/carc tumor component are shown in (A) and between the met/carc tumor component in (B) arranged by log2 fold change. Criteria for selection DEG: minimum expression level: 1;; minimum fold change: 2.0;; p--value (adjusted)<0.01.

1117	PERP	-2.167075714	9.44E-17
1118	SC4MOL	-2.16233537	0.007739134
1119	STEAP2	-2.160943702	7.69E-26
1120	TST	-2.157264491	2.48E-05
1121	CXCL5	-2.15517206	6.91E-05
1122	FBXO2	-2.153232034	2.44E-05
1123	EZR	-2.153196245	7.10E-36
1124	FAAH2	-2.149208039	3.63116E-04
1125	SYTL1	-2.148094108	2.78044E-04
1126	ALDH1A3	-2.146467948	2.19E-13
1127	PLA2G4A	-2.14192728	0.001501155
1128	CHN2	-2.139696381	6.71E-07
1129	RASL10B	-2.139259957	0.009873125
1130	SHC3	-2.13899609	8.96E-08
1131	ALDH4A1	-2.136995718	2.05E-06
1132	SOX5	-2.136926061	5.88707E-04
1133	CXCL6	-2.13437819	0.004725279
1134	C16orf46	-2.130594066	7.65381E-04
1135	ANXA1	-2.129663556	2.91E-20
1136	CHGB	-2.125488906	0.002653059
1137	ENPP4	-2.120210252	8.36E-09
1138	CD300E	-2.115172116	0.003688027
1139	LOC219731	-2.111306609	1.38169E-04
1140	IL20RA	-2.110030495	1.14E-08
1141	KAZN	-2.109894454	0.003103316
1142	KIAA1324L	-2.103344681	1.09E-05
1143	AMOT	-2.103034328	5.25E-07
1144	LDLR	-2.101014694	1.96E-07

DEGs between the sarc/carc tumor component are shown in (A) and between the met/carc tumor component in (B) arranged by log2 fold change. Criteria for selection DEG: minimum expression level: 1;; minimum fold change: 2.0;; p--value (adjusted)<0.01.

1145	C15orf41	-2.0926056	1.38E-07
1146	RNF39	-2.091956078	0.005242984
1147	TMC6	-2.091213525	4.77E-12
1148	SH2D4A	-2.090500888	9.55E-14
1149	ATP2A1	-2.09017249	0.008077389
1150	ZNF860	-2.088233928	0.00011249
1151	CYP8B1	-2.088075341	0.00224447
1152	FUT6	-2.086218934	0.004694721
1153	CCDC113	-2.084101625	0.004975104
1154	C9orf140	-2.075917942	0.00043551
1155	CXCL16	-2.074973692	4.08E-19
1156	EXPH5	-2.074692382	3.58E-13
1157	GALNT12	-2.073469668	5.60E-15
1158	KCNK5	-2.067958451	1.05751E-04
1159	SYNGR2	-2.065690591	4.10E-22
1160	C9orf68	-2.049578477	3.76078E-04
1161	SVIP	-2.048234396	7.30E-07
1162	EPDR1	-2.047373698	4.05E-10
1163	TFAP2A	-2.043405591	5.92E-13
1164	SMPD3	-2.042904254	0.003127337
1165	NUP62CL	-2.038658449	0.003945185
1166	PAQR4	-2.035327553	4.67E-07
1167	ACSL5	-2.032557059	1.81E-17
1168	TRIM47	-2.032212067	4.10E-09
1169	FAM70A	-2.028367677	0.00586411
1170	GINS2	-2.021775283	4.90E-06
1171	LAMA1	-2.019265628	5.50638E-04
1172	DHRS3	-2.017600542	8.40E-08

DEGs between the sarc/carc tumor component are shown in (A) and between the met/carc tumor component in (B) arranged by log2 fold change. Criteria for selection DEG: minimum expression level: 1;; minimum fold change: 2.0;; p--value (adjusted)<0.01.

1173	MGST2	-2.016220706	1.08E-06
1174	SIGLEC11	-2.012142422	0.008146439
1175	LOC100507421	-2.0113161	0.007345246
1176	ALDH5A1	-2.007323507	8.75E-06
1177	C9orf150	-2.002656676	1.23E-13
1178	CTSK	2.000939377	1.13E-11
1179	LOC645166	2.008222122	7.63E-12
1180	MICAL2	2.010276265	2.19E-18
1181	SPARCL1	2.01482964	2.43647E-04
1182	CD74	2.016931594	4.30E-12
1183	PFN2	2.018833759	4.88E-21
1184	WIPF1	2.019287022	1.80E-22
1185	GJA1	2.022188474	8.49E-14
1186	CXCL10	2.025575018	2.19E-05
1187	FAM26E	2.025704493	1.55E-07
1188	CYBB	2.02735609	1.80E-05
1189	MGAT5B	2.027512783	1.77E-07
1190	CYTIP	2.02797986	8.15E-05
1191	IKZF1	2.031259466	2.46E-05
1192	ODZ4	2.033686436	1.11E-20
1193	PLEKHG4	2.036321067	2.05E-13
1194	LILRB4	2.041056481	8.30E-10
1195	RHOQ	2.042236395	2.83E-26
1196	CASP3	2.044447821	3.79E-21
1197	DENND2A	2.046683018	2.21E-15
1198	DNM1	2.048989777	5.83E-06
1199	IL31RA	2.050354303	4.60E-19
1200	TGFBR1	2.054854782	4.92E-15

DEGs between the sarc/carc tumor component are shown in (A) and between the met/carc tumor component in (B) arranged by log2 fold change. Criteria for selection DEG: minimum expression level: 1;; minimum fold change: 2.0;; p--value (adjusted)<0.01.

1201	POU2F2	2.055188958	3.55E-08
1202	PDE4B	2.061212295	5.99E-05
1203	ERAP2	2.068437089	1.23E-22
1204	GPR137C	2.068731043	1.65585E-04
1205	EPHA3	2.070365563	2.07E-05
1206	HLA-DPB1	2.078451424	2.03E-07
1207	XBP1	2.080281146	0.004478352
1208	FASLG	2.081842597	8.66774E-04
1209	DCN	2.090031055	1.20E-07
1210	C1QB	2.092053836	1.23E-05
1211	MS4A6A	2.094314576	5.48904E-04
1212	LOC100507463	2.094417414	1.61556E-04
1213	TP53INP1	2.096179069	0.00026674
1214	ADAMTS14	2.099723472	1.58E-12
1215	IL2RB	2.104413431	1.77E-11
1216	FGF5	2.10740682	1.23E-24
1217	THEMIS	2.114677688	1.62E-06
1218	LRMP	2.116322149	5.90306E-04
1219	LY96	2.118839911	0.006153375
1220	KCNJ15	2.125096735	7.01E-09
1221	LAX1	2.1259369	0.002031675
1222	NRXN3	2.126105035	8.25E-18
1223	C13orf33	2.130901697	4.79E-12
1224	SH2D1A	2.135793573	3.16E-05
1225	GGTA1P	2.139127929	0.001402534
1226	HLA-DRA	2.139997611	5.50E-08
1227	ABCD2	2.14059856	0.00457534
1228	SNED1	2.141294188	4.50E-18

DEGs between the sarc/carc tumor component are shown in (A) and between the met/carc tumor component in (B) arranged by log2 fold change. Criteria for selection DEG: minimum expression level: 1;; minimum fold change: 2.0;; p--value (adjusted)<0.01.

1229	C1QA	2.14265195	3.56E-05
1230	NEURL1B	2.145462626	3.35E-07
1231	SGK1	2.146146969	3.53E-18
1232	LEPREL1	2.149135892	2.49E-13
1233	EGR1	2.151545914	1.12E-07
1234	AIM2	2.153695109	0.004456735
1235	SLC2A5	2.155212336	3.45E-08
1236	SLAMF7	2.155731254	3.14933E-04
1237	CNIH3	2.159104743	2.31E-10
1238	ANTXR1	2.160818676	7.37E-17
1239	CHN1	2.163435498	1.23E-09
1240	GPN1	2.182152621	6.27E-18
1241	SLC9A10	2.183427521	5.29E-08
1242	RNF150	2.184618168	1.21E-09
1243	GIMAP2	2.188381813	0.001189844
1244	STMN3	2.18985698	3.46E-18
1245	FAM171A1	2.190300384	1.69E-14
1246	SIGLEC1	2.192722311	3.99E-07
1247	ARID5A	2.197117861	2.70E-08
1248	SYNGR3	2.204226928	0.001965923
1249	KIAA1462	2.205933828	1.17E-28
1250	PRICKLE1	2.206476433	1.26E-23
1251	ABCA10	2.21122391	8.19813E-04
1252	GLIPR1	2.217850376	7.23E-17
1253	FABP5	2.219875272	7.93E-14
1254	COL8A2	2.220995735	6.06E-12
1255	KIAA0125	2.226492807	0.001441537
1256	CD96	2.229442842	9.65E-07

DEGs between the sarc/carc tumor component are shown in (A) and between the met/carc tumor component in (B) arranged by log2 fold change. Criteria for selection DEG: minimum expression level: 1;; minimum fold change: 2.0;; p--value (adjusted)<0.01.

1257	STX1B	2.234165082	4.37E-11
1258	ITM2A	2.239976058	4.11743E-04
1259	SMAD1	2.241275004	1.40E-23
1260	IL10RA	2.243519711	8.45E-08
1261	DIXDC1	2.244106649	9.11E-24
1262	PTGER2	2.247933274	4.17E-05
1263	CD48	2.248575057	7.21E-05
1264	BATF3	2.25177026	0.001503255
1265	PRF1	2.257581977	9.40E-07
1266	APCDD1L	2.259449727	5.85344E-04
1267	PTN	2.259603863	1.57E-07
1268	TGFB3	2.266380195	1.08E-20
1269	VAMP5	2.270015869	1.10E-07
1270	HLA-DPA1	2.270399294	5.16E-08
1271	OLFML2B	2.275393934	1.46E-14
1272	PLXNC1	2.278571871	1.18E-10
1273	ITPKA	2.280672589	4.96E-05
1274	PRUNE2	2.287068511	6.37E-15
1275	SECTM1	2.290192354	6.28E-06
1276	CD8A	2.295198224	7.91E-05
1277	FZD8	2.297924489	2.53E-05
1278	TNFSF13B	2.298047265	1.20E-07
1279	CPE	2.29935139	1.33E-06
1280	STARD9	2.309546144	2.16E-30
1281	KCNG1	2.315865268	0.00137983
1282	RAB31	2.316120752	9.35E-26
1283	SYNC	2.317427931	0.00011249
1284	C6orf174	2.319457537	6.29E-08

DEGs between the sarc/carc tumor component are shown in (A) and between the met/carc tumor component in (B) arranged by log2 fold change. Criteria for selection DEG: minimum expression level: 1;; minimum fold change: 2.0;; p--value (adjusted)<0.01.

1285	PTPRC	2.320254379	1.04E-05
1286	FBXO32	2.327233189	8.43E-30
1287	TRPC1	2.32798665	3.30E-17
1288	CD163	2.33133606	1.94E-09
1289	NF2	2.331749337	1.88E-30
1290	IL4I1	2.337654189	1.25E-07
1291	ACPL2	2.343223386	2.49E-20
1292	CCND2	2.352804846	3.21E-13
1293	PDZD2	2.355019129	2.79E-25
1294	TMOD2	2.366704649	2.25E-27
1295	OLFML3	2.369419331	2.02E-09
1296	PTPRS	2.374349024	1.23E-18
1297	LOC643401	2.381614641	6.21E-18
1298	IGFBP5	2.382475049	1.35E-14
1299	DAB2	2.397109138	3.69E-35
1300	VWF	2.401024312	3.07E-07
1301	PRRX1	2.408600031	2.32E-20
1302	SPDYA	2.431769789	0.001203393
1303	ITK	2.434684539	7.20E-06
1304	CNRIP1	2.445400447	2.71E-06
1305	TRIM9	2.459002072	1.98E-14
1306	TGFBI	2.460672365	7.58E-31
1307	E2F7	2.46556575	1.84E-12
1308	RGMB	2.472318925	7.78E-28
1309	LRIG1	2.485407727	1.62E-25
1310	TMEM158	2.497516508	1.46E-11
1311	NKG7	2.499611153	6.02E-06
1312	OLFML2A	2.506459902	2.05E-35

DEGs between the sarc/carc tumor component are shown in (A) and between the met/carc tumor component in (B) arranged by log2 fold change. Criteria for selection DEG: minimum expression level: 1;; minimum fold change: 2.0;; p--value (adjusted)<0.01.

1313	RBMS3	2.51139113	2.95E-27
1314	PLTP	2.512057985	2.18E-21
1315	MEG3	2.513044816	2.38E-09
1316	MIR100HG	2.524640894	4.16E-18
1317	SACS	2.53601437	3.42E-45
1318	CORO2B	2.558308281	3.42E-11
1319	GLI3	2.570868551	1.08E-27
1320	PAEP	2.580913152	4.53E-08
1321	GBP1	2.593025926	9.33E-09
1322	SLC9A9	2.597290947	1.94E-09
1323	SPEG	2.623923745	2.93E-10
1324	BNC2	2.63825999	3.49E-40
1325	FMO2	2.638903987	1.58E-05
1326	UNC5A	2.639980187	3.51E-08
1327	PDCD1LG2	2.654781839	3.21E-09
1328	GLIPR2	2.676278094	5.96E-17
1329	CREB5	2.677427058	1.09E-43
1330	H19	2.680603245	0.00013105
1331	EPB41L3	2.685908944	1.31E-28
1332	IKZF3	2.688995716	2.42E-05
1333	CD200	2.689835156	1.47E-12
1334	TRAT1	2.693898531	0.004438055
1335	ARHGAP15	2.695477854	4.28E-07
1336	PROCR	2.695583472	7.80E-25
1337	SCG5	2.700744247	3.94E-15
1338	F13A1	2.714957853	3.78E-05
1339	IRF4	2.717178577	4.38E-05
1340	VEGFC	2.717541198	4.05E-27

DEGs between the sarc/carc tumor component are shown in (A) and between the met/carc tumor component in (B) arranged by log2 fold change. Criteria for selection DEG: minimum expression level: 1;; minimum fold change: 2.0;; p--value (adjusted)<0.01.

1341	POPDC3	2.727130308	0.00031406
1342	IL7R	2.730510414	3.78E-10
1343	PCDHGA3	2.737114409	3.41E-08
1344	LTBP1	2.741334761	8.21E-53
1345	SRPX	2.746264579	1.24E-19
1346	LOC84856	2.759350219	6.75E-06
1347	ADAMDEC1	2.759822548	8.03E-05
1348	MFAP2	2.769076204	7.99E-10
1349	VASH2	2.775654225	3.69E-17
1350	CD2	2.784521993	2.53E-06
1351	BTG2	2.791840047	4.12E-16
1352	ITGB3	2.800901295	3.06E-45
1353	RASGEF1C	2.820222654	0.007766072
1354	PAPPA	2.833378962	5.65E-50
1355	CD38	2.842581916	0.006859988
1356	PYHIN1	2.848861824	3.64E-05
1357	GLIS1	2.851294249	2.61E-07
1358	CCL4	2.865947437	3.24E-05
1359	ARHGAP20	2.86826587	5.72E-18
1360	ADAMTS16	2.869051132	5.96E-19
1361	PTPN22	2.876123476	4.72E-24
1362	CHST2	2.878529506	3.38E-15
1363	MMP24	2.878665932	3.02E-35
1364	FMO1	2.891891759	7.14E-05
1365	FAM126A	2.896543143	3.35E-45
1366	SUSD5	2.943581232	1.87E-37
1367	PDPN	2.95726686	1.80E-32
1368	WNT5A	2.959279623	3.48E-47

DEGs between the sarc/carc tumor component are shown in (A) and between the met/carc tumor component in (B) arranged by log2 fold change. Criteria for selection DEG: minimum expression level: 1;; minimum fold change: 2.0;; p--value (adjusted)<0.01.

1369	DERL3	2.967869892	1.27E-11
1370	MAFB	2.986553598	1.82E-23
1371	EDIL3	3.014511346	1.01E-45
1372	FCRL5	3.018072218	2.94691E-04
1373	HRH2	3.021743272	5.05E-21
1374	GNLY	3.031098031	6.54E-15
1375	LOXL1	3.049679566	2.28E-28
1376	CCL21	3.073714508	1.37E-05
1377	NTNG1	3.076023954	1.06E-19
1378	CPNE7	3.099342642	1.46E-07
1379	NCAM1	3.10604038	1.68E-42
1380	BDKRB2	3.131601823	2.59E-26
1381	PTPRCAP	3.136373516	2.38E-05
1382	MOXD1	3.152747109	1.20E-16
1383	SPOCK3	3.162074296	2.98E-14
1384	NKD2	3.269361106	8.38E-13
1385	SLFN12L	3.299955313	9.76E-08
1386	HS3ST3A1	3.333422684	1.25E-09
1387	CD79A	3.342361947	3.00E-05
1388	SLC1A3	3.420850707	4.45E-45
1389	IL6	3.500268631	6.67E-06
1390	VAT1L	3.517991143	7.67E-19
1391	CXCL13	3.584918729	3.44E-06
1392	GBP5	3.586712972	3.70E-08
1393	CPA3	3.617433102	9.02E-05
1394	CYGB	3.648357561	1.17E-16
1395	FAM46C	3.648454895	4.27E-05
1396	ADAMTS6	3.656800008	3.12E-68

DEGs between the sarc/carc tumor component are shown in (A) and between the met/carc tumor component in (B) arranged by log2 fold change. Criteria for selection DEG: minimum expression level: 1;; minimum fold change: 2.0;; p--value (adjusted)<0.01.

1397	GZMK	3.823293949	1.14E-07
1398	PCDH9	3.865399475	1.44E-33
1399	SERPINE1	3.889982087	7.88E-29
1400	PIM2	3.910675467	4.60E-07
1401	SFRP2	3.941397389	4.69312E-04
1402	C7	4.053138236	9.15E-11
1403	IFNG	4.054486869	3.48E-05
1404	MIR155HG	4.065823945	1.09E-06
1405	APOD	4.169249832	3.80E-14
1406	GZMA	4.312252515	1.67E-13
1407	CXCL9	4.368525602	9.41E-10
1408	GFPT2	4.371841817	2.11E-64
1409	MIAT	4.374596181	4.66E-89
1410	GPR174	4.412101815	4.16E-07
1411	CLMP	4.795116161	2.32E-68
1412	ANKRD36BP2	4.99479114	2.17E-08
1413	DNER	5.322140304	4.30E-22
1414	MZB1	5.682279249	1.36E-07
1415	IGJ	6.014652406	6.63E-08
1416	MIR650	6.426255537	1.59175E-04
1417	SLN	6.525625335	2.02E-18
1418	IGLL5	6.682772456	2.72E-13

Supporting Table S3. Summary of immunohistochemical characterization of cholangiocarcinoma with sarcomatoid component and corresponding lymph node metastasis.

Antigen	Carcinomatous component	Sarcomatoid component	Lymph node metastasis
Keratin-7	+++	negative	negative
Keratin-8+18	+++	negative	negative
Keratin-19	+	negative	negative
Vimentin	+	+++	+++
c-Met	+++	+++	++
Claudin 4	+++	negative	negative
Muc1	+++*	negative	negative
E-cadherin	+++	negative	negative
Beta catenin	+++	+	+++ (nuclear staining)
CEA	+++	negative	negative
CD44	+++	+++	negative
P53	++	++	+++
Ki67	21.6±4.8	44.8±3.9	29.8±9.8
NCAM1	negative	negative	n.a.
CgA	negative	negative	n.a.
CD117	negative	negative	n.a.
CD34	negative	negative	n.a.
Nestin	+*	+	n.a.
EpCam	++	negative	negative
Hep Par 1	negative	negative	n.a.
AFP	negative	negative	n.a.

Samples showing staining of at least 10% of tumor cells were defined as positive, and the intensity of immunoreactivity was graded as weak (+), moderate (++), and strong (+++), heterogeneous or focal staining (*); not analyzed (n.a.). Ki67 reactivity was determined by counting positive cells in 10 high power fields.

Supporting Table S4. The most significantly differentially expressed genes between K7pos and K7neg sub-clones.

	downreulated genes in K7neg sub clones	Log2 fold change*	p-value (adjusted)	downregulated genes in K7pos sub clone	Log2 fold change*	p-value (adjusted)
1	LRRC61	-5.9	0.0000969	GALNT6	1	0.00062003
2	ACN9	-5.7	0.00062003	MPP4	1.1	0.0048732
3	C7orf29	-4.7	0.00364877	THSD7A	1.1	0.03266506
4	XKR6	-4.6	0.0000382	NRP1	1.1	0.00000077
5	LOC84856	-3.9	0.00830947	COL7A1	1.1	0.00557708
6	KRT7	-3.8	0.000048	FMNL3	1.1	0.00032817
7	FGA	-3.8	0.00032817	LEF1	1.2	0.00179461
8	LHFPL4	-2.9	0.00000000	NOV	1.2	0.00238779
9	C4BPA	-2.8	0.00315505	NOG	1.2	0.00993124
10	LRRN2	-2.8	0.04295868	EVI2A	1.2	0.00326579
11	COL21A1	-2.6	0.02951149	SFRP1	1.3	0.00000369
12	ZNF268	-2.5	0.00087405	FREM2	1.3	0.00676192
13	CXCL14	-2.2	0.0007305	IGDCC4	1.3	0.00393465
14	NID1	-2	0.0139443	IL24	1.5	0.00231568
15	AKR1B10	-2	0.00728353	CLDN3	1.6	0.00333562
16	UBD	-2	0.00167271	MNX1	1.6	0.00478243
17	OLFML3	-1.9	0.01147409	FAM26E	1.6	0.0000382
18	NKD2	-1.8	0.013417	ACSL5	1.7	0.00940214
19	TMEM233	-1.8	0.00174979	LOC149837	1.7	0.0049401
20	DAAM2	-1.8	0.00062003	VEPH1	1.7	0.0033163
21	RBP4	-1.7	0.000328	TRO	1.7	0.004655
22	C17orf107	-1.7	0.04002703	GJB3	1.8	0.00467597
23	EMILIN2	-1.7	0.03027566	DMBT1	1.9	0.02140321
24	ATOH8	-1.6	0.00112841	ADAM22	2.1	0.00000118
25	GSTM3	-1.6	0.0023611	MN1	2.2	0.00000025
26	SGK2	-1.5	0.01263831	HS3ST3A1	2.3	0.00465564
27	SGSM1	-1.5	0.0049401	IL13RA2	2.3	0.00032817
28	SECTM1	-1.5	0.00260028	INHBA	2.4	0.01788492
29	AKR1C3	-1.5	0.00084269	ZP3	2.5	0.00315505
30	EFCAB4B	-1.5	0.00018373	C20orf197	2.5	0.0020407
31	PTPN6	-1.4	0.00676192	NTM	2.6	0.00355414
32	RASL11A	-1.3	0.0000382	CPVL	3.3	0.00000000
33	CA12	-1.3	0.0000382	MGST1	3.5	0
34	MAPK13	-1.3	0.00062003	SSTR1	4.2	0.00063413
35	ADAP2	-1.3	0.00137982			
36	B3GALNT1	-1.2	0.00333562			

37	TNFSF10	-1.2	0,00279829			
38	PCSK9	-1.2	0,03150805			
39	SLC6A17	-1.2	0.00069297			
40	SIX2	-1.1	0.01911192			
41	WISP2	-1.1	0.03528742			
42	LOC644538	-1.1	0.02382562			
43	STK33	-1.1	0.00062003			
44	PPP2R2B	-1.0	0.01488498			

* The logarithm (to basis 2) of the fold change between samples ‘K7pos’ and ‘K7neg’. A negative log2 fold change designates that the gene is upregulated in K7pos sub clones and downregulated in K7neg sub clones, respectively. The opposite is true for the positive log2 fold change.

Supporting Abbreviations:

ZEB1, Zinc finger E-box binding homeobox 1

MCAM, Melanoma cell adhesion molecule

DSP, Desmoplakin

JUP, Junction plakoglobin

PVRL4, Nectin cell adhesion molecule 4

GJB3, Gap junction protein beta 3

GJB4, Gap junction protein beta 4

GJB6, Gap junction protein beta 6

TJP3, Tight junction protein 3

CGN, Cingulin

CDH1, Cadherin 1

CDH3, Cadherin 3

CRB3, Crumbs 3, cell polarity complex component

OCLN, Occludin

DDR1, Discoidin domain receptor tyrosine kinase 1

EPCAM, Epithelial cell adhesion molecule

CLDN3, Claudin 3

FGFR2, Fibroblast growth factor receptor 2

MMP2, Matrix metallopeptidase 2

MMP11, Matrix metallopeptidase 11

CDH2, Cadherin 2

VIM, Vimentin

SERPINE1, Serpin family E member 1

PDPN, Podoplanin

VEGFC, Vascular endothelial growth factor C

TLN2, Valin 2

TWIST1, Twist family bHLH transcription factor 1

TGFBR1, Transforming growth factor beta receptor 1

ZEB2, Zinc finger E-box binding homeobox 2

NCAM1, Neural cell adhesion molecule 1

PROCR, Protein C receptor

ANPEP, Alanyl aminopeptidase (membrane)

IL13RA2, Interleukin 13 Receptor Subunit Alpha 2

MUC-1, Mucin 1 Cell Surface Associated

CLDN4, Claudin 4

E-cadherin, Cadherin 1

c-MET, Hepatocyte Growth Factor Receptor

CEA, Carcinoembryonic antigen

CgA, Chromogranin A

CD117, KIT Proto-Oncogene Receptor Tyrosine Kinase

Hep Par 1, Hepatocyte Paraffin 1 antibody

AFP, Alpha fetoprotein

alpha-SMA, alpha smooth muscle actin
